# A Computational Framework for the Design and Development of Isoform Selective PI3Kα Inhibitors as Novel Anticancer Agents

**DOI:** 10.3390/molecules31162782

**Published:** 2026-08-10

**Authors:** Milan Jovanović, Teodora Djikic-Stojsic, Branislav Stanković, Marija Popovic-Nikolic, Katarina Nikolic

**Affiliations:** 1 Department of Pharmaceutical Chemistry, Faculty of Pharmacy, University of Belgrade, Vojvode Stepe 450, 11221 Belgrade, Serbia; teodora.djik88@gmail.com (T.D.-S.); marija.popovic@pharmacy.bg.ac.rs (M.P.-N.); 2 Department of Molecular Biology and Endocrinology, “VINCA” Institute of Nuclear Sciences—National Institute of the Republic of Serbia, University of Belgrade, Mike Petrovica Alasa 12–14, Vinca, 11351 Belgrade, Serbia; 3 Department of Nuclear and Plasma Physics, “VINCA” Institute of Nuclear Sciences—National Institute of the Republic of Serbia, University of Belgrade, Mike Petrovica Alasa 12–14, Vinca, 11351 Belgrade, Serbia; branislav.stankovic@vin.bg.ac.rs

**Keywords:** PI3Kα selective inhibitors, anticancer agents, scaffold hopping, 3D-QSAR, ensemble docking, CADD, DFT, ADMET

## Abstract

Background: Phosphatidylinositol 3-kinase (PI3K) is a promising anticancer drug target, and selective PI3Kα inhibition may provide both efficacy and an improved safety profile. This study aimed to design new potentially selective PI3Kα inhibitors using computer-aided drug design (CADD). Methods: Benzoxazepine and thiazole derivatives were investigated using molecular dynamics, ensemble docking, and Three-Dimensional Quantitative Structure–Activity Relationship (3D-QSAR) analyses. Scaffold hopping, substituent replacement, structure-based virtual screening, and density functional theory (DFT) calculations were then applied to guide the design and characterization of new derivatives. Results: The study identified new chemotypes capable of interacting with PI3Kα Val851 (αVal851) in the hinge region, including chromeno[3,4-d]imidazole, 2H-benzo[b]oxazine, and quinoline derivatives. Additional substructures directed toward hydrophobic region II and the αGln859 interaction environment supported predicted selectivity over PI3Kβ, PI3Kγ, and PI3Kδ. Conclusions: The results establish a comprehensive CADD framework for the rational design of selective PI3Kα inhibitors and provide new compounds with improved predicted selectivity profiles for further development.

## 1. Introduction

Phosphatidylinositol 3-kinases (PI3K) are lipid kinases that regulate numerous cellular processes, such as proliferation, cell metabolism, motility, and apoptosis. The PI3K enzyme catalyzes the phosphorylation of inositol phospholipids at position 3 of inositol in response to extracellular stimuli, producing phosphatidylinositol 3,4,5-trisphosphate (PIP3) [1,2].

In mammals, PI3Ks are divided into three classes. Class I is further split into IA (PI3Kα, PI3Kβ, PI3Kδ) and IB (PI3Kγ). Class IA is activated by receptor tyrosine kinases, GPCRs, and oncoproteins, while Class IB is activated solely by GPCRs. Class IA includes PI3Kα, PI3Kβ, and PI3Kδ, encompassing enzymes with a p110 catalytic subunit (p110α, p110β, and p110δ) along with a regulatory subunit (p85) that manages receptor binding, activation, and the localization of the PI3K enzyme. Class IB includes PI3Kγ, which consists of the p110γ catalytic subunit bound to the p101 or p87 regulatory subunit. Unlike p110α and p110β, which are widely expressed, p110γ and p110δ are primarily expressed in leukocytes [1].

Common stimuli for this signaling pathway include growth factors like insulin-like growth factor (IGF) and chemokines. In the absence of activation signals, p85 interacts with p110 to inhibit its kinase activity across all four isoforms. Upon activation of the tyrosine kinase receptor or G-protein coupled receptor, p110 dissociates from the p85 subunit, binds to the membrane, and phosphorylates PIP2 to PIP3 [1,3].

**Figure 1:**
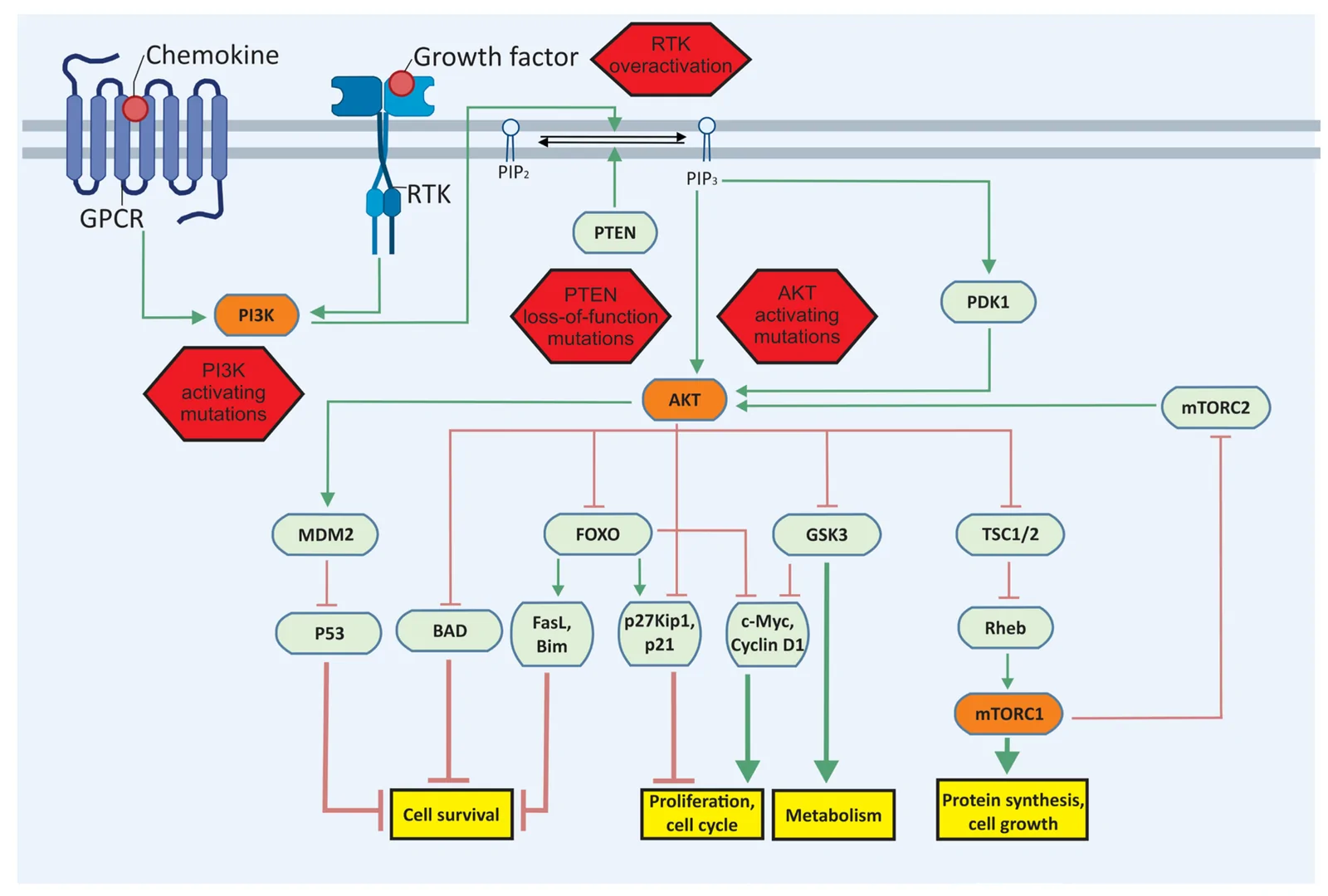
Schematic representation of PI3K signaling and its role in cancer development [1,8,9]; green lines represent activation effects, while orange lines represent inhibitory effects, and the grey parallel lines represent the cell membrane lipid bilayer.

Research on gene expression in cancer cells indicates that the PI3K signaling pathway (PI3K-AKT-mTOR) is most altered in human tumors (Figure 1). Among the four class I PI3K isoforms, only PIK3CA (the gene encoding the p110α subunit) is frequently mutated in human cancer, making it the second most mutated oncogene. In contrast, mutations in the catalytic subunits of other PI3K isoforms are rare. Additionally, the phosphatase and tensin homolog (PTEN) gene is among the most frequently mutated tumor suppressor genes, leading to increased activity in the PI3K-AKT-mTOR signaling pathway [1,4]. Since the activation of this pathway results in heightened enzyme activity, upregulation of the signaling cascade, and oncogenic transformation of cells, PIK3CA gene mutations are prevalent in many types of solid tumors (including brain, breast, colon, liver, stomach, and ovary) [1,5].

Targeting the PI3K enzyme has thus proven to be a promising strategy for developing new cancer therapies. By selectively targeting PI3Kα, anticancer effects on solid tumors can be achieved while minimizing side effects on the immune system, which relies on p110γ or p110δ [6]. Moreover, p110α plays an important role in angiogenesis, providing an additional advantage in cancer therapy [7].

**Figure 2:**
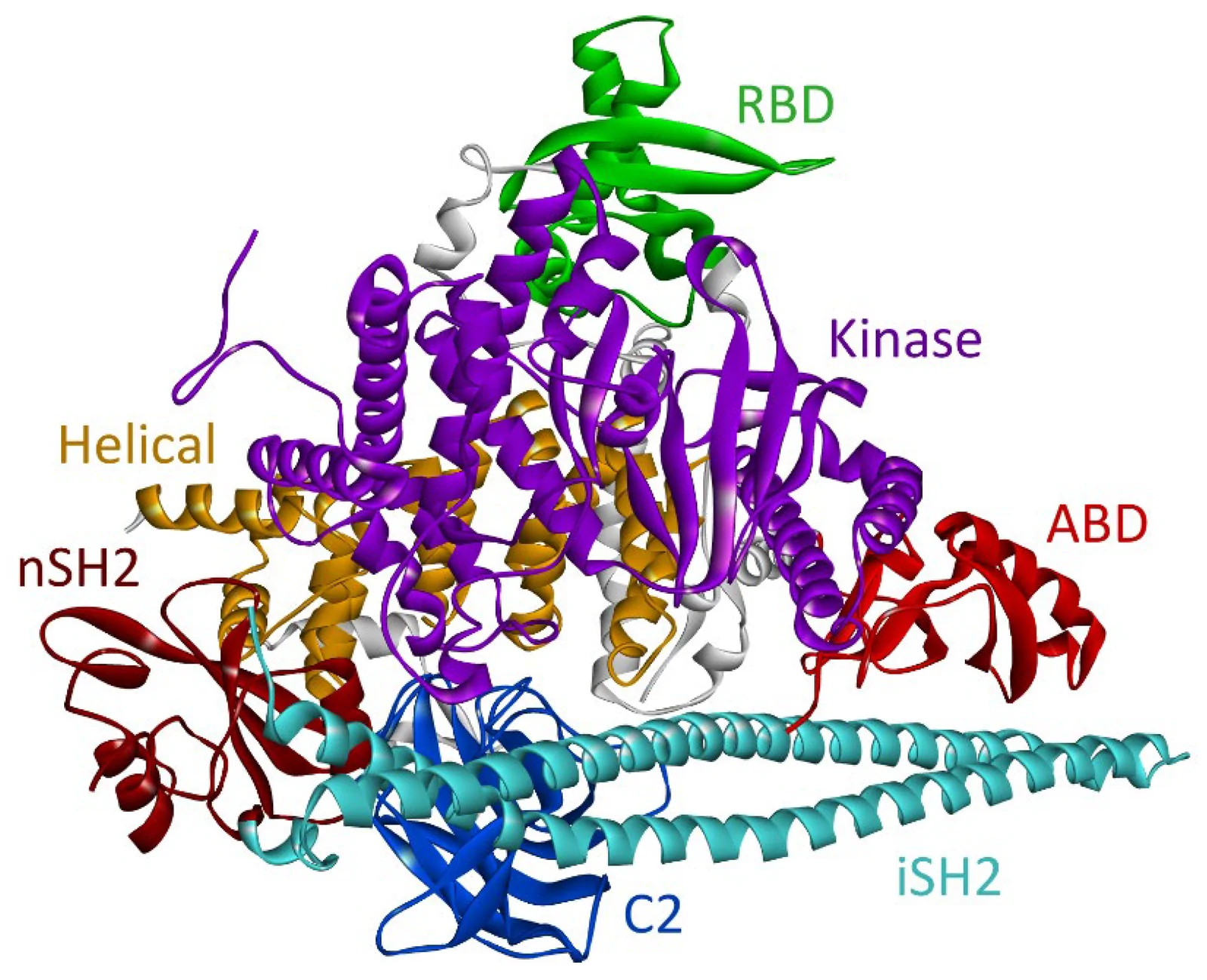
3D structure of the PI3Kα enzyme—p110 subunit: ABD, RBD, C2, helical and kinase domains; p85 subunit: nSH2 and iSH2 domains.

All class I p110 subunits of PI3K possess five domains: the adapter-binding domain (ABD), the Ras-binding domain (RBD), the C2 domain, the helical domain, and the kinase domain. Among these, the RBD, C2, helical and kinase domains show high sequence homology across isoforms. The p85 subunit is composed of five domains: Src homology-3-domain (SH3), GTPase-activating protein (GAP) and two SH2 domains (N-terminal nSH2 and C-terminal cSH2) separated by an inter-SH2 domain (iSH2) [10,11] (Figure 2).

The ATP-binding site of PI3K is at the junction of the two lobes of the kinase domain and contains five key features: the hinge region, affinity pocket, specificity pocket, hydrophobic region II (ribose pocket), and P-loop [12]. The hydrophobic region II (residues 852–860) and the P-loop (residues 768–781) show the greatest isoform variability, making them critical for designing selective inhibitors. A distinctive characteristic of kinase inhibitors is the presence of an articulated linker that mimics the adenine moiety of ATP and forms hydrogen bonds with conserved valines (αVal850/851 in PI3Kα). The affinity pocket, encircled by conserved residues, including αSer806, αLys833, αAsp841, αTyr867, and αAla885, is not accessible for ATP but may enhance potency when targeted, whereas the specificity pocket is blocked in PI3Kα, limiting inhibitor access.

Alpelisib (BYL719), a thiazole derivative, is an FDA-approved drug that acts as a selective inhibitor of the PI3Kα isoform [13]. This compound is approximately 50 times more potent against PI3Kα compared to other isoforms like PI3Kβ, γ, and δ. It is indicated for ER+/HER2−-advanced or metastatic breast cancer with a PIK3CA gene mutation. However, alpelisib may cause hyperglycemia as an adverse effect due to PI3Kα inhibition, but it still generally has a safer profile than pan-PI3K inhibitors. Hyperglycemia and other adverse effects, like rash and diarrhea, are typically manageable with monitoring. In contrast, pan-PI3K inhibitors often cause more frequent, severe, and varied toxicities, including some drug-specific adverse events. Given that mutations and hyperactivation of the PI3K-AKT-mTOR signaling pathway commonly contribute to resistance development in breast cancer, these drugs can be effectively combined with fulvestrant, an estrogen receptor antagonist [2,14,15]. Taselisib (GDC-0032) is another PI3K inhibitor that was undergoing phase III clinical trials. Chemically, it is a benzoxazepine derivative, exhibiting similar activity on the p110α, p110γ, and p110δ subunits, but reduced activity on the p110β subunit. The moderate efficacy of taselisib, combined with pronounced toxicity, led to the termination of further clinical studies for this inhibitor [16]. More recently, inavolisib (GDC-0077), also a benzoxazepine derivative, was approved in combination with palbociclib and fulvestrant for adults with endocrine-resistant, PIK3CA-mutated, hormone receptor (HR)-positive, human epidermal growth factor receptor 2 (HER2)-negative, locally advanced or metastatic breast cancer [17]. This compound demonstrates increased selectivity for the PI3Kα isoform compared to earlier benzoxazepine derivatives. Inavolisib is associated with some adverse reactions, including rash, dry skin, decreased appetite, and urinary tract infection, but these events are generally less severe compared to those observed with alpelisib [18].

Despite the advancement of PI3Kα selective inhibitors, current therapies are constrained by adverse effects and varying degrees of isoform selectivity. Adverse effects, like off-target gastrointestinal reactions due to inhibition of the PI3Kδ isoform [18], or on-target hyperglycemia and hyperinsulinemia, are major dose-limiting issues for PI3Kα inhibitors [19]. Additionally, many inhibitors display suboptimal efficacy or toxicity profiles in clinical trials [20]. Clinical responses to the therapy have been modest and, in many cases, insufficient to justify continuation of trials because of on-target toxicities (like hyperglycemia, liver enzyme elevation, GI symptoms, and immune-mediated toxicities). Thus, the identification and design of novel PI3Kα inhibitors with improved selectivity and therapeutic indices are needed.

Previous computational studies have combined molecular docking, virtual screening, 3D-QSAR, molecular dynamics, free-energy calculations, DFT, and ADMET prediction to identify potential PI3Kα inhibitors. However, their principal modeling was generally focused on the targeted PI3Kα isoform, with selectivity assessed through subsequent or more limited analyses [21,22,23]. In contrast, the present workflow used MD-derived receptor ensembles and separate 3D-QSAR models for PI3Kα, PI3Kβ, PI3Kγ, and PI3Kδ, enabling simultaneous evaluation of predicted target activity and potential off-target activity across all four class I isoforms.

This study aims to develop and validate an integrated platform for identifying or designing novel chemotypes of selective PI3Kα inhibitors as promising antineoplastic agents. Building on existing knowledge of PI3Kα inhibitors, this study focuses on the structural features and binding interactions of clinically relevant compounds—specifically thiazole and benzoxazepine derivatives. Insights gained from these scaffolds, including their PI3K isoform selectivity profiles and interaction patterns within key binding site regions, such as the hinge region, affinity pocket, hydrophobic region II, and P-loop, will guide the design of novel molecules. The ultimate objective is to develop new chemical entities with optimized binding to PI3Kα that overcome the limitations of current inhibitors. These efforts, supported by computer-aided drug design (CADD) techniques, aim to contribute to the development of the next generation of targeted therapies for cancers driven by PIK3CA mutations.

CADD tools have become crucial in modern drug discovery, facilitating the rapid identification and optimization of lead compounds through different in silico methods [24,25]. This study proposes an integrated approach that combines structure-based and ligand-based CADD methods to identify new promising compounds as potential next-generation PI3Kα inhibitors. These in silico strategies are particularly valuable when targeting conserved protein families like kinases, where small variations in binding pockets must be utilized to achieve isoform selectivity.

## 2. Results and Discussion

### 2.1. Prepared PI3K Structures and Ensemble Docking Protocol Validation

The human PI3Kβ structure was generated by homology modeling using the mouse PI3Kβ crystal structure as a template, due to the absence of a suitable experimental human PI3Kβ structure for the applied modeling workflow.

A multiple sequence alignment of the four human class I PI3K catalytic subunits was also considered in template selection. Human p110β shared approximately 58% sequence identity with p110δ, 42% with p110α, and 33% with p110γ, whereas approximately 96% identity was observed between human and mouse p110β. Although the alternative structures were of human origin, they belonged to different PI3K isoforms and therefore contained isoform-specific residues that may be relevant to ligand recognition and selectivity. Therefore, the same-isoform mouse p110β structure was preferred because it was expected to preserve β-specific sequence and structural features more reliably than a p110 subunit structure of human origin.

The selected model showed acceptable stereochemical and structural quality according to standard validation metrics, including zDOPE, Ramachandran plot analysis, ERRAT, and ProSA evaluation. Detailed validation parameters are provided in the Supplementary Information.

Initial docking of the representative benzoxazepine and thiazole ligands was performed to generate starting complexes for conformational ensemble generation. Re-docking of the crystallographic ligands into the prepared PI3Kα, PI3Kβ, PI3Kγ, and PI3Kδ structures yielded heavy-atom RMSD values below 2 Å (0.518 Å, 1.049 Å, 0.857 Å, and 0.258 Å for PI3Kα, PI3Kβ, PI3Kγ, and PI3Kδ, respectively), confirming that the docking setup was able to reproduce the reference binding modes.

MD simulations were used as a local conformational refinement step to generate representative receptor conformations for ensemble docking, rather than as an exhaustive analysis of long-timescale protein dynamics. Inspection of the trajectories indicated preservation of the binding site architecture during the analyzed production phase. Representative structures were therefore extracted from the locally stabilized part of each trajectory by active site RMSD-based clustering, and the five most populated cluster centroids were selected for each isoform–ligand complex. The resulting centroid structures were used as receptor ensembles (five structures per ensemble—for each PI3K isoform) for subsequent docking and 3D-QSAR modeling (Table S1).

The initial docking poses showed a broadly conserved binding orientation of **B1** and **T1** across the four PI3K isoforms, as illustrated in Figures S2 and S3, while the MD-derived centroids refined these poses into interaction patterns that more closely reflected the experimentally established hinge- and selectivity-related contacts of PI3K inhibitors. Following local relaxation of **B1** complexes, some contacts were strengthened or re-established through alternative ligand atoms. In PI3Kα, stabilization was dominated by hydrogen bonding with αVal851 through the benzoxazepine oxygen or urea nitrogen, reinforced by interactions with αSer854. The amide group also interacted with αGln859, most prominently in centroid αBzo2, which displayed the richest interaction profile through strong hydrophobic anchoring and additional polar contacts with αAsp810 and αAsp933. For **T1**, centroid αThz5 represented the most stabilized PI3Kα conformation, combining hinge hydrogen bonding with αVal851 and extensive hydrophobic interactions in the hinge and affinity pocket regions. In the selected PI3Kβ, PI3Kγ, and PI3Kδ centroids, the corresponding hinge region and P-loop interactions were generally less persistent than in PI3Kα.

The ensemble docking procedure was also validated by re-docking the reference ligands in cluster centroids obtained from the MD simulations. All RMSD values are below the threshold as well and are presented in Table 1. The slightly higher re-docking RMSD obtained for PI3Kβ may reflect minor local geometric uncertainty associated with the homology-modeled human PI3Kβ structure; however, the value remained below 2 Å and indicated satisfactory reproduction of the reference binding orientation.

### 2.2. 3D-QSAR Study Validation and Defined Applicability Domains

The predicted bioactive conformations of the dataset, obtained from the molecular docking into multiple representative conformations of PI3Kα, PI3Kβ, PI3Kγ, and PI3Kδ, were used to calculate GRIND-2 descriptors and build four PLS models for each isoform. The predicted activities for both the training and test sets are shown in Table S3, and the plots of observed versus predicted activities are shown in Figure S9.

We performed extensive statistical validation to ensure the reliability of our built models. The parameters of internal and external validation, including r-metrics, are presented in Table 2.

The PI3Kα, PI3Kβ, PI3Kγ, and PI3Kδ models were analyzed to determine their applicability domain using ten variables with the highest PLS coefficients. The leverage value limit was calculated using Equation (9) and found to be h* = 1.320 for the PI3Kα, PI3Kγ, and PI3Kδ models and h* = 1.375 for PI3Kβ. The Williams plots shown in Figure S10 indicate that both the training and test sets for all models are within the defined ADs.

3D-QSAR and molecular docking results analysis:

GRIND descriptors are computed based on the interaction energies between the molecules and MIFs. We interpreted and visualized the selected GRIND descriptors, which contribute to the QSAR models, to understand their influence on the inhibitory activity of each PI3K isoform. They provide the numerical values of the product of the fields and the distance between them for the most important pairs of nodes that contribute negatively or positively to the inhibitory activity. The selection of the interpreted variables was made based on their PLS coefficients. The PLS coefficient plots are displayed for each model, in which the selected variables are indicated. All variables obtained from each model are graphically represented in diagrams called “correlogram plots”, wherein the products of these interaction energies are plotted versus the distance between them. Four auto-correlograms, including DRY–−DRY, O–O, N1–N1, and TIP–TIP, belonging to the same MIF, and six cross-correlograms (DRY–O, DRY–N1, DRY–TIP, O–N1, O–TIP, and N1−TIP) from different MIFs were obtained using interaction energy values.

The study evaluated 3D-QSAR models to determine the key structural features that affect inhibitory activity in each PI3K isoform (α, β, γ, and δ) with a primary focus on the PI3Kα model.

#### 2.2.1. PI3Kα 3D-QSAR Model

**Figure 3:**
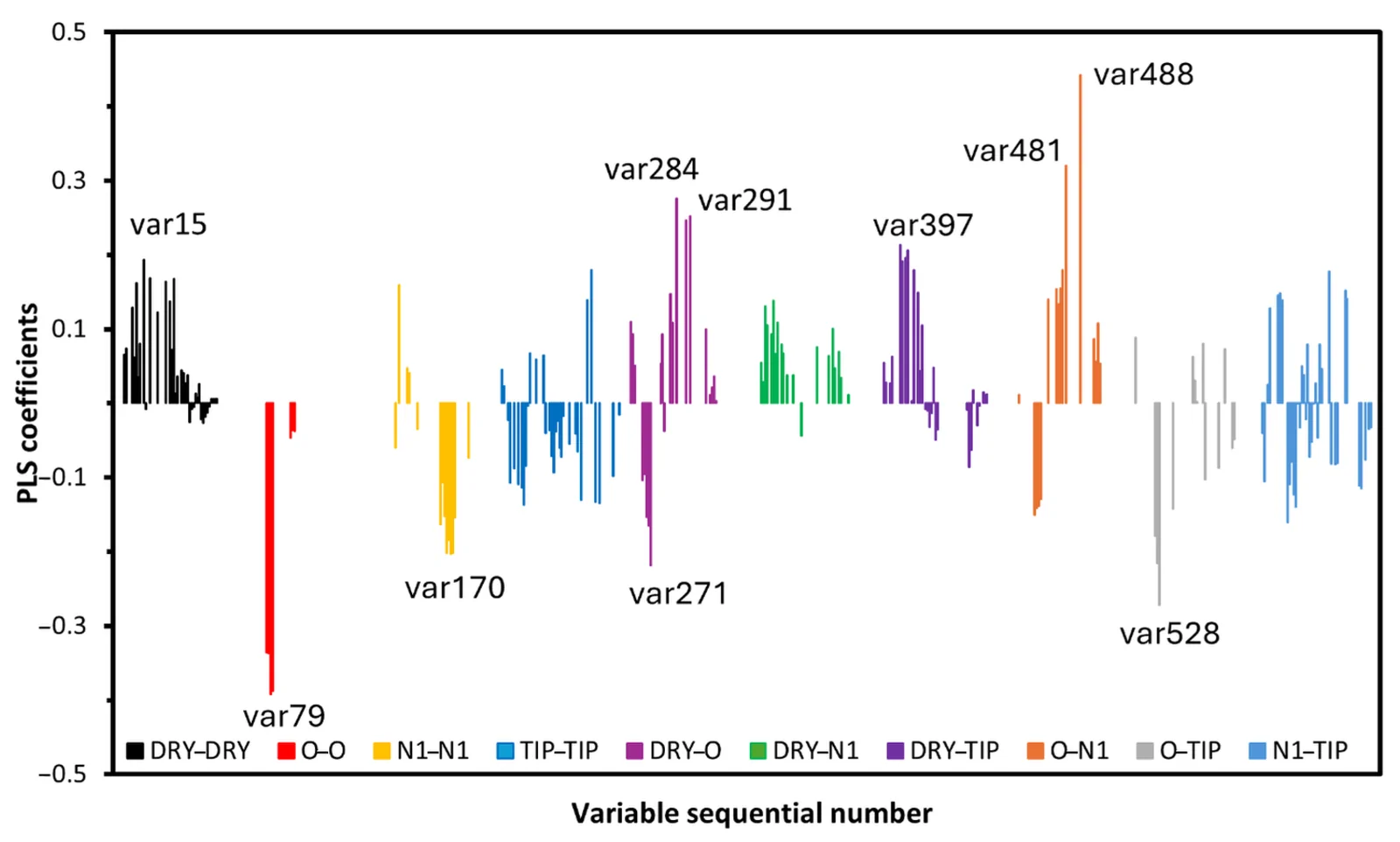
PLS coefficient plot with the most important variables indicated for the PI3Kα 3D-QSAR model.

The PI3Kα model includes 205 variables, with a model dimensionality of two latent variables. The most important variables are shown in Figure 3 and Table S4.

Almost all variables in the DRY–DRY correlogram have a positive impact on PI3Kα inhibitory activity. The one with the highest PLS coefficient is variable var15 (6.0–6.4 Å), formed between MIFs forming on both sides of a hydrophobic moiety. In Bzo cluster compounds, var15 is formed around the 1,2,4-triazole ring. Thz cluster compounds have this variable formed around the benzene ring connected to the thiazole ring. All compounds have var15 expressed, with the Bzo cluster compounds having a higher value of above 0.6.

Variable var79 (6.0–6.4 Å), from the O–O correlogram, has the highest negative impact on the activity and depicts the distances between MIFs interacting with two hydrogen bond donors. It is present only in less active thiazole derivatives and is formed between the urea hydrogen and the amide group hydrogen.

**Figure 4:**
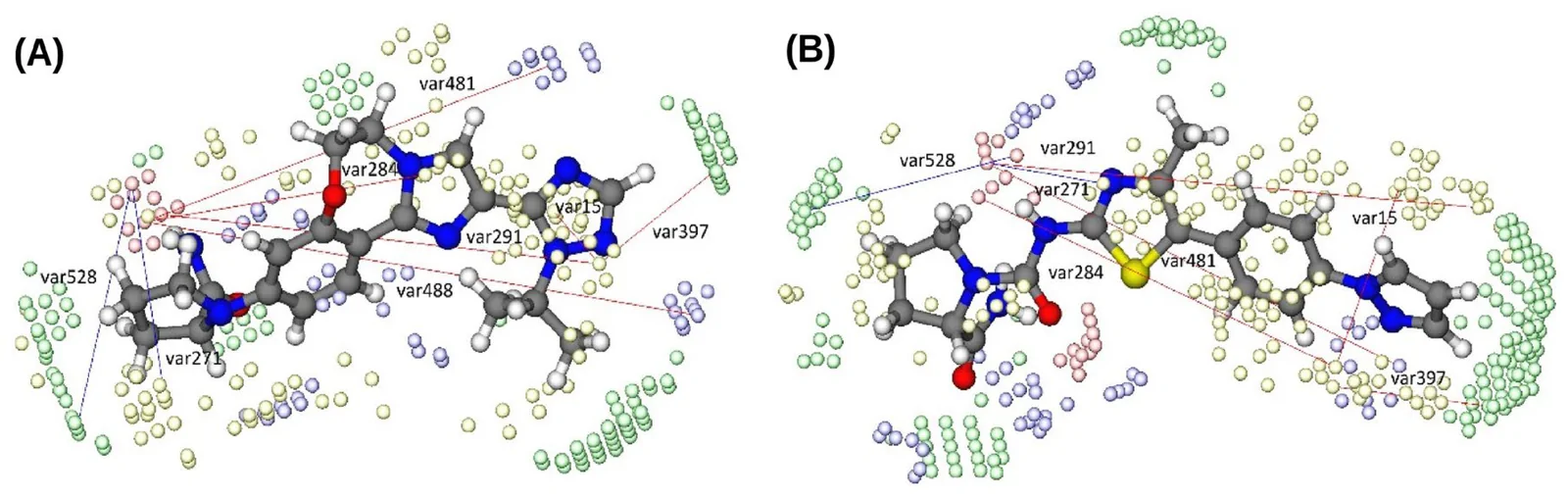
The most important positive (red lines) and negative (blue lines) variables from the PI3Kα 3D-QSAR model for compounds: (**A**) **B1** and (**B**) **T1**; blue spheres represent N1 probes (hydrogen bond donors), red spheres represent O probes (hydrogen bond acceptors), yellow spheres represent DRY probes (hydrophobic centers), and green spheres represent TIP probes (steric hot spots).

Variable var170 (16.8–17.2 Å) from the N1–N1 group is formed between two MIFs interacting with two hydrogen bond acceptors. It is formed in some compounds from both clusters. Benzoxazepine derivatives exhibit this variable between the triazole nitrogen and amide oxygen atoms, whereas thiazole derivatives display it between the sulfone oxygen or imidazole ring oxygen and the amide oxygen. For example, compounds **B1** and **B19**, the most active and least active Bzo cluster compounds, and **T1** and **T18**, the most active and least active Thz cluster compounds, are missing this variable (Figure 4).

The DRY–O correlogram shows three important variables: var271 (6.0–6.4 Å), var284 (11.2–11.6 Å) and var291 (14.0–14.4 Å). The variables depict distances between different hydrophobic entities and the amide group hydrogen, acting as a hydrogen bond donor. In Bzo cluster compounds, var271 depicts a nonoptimal distance of the hydrogen from the adjacent hydrophobic moiety in the R_1_ substituent. On the other hand, variables var284 and var291 depict the positive impact of the distance between the same hydrogen and two other hydrophobic moieties: the joined imidazole ring and the 1,2,4-triazole, respectively. In Thz cluster compounds, var271 connects an amide or urea hydrogen with the thiazole ring, and var284 and var291 connect the same hydrogen with the benzene ring.

The DRY-TIP correlogram shows var397 (5.2–5.6 Å) as an important variable with a positive coefficient. The existence of a steric moiety and an adjacent hydrophobic moiety positively affect the PI3Kα inhibitory activity. Benzoxazepine derivatives have a 1,2,4-triazole ring and a hydrogen or methyl group in position 3 of the ring that interact with the described MIFs. Thiazole derivatives express the variable between the thiazole ring and the adjacent methyl substituent, with a few exceptions. The latter group exhibits lower values of this variable.

Variables var481 (13.2–13.6 Å) and var488 (16.0–16.4 Å) from the O–N1 correlogram positively affect the inhibitory activity and are formed between a hydrogen bond donor and a hydrogen bond acceptor. Variable var488 has the highest positive impact on the inhibitory activity and is expressed only in Bzo cluster compounds. Both depict the existence of an amide group hydrogen and nitrogen in the 1,2,4-triazole. Variable var481 connects the hydrogen in the amide or urea group and the pyrazole ring nitrogen or sulfone group oxygen.

The O-TIP correlogram contains var528 (6.4–6.8 Å), which has a significant negative impact on the inhibitory activity. The variable is formed in both clusters between the amide group hydrogen, which acts as a hydrogen bond donor, and a steric entity in close proximity. This variable is not expressed in compound **T18**.

#### 2.2.2. Molecular Docking Study of PI3Kα Isoform

The obtained poses from the performed docking procedure were analyzed to further explore possible interactions between PI3Kα inhibitors and the enzyme. All Bzo cluster compounds except **B9** form a hydrogen bond with αVal851, the hinge region amino acid to which most ATP-competitive PI3K inhibitors bind [12]. The oxazepine oxygen acts as a hydrogen bond acceptor in the interaction. Other interactions that are formed by the benzoxazepine ring are hydrophobic interactions with αGlu849, αTyr836, αMet922, αIle932, and αVal850 from the hinge region. The benzene ring and the substituent extend to the hydrophobic region II, forming different interactions based on the substituent. Some compounds with the amide group form hydrogen bonds with αArg852, αSer854, and αGln859. The imidazole and the adjacent 1,2,4-triazole ring bind to the affinity pocket by making hydrophobic contacts with the residues αIle932 and αIle848. The isopropyl group, which is contained in almost all derivatives of this cluster, expands further into the pocket, binding with amino acids, like αIle800 and αTyr836, and p-loop residues, like αMet772 and αPro778. Different side chains connected to the benzene/pyridine ring show diverse interactions. The most active compound, **B1**, which contains a primary amide group, forms hydrogen bonds with αGln859, a non-conserved amino acid. Another hydrogen bond is also formed with αSer854. The interaction with serine is conserved across the Bzo cluster. Shorter side chains containing the amide group tend to form hydrogen bonds with αArg852 or αAsn853. The cluster compounds form two hydrogen bonds with αVal851 via the thiazole ring nitrogen, which acts as a hydrogen bond acceptor, and the adjacent nitrogen from the urea or amide group, acting as a hydrogen bond donor. Sulfonamide derivatives also form a hydrogen bond with αLys802. The benzene ring forms different hydrophobic contacts with amino acids in the affinity pocket, such as αTyr836 and αIle932, and the adjacent moiety (imidazole, sulfonamide, etc.) extends further, interacting with the affinity pocket and the p-loop residues, targeting αIle848, αGlu849, αTrp780, αAsp933, and αPhe930, among others. Some compounds, like **T1**, containing an (*S*)-pyrrolidine carboxamide moiety, also form a hydrogen bond with Ser854 and hydrophobic interactions with αHis855 and αGln859 from the hydrophobic region II.

The 3D-QSAR model for PI3Kα isoform identified key variables such as var15 (DRY–DRY), var481 (O–N1), and var488 (O–N1), highlighting the importance of hydrophobic contacts and H-bond interactions for inhibitory activity. The distance captured by var15 (6.0–6.4 Å) reflects the same spatial separation observed between αTyr836 and αIle932 in the affinity pocket. In turn, var481 (13.2–13.6 Å) and var488 (16.0–16.4 Å) parallel the distances connecting αTyr836/αIle932 with hinge residues αSer854 and αGln859, respectively. Another important variable, var284 (DRY–O: 11.2–11.6 Å), further supports the importance of the described hydrogen bonds while also emphasizing the importance of the hydrophobic properties of the hinge binder itself—corresponding to the distance between a hydrogen bond donor—αGln859—and a hydrophobic contact with αVal850. Therefore, effective PI3Kα inhibitors should possess strong hydrophobic features at hinge and affinity pocket binders and include hydrogen bond acceptors capable of interacting primarily with αVal851, αSer854, and αGln859.

#### 2.2.3. PI3Kβ 3D-QSAR Model

**Figure 5:**
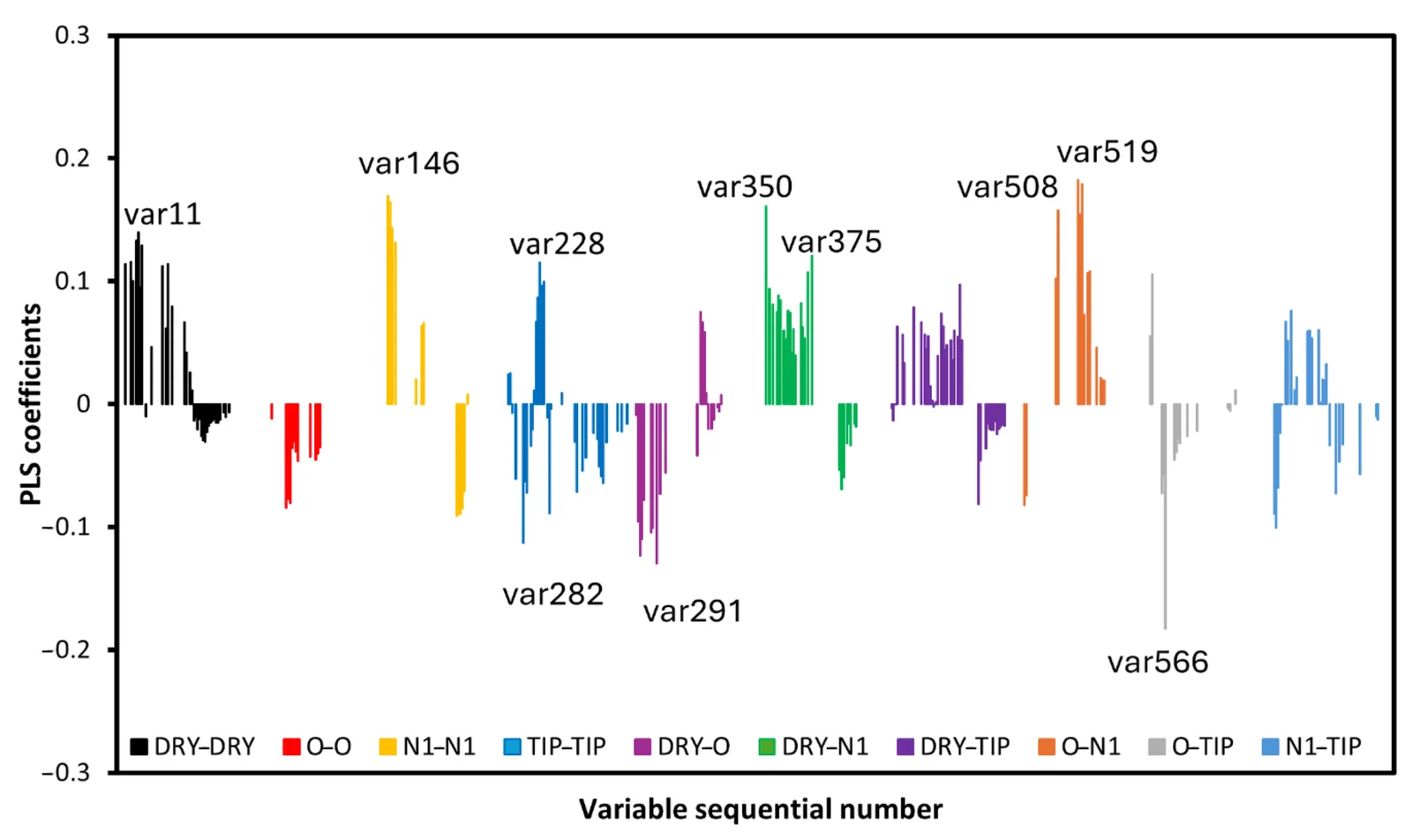
PLS coefficient plot with the most important variables indicated for the PI3Kβ 3D-QSAR model.

In the PI3Kβ model, there are 231 variables, and the model dimensionality is defined by two latent variables. Figure 5 and Table S5 illustrate the key variables of significance.

In the O–O correlogram, most variables positively correlate with the PI3Kβ inhibitory activity. One of the most impactful variables is var11 (4.4–4.8 Å). It describes the importance of the pair of two hydrophobic centers (imidazole and triazole rings in the Bzo cluster, and thiazole and benzene in the Thz cluster). The variable is expressed in all compounds, and in all compounds with p*K*_i_ higher than 7.0, its value is higher than 0.5.

A N1–N1 variable—var 146 (3.2–3.6 Å) —has a significant impact on activity, with a positive PLS coefficient. It depicts the benzoxazepine oxygen, the adjacent imidazole nitrogen, and two carbonyl oxygens from amide and urea groups at an optimal distance. It is not expressed in some Thz cluster compounds with lower activity.

Variable var228 (8.4–8.8 Å) from the TIP–TIP correlogram indicates that the presence of two steric moieties at an optimal distance positively affects activity. The variable is expressed between the triazole ring and isopropyl group in benzoxazepine derivatives and between different entities in thiazole derivatives.

**Figure 6:**
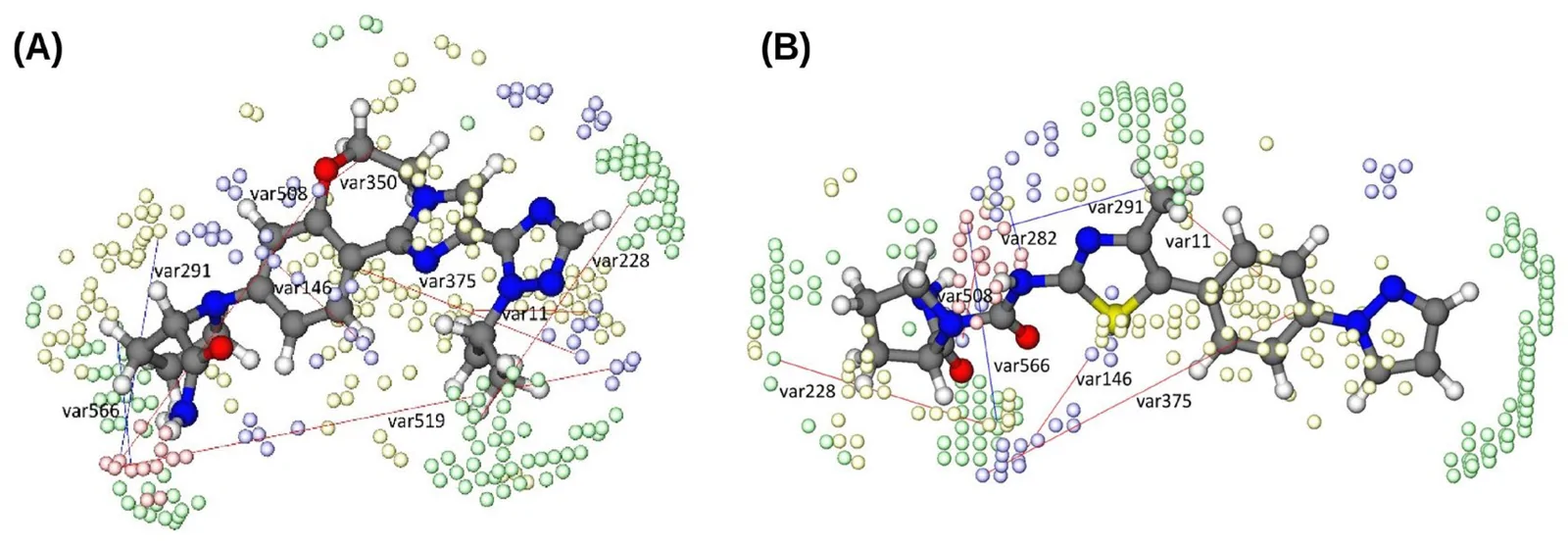
The most important positive (red lines) and negative (blue lines) variables from the PI3Kβ 3D-QSAR model for compounds (**A**) **B1** and (**B**) **T1**; blue spheres represent N1 probes (hydrogen bond donors), red spheres represent O probes (hydrogen bond acceptors), yellow spheres represent DRY probes (hydrophobic centers), and green spheres represent TIP probes (steric hot spots).

Most DRY–O variables have a negative impact on the activity. The distance between MIFs interacting with a hydrogen bond donor, like an amide hydrogen, and a hydrophobic moiety, either a hydrophobic substituent or an aromatic ring, is described with two variables: var282 (2.4–2.8 Å) and var291 (6.0–6.4 Å). Variable var282 is not present in more active Bzo cluster compounds, including **B1** (Figure 6A).

Variables var350 (2.0–2.4 Å) and var375 (12.0–12.4 Å) in the DRY-N1 correlogram are formed between a hydrogen bond acceptor and a hydrophobic moiety. Variable var350 is formed in almost all Bzo cluster compounds and in a few Thz cluster compounds. **B19**, Compound **T1** does not form this variable (Figure 6B). In benzoxazepine derivatives, it represents the distance between MIFs that interact with the nitrogen of the imidazole ring (var350) or triazole ring (var375) as a hydrogen bond donor and the hydrophobic oxazepine ring. The carbonyl oxygen of the amide group/sulfone oxygen is at an optimal distance for the PI3Kβ inhibitory activity from the benzene/ring in Thz cluster compounds.

Amide groups form two variables from the O–N1 group. Variable var508 (10.0–10.4 Å) is formed between the amide hydrogen as a hydrogen bond donor and the imidazole/thiazole ring nitrogen as a hydrogen bond acceptor, and var519 (14.4–14.8 Å) is formed between the amide hydrogen as a hydrogen bond donor and the triazole ring/urea nitrogen as a hydrogen bond acceptor.

Var566 (5.6–6 Å) belongs to the O–TIP variables and describes the distance between the amide hydrogen and the adjacent steric entity. Compound **T1** does not have these two variables expressed.

#### 2.2.4. Molecular Docking Study of PI3Kβ Isoform

The docking studies indicate that, unlike the α isoform, Bzo cluster derivatives do not typically form a hydrogen bond with the hinge region, specifically βVal854. Only a few compounds with higher PI3Kβ inhibitory activity form an interaction with it. Other interactions include hydrogen bonds with βSer799 and βThr856. Less active compounds tend to form hydrogen bonds with βTrp787. Hydrophobic interactions typically involve the oxazepine ring with amino acids βGlu852, βVal853, βMet926, and βPhe934 in the hinge region, as well as interactions via the triazole ring and the isopropyl substituent with βMet779, βPro785, βIle803, βLys805, βIle851, and βIle936.

In contrast, Thz cluster compounds tend to form a hydrogen bond with βVal854, and some compounds also interact with βSer857 and βGlu858. However, compared to Bzo cluster compounds, Thz cluster compounds lack many hydrophobic interactions. The interactions are primarily with the hinge region via the thiazole ring with βTyr839, βGlu852, βVal853, and βPhe0934.

The PI3Kβ 3D-QSAR model highlighted the presence of var11 (DRY–DRY) and var228 (TIP–TIP) variables, emphasizing favorable hydrophobic and steric interactions. The distance of var11 (4.4–4.8 Å) parallels the separation between interactions of imidazole with βVal853 and triazole rings with βPhe934, while var228 (8.4–8.8 Å) corresponds to the spacing between steric moieties such as the triazole ring and isopropyl group contacting βIle803 and βIle936, respectively. Positive O–N1 variables, var519 (14.4–14.8 Å) and var508 (10.0–10.4 Å), capture donor–acceptor distances that match the hydrogen bond geometries of amide groups interacting with hinge residues βVal854, βSer799, and βThr856. In contrast, negative var291 (DRY–O: 6.0–6.4 Å) and var 566 (O–TIP: 5.6–6 Å) variables describe unfavorable distances between a hydrogen bond donor group and steric and hydrophobic regions, reflecting the lack of a hydrogen bond in the amide group and suboptimal binding of the triazole ring in the activity pocket. Taken together, effective PI3Kβ inhibition is characterized by strong hydrophobic and steric interactions within the hinge region and affinity pocket, complemented by hydrogen bonds with βVal854, βSer799, and βThr856, while avoiding steric clashes and poorly oriented hydrogen bond donors.

#### 2.2.5. PI3Kγ 3D-QSAR Model

**Figure 7:**
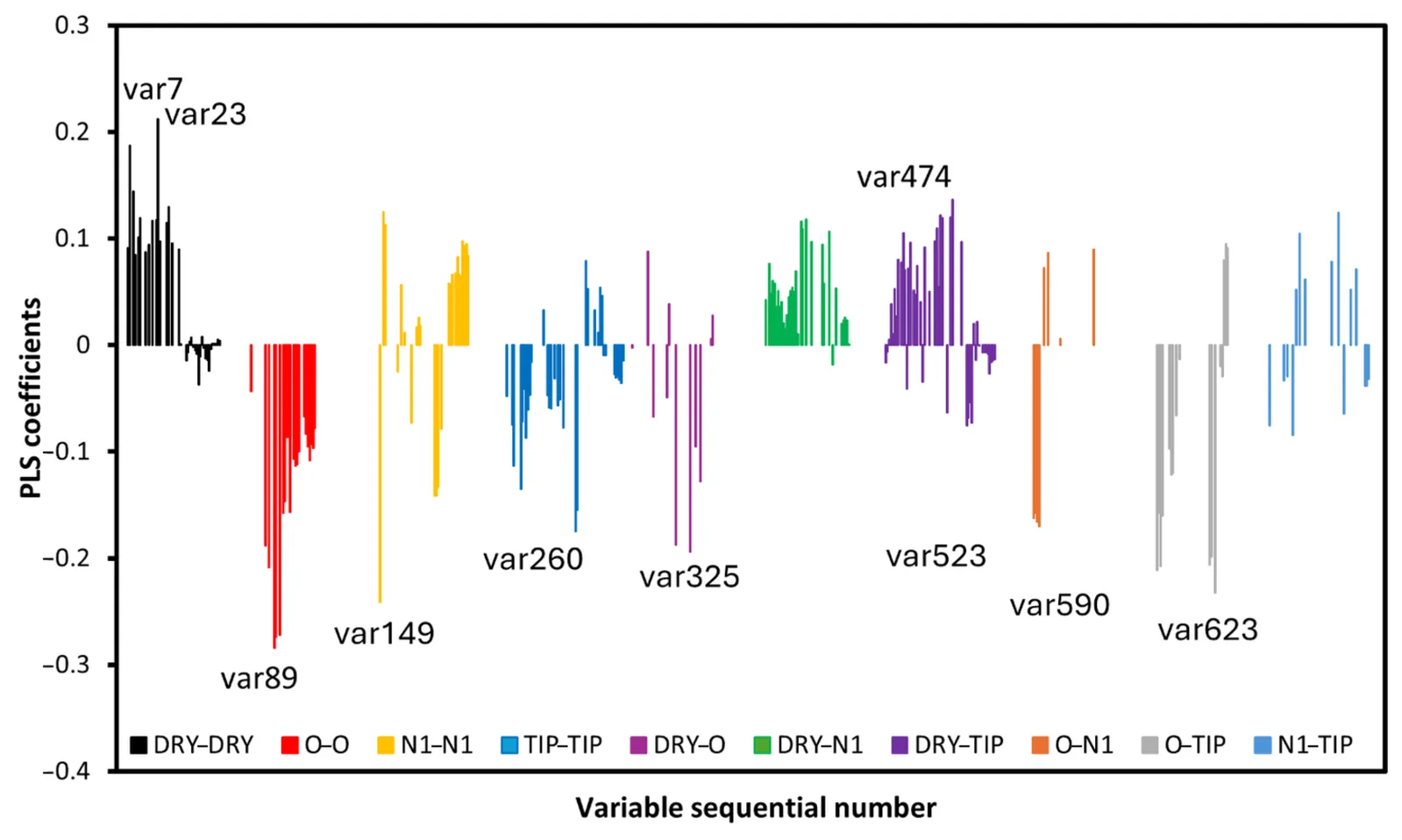
PLS coefficient plot with the most important variables indicated for the PI3Kγ 3D-QSAR model.

The PI3Kγ model consists of 247 variables, and two latent variables determine its dimensionality. Figure 7 and Table S6 highlight the most critical variables.

**Figure 8:**
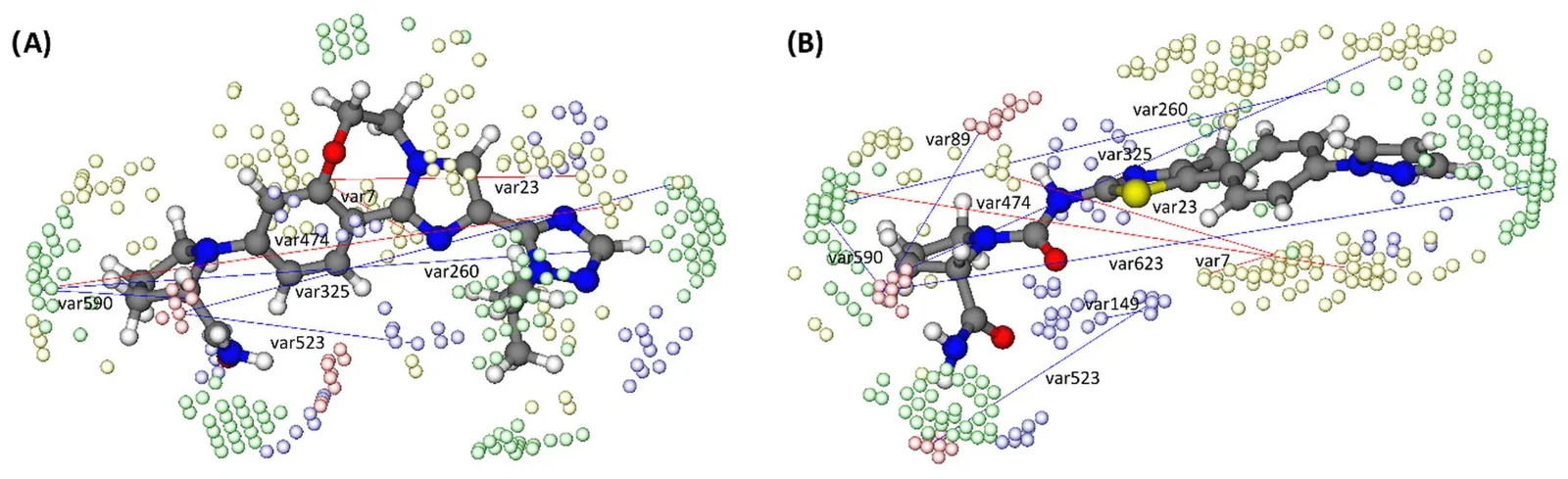
The most important positive (red lines) and negative (blue lines) variables from the PI3Kγ 3D-QSAR model for compounds (**A**) **B1** and (**B**) **T1**; blue spheres represent N1 probes (hydrogen bond donors), red spheres represent O probes (hydrogen bond acceptors), yellow spheres represent DRY probes (hydrophobic centers), and green spheres represent TIP probes (steric hot spots).

Most DRY–DRY variables have a positive influence on PI3Kγ inhibitory activity. Variables var7 (2.8–3.2 Å) and var23 (9.2–9.6 Å) are the two most influential positive variables, describing a distance between two hydrophobic entities. Variable var7 depicts the distance between imidazole and triazole in benzoxazepine derivatives, or between the thiazole and benzene ring in thiazole derivatives. Variable var23 usually describes the distance between the pyridine or benzene ring fused as one with the oxazepane ring and the triazole ring in the Bzo cluster as another entity, or between the thiazole ring and different entities in the Thz cluster. The most active compound, **B1** (Figure 8A), has a high value of this descriptor and is formed between the benzene and thiazole rings.

All O–O variables negatively impact the activity. The most important variable in this correlogram, var89 (6.8–7.2 Å), is expressed only in a few less active Thz cluster compounds. It depicts the distance between the urea hydrogen and amide hydrogen as two hydrogen bond donors.

Var149 (2.0–2.4 Å), an N1–N1 variable, is formed in several compounds of the dataset, describing a nonoptimal distance between two hydrogen bond acceptors—oxazepine oxygen and imidazole nitrogen—in Bzo cluster compounds and thiazole nitrogen and carbonyl oxygen in Thz cluster compounds. This variable is not present in compound **B1** but is present in **T1** (Figure 8B).

One TIP–TIP variable has a significant negative impact on the inhibitory activity. Variable var260 (17.6–18.0 Å) is formed between the terminal parts of the R_1_ and R_2_ substituents in Bzo cluster compounds and the R_1_ and R_3_ substituents in Thz cluster compounds, depicting the steric shape of the compound itself. The variable shows the negative impact of compound size on the PI3Kγ inhibitory activity.

Variable var325 (14.8–15.2 Å) in the DRY–O correlogram also negatively affects the activity. The variable is expressed between a hydrophobic moiety in the R_1_ substituents in both clusters and the amide or urea hydrogen, acting as a hydrogen bond donor.

The DRY–TIP correlogram shows var474 (16.8–17.2 Å), which is expressed similarly to var260, but with the R_1_ substituent as a hydrophobic entity and a positive impact on the activity.

Variable var523 (7.6–8 Å) in the O–N1 correlogram represents the distance between a hydrogen bond donor and a hydrogen bond acceptor. Only a few of the most active compounds do not form it. Bzo cluster compounds form the variable between the diazole’s nitrogen and the amide group’s hydrogen. Thz cluster compounds express the variable between the urea carbonyl oxygen and either the urea group hydrogen atom or amide group hydrogen atom.

The O–TIP correlogram includes two variables depicting the distance between a hydrogen bond donor and a hydrophobic entity—var590 (5.6–6.0 Å) and var623 (18.8–19.2 Å). Variable var590 is formed between the amide group hydrogen and different steric moieties in close vicinity, and it is not formed in the most PI3Kγ active compound **B13**. Variable var623 is more dominantly expressed in less active compounds. In benzoxazepine derivatives, it is formed between the amide group hydrogen and the R_3_ substituent—either an isopropyl group at position 1 or a hydrogen/methyl group at position 3 of the 1,2,4-triazole. Thiazole derivatives express the variable between the amide group hydrogen and different steric entities in the R_3_ substituent.

#### 2.2.6. Molecular Docking Study of PI3Kγ Isoform

Bzo cluster compounds also form a hinge hydrogen bond with γVal882. The triazole nitrogen in some compounds also forms a hydrogen bond with γLys833 in the affinity pocket. Other hydrogen bonds can be formed with γAsp884 and γAla885 from hydrophobic region II. Hydrophobic contacts are made with different amino acids, including γTyr879, Ile879, and γIle963 in the hinge region; γMet804, γPro810, γLeu838, and γIle879 in the affinity pocket; and γMet953, as well.

Some more active Thz cluster compounds also form a hydrogen bond with γVal882 via the thiazole nitrogen, whereas less active ones lack the interaction. Sulfone derivatives also form a hydrogen bond with γAsp964. Different hydrophobic contacts can be noticed, including those with γGlu880, γIle963, Ile963, and γMet953 in the hinge region; γLeu845, γTyr867, γIle879, and γIle963 in the affinity pocket; and γMet804 and γSer806.

The 3D-QSAR model for the PI3Kγ isoform emphasized hydrophobic spacing and steric balance described by var11 and var23 (DRY–DRY) and var260 (TIP–TIP), respectively. Var11 (2.8–3.2 Å) reflects short-range contacts between γTyr879 and γIle963, while var23 (9.2–9.6 Å) parallels the distance between two hydrophobic interactions involving γIle879 and γVal882. Var260 (17.6–18.0 Å) corresponds to the steric spacing between groups contacting γLys833 in the affinity pocket and γMet953. In contrast, negative variables, such as var623 (O–TIP), describe an unfavorable distance between a hydrogen bond donor interaction from the selectivity-modifying moiety and a steric interaction with the affinity pocket binder, consistent with weaker binding poses and a lack of formed hydrogen bonds. Both analyses show that the potency of PI3Kγ inhibitors depends on balanced hydrophobic fitting and steric complementarity in the hinge region and the affinity pocket, while misaligned polar groups in the selectivity modifiers weaken the binding.

#### 2.2.7. PI3Kδ 3D-QSAR Model

**Figure 9:**
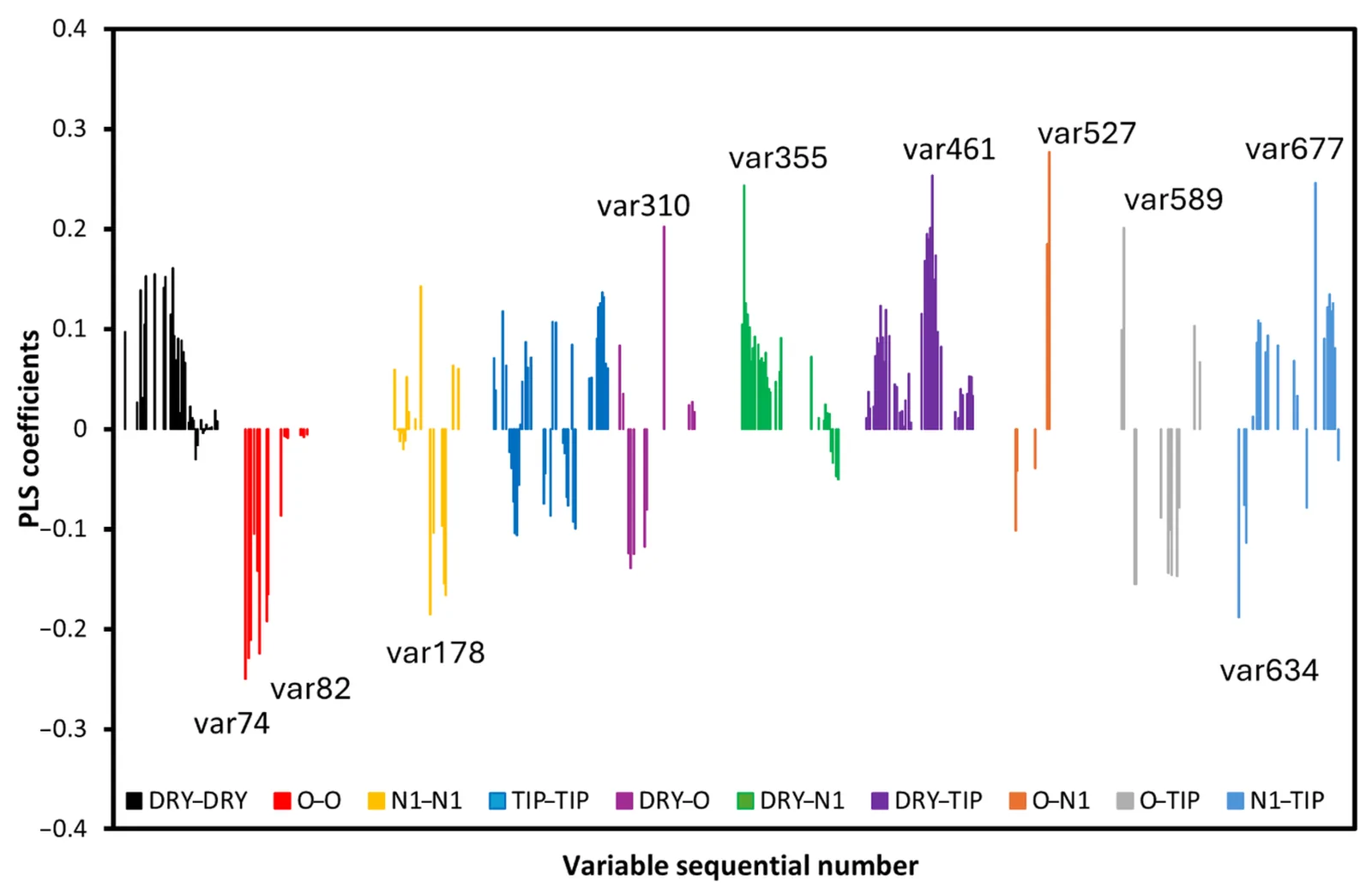
PLS coefficient plot with the most important variables indicated for the PI3Kδ 3D-QSAR model.

With 205 variables, the PI3Kδ model’s dimensionality is characterized by two latent variables, as depicted in Figure 9 and Table S7, which show the crucial variables.

All O-O variables have a negative influence on PI3Kδ inhibitory activity. Two variables have a significant impact on the activity—var74 (1.6–2.0 Å) and var82 (4.8–5.2 Å), depicting the distance between two hydrogen bond donors. It is expressed only in a few Thz cluster compounds. Variable var74 is formed between two urea hydrogens, and var82 is formed between the urea hydrogen and amide group hydrogen.

**Figure 10:**
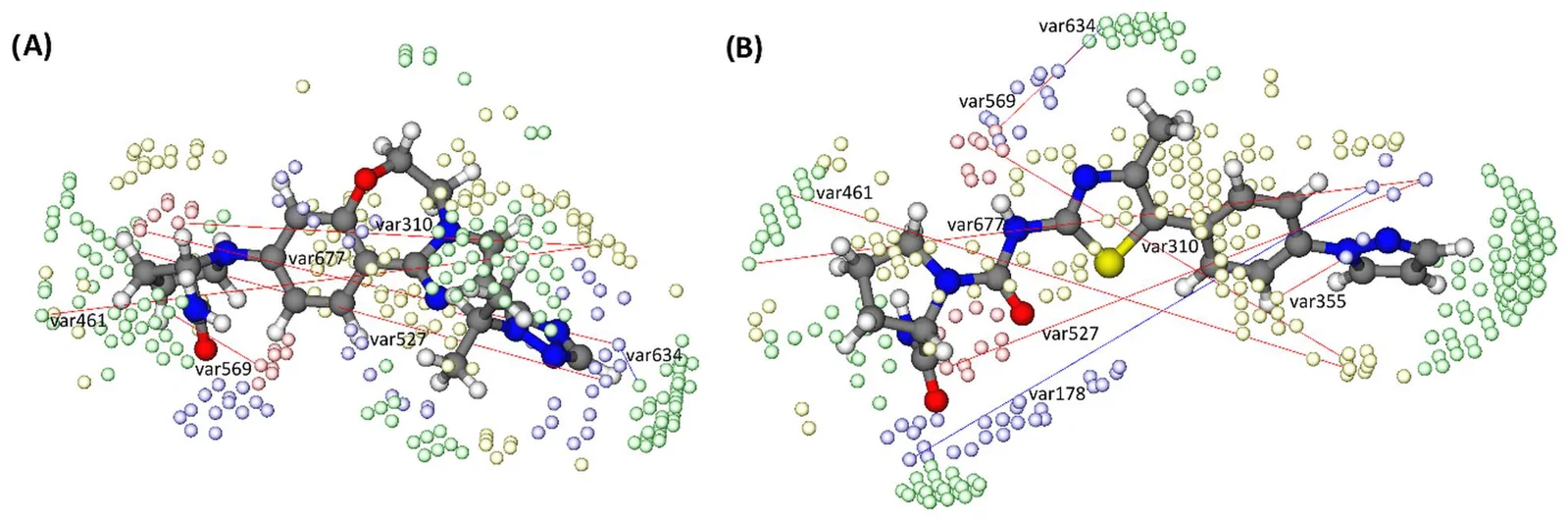
The most important positive (red lines) and negative (blue lines) variables from the PI3Kδ 3D-QSAR model for compounds (**A**) **B1** and (**B**) **T1**; blue spheres represent N1 probes (hydrogen bond donors), red spheres represent O probes (hydrogen bond acceptors), yellow spheres represent DRY probes (hydrophobic centers), and green spheres represent TIP probes (steric hot spots).

Variable var178 (15.2–15.6 Å) from the N1-N1 correlogram has a negative impact on the activity. It is formed between the 1,2,4-triazole nitrogen and different hydrogen bond acceptors in the R_1_ substituent in Bzo cluster compounds and between the amide/urea oxygen and different hydrogen bond acceptors in the R_3_ substituent in Thz cluster compounds. This is one of two negative variables formed in **T1** and is not present in **B1** (Figure 10).

Variable var310 (12.0–12.4 Å) belongs to the DRY–O type and describes the distance between a hydrogen bond donor and a hydrophobic moiety. It positively impacts the activity and is formed between the amide hydrogen and 1,2,4-triazole in benzoxazepine derivatives or between the amide/urea oxygen and the benzene ring in thiazole derivatives.

DRY–N1 shows var355 (2.0–2.4 Å), depicting the optimal distance between two MIFs formed by an imidazole or 1,2,4-triazole ring as a hydrophobic moiety and its nitrogen as a hydrogen bond acceptor in Bzo cluster compounds. Thz cluster compounds form this variable between benzene and a hydrogen bond acceptor in the R_3_ substituent.

All DRY–TIP variables positively affect the activity, and var461 (16.4–16.8 Å) connects the hydrophobic 1,2,4-triazole and different R_1_ substituents as steric entities in the Bzo cluster. This variable is usually expressed between the hydrophobic benzene ring and the R_3_ substituent as a steric moiety in the Thz cluster.

Variable var527 (14.8–15.2 Å) has the highest positive PLS coefficient in this model and belongs to the O–N1 correlogram. It describes the distance between a hydrogen bond donor and an acceptor. It is mostly formed in benzoxazepine derivatives, between the amide group’s hydrogen and nitrogen from 1,2,4-triazole. Thiazole derivatives express this variable between the amide or urea hydrogen and the pyrazole nitrogen or the keto group oxygen from the R_1_ substituent.

Variable var569 (3.6–4 Å) from the O–TIP group also positively impacts the inhibitory activity. In Bzo cluster compounds, this variable is expressed between two MIFs interacting with the amide group’s hydrogen, acting as a hydrogen bond donor, and the oxygen, acting as a steric entity. In Thz cluster compounds, it is primarily expressed between the amide/urea group’s hydrogen and the methyl group at position 4 of the thiazole ring.

The N1–TIP correlogram shows two important variables—var634 (1.6–2 Å) and var677 (18.8–19.2 Å)—both depicting the distance between a hydrogen bond acceptor and a steric entity. For example, the benzoxazepine derivatives form these variables between the 1,2,4-triazole nitrogen and the methyl group/hydrogen (var634) or the amide group (var677). Variable var634 is the only negative variable formed in compound **B1**. Variable var634 is formed between the thiazole nitrogen and the methyl group substituent in Thz cluster compounds. Variable var677 is formed only in some thiazole derivatives, between different hydrogen bond acceptors in the R_3_ substituent and the distant parts of the R_1_ substituents as a steric entity. This variable is formed in **T1**.

#### 2.2.8. Molecular Docking Study of PI3Kδ Isoform

The docking results show that Bzo cluster compounds form a hydrogen bond with δVal828. The docking results show a lack of interaction with δAsn836, but other hydrogen bonds are formed with δArg830 and δSer831. Hydrophobic contacts are formed with δIle758, δIle777, δTyr813, δIle825, Glu826, δVal827, and δIle910 in the hinge region. In the affinity pocket, interactions occur with δMet752 and δIle910. The selectivity motif also forms a T-shaped π-π interaction with δTrp760.

Thz cluster compounds also form a hydrogen bond with δVal828 via the thiazole ring nitrogen, which acts as a hydrogen bond acceptor, and the adjacent nitrogen from the urea or amide group acts as a hydrogen bond donor. The carbonyl oxygen also forms a hydrogen bond with δTrp760. Sulfone derivatives also form a hydrogen bond with δAsp911. The thiazole hinge binder forms different hydrophobic contacts, including those with δTyr813, δIle825, δGlu826, δVal827, δMet900, and δPhe908. The benzene and adjacent substituents also form multiple hydrophobic interactions with δIle910 and δTyr813 in the affinity pocket.

The 3D-QSAR model for the PI3Kδ isoform identified key variables, such as var355 (DRY–N1), var310 (DRY–O), and var527 (O–N1), highlighting favorable hydrophobic and donor–acceptor interactions. The distance captured by var355 (2.0–2.4 Å) reflects the short distance between a hydrogen bond with δLys779 and the hydrophobic interaction with δIle825, while var310 (12.0–12.4 Å) parallels the hydrogen bond interaction with δArg830 and δSer831 and a hydrophobic interaction of the hinge binder with δIle910. Var527 (14.8–15.2 Å) corresponds to hinge hydrogen bonds involving δVal828 and δSer831. Two additional δ variables—var461 (DRY–TIP: 16.4–16.8 Å) and var677 (N1–TIP: 18.8–19.2 Å)—indicate that an increased steric contribution from the amide-adjacent substructure can enhance δ activity, which corresponds with this structure interacting δTrp760 and its distance to δIle825, whereas var634 (N1–TIP: 1.6–2.0 Å) flags steric arrangements of an activity pocket binder motif that is unfavorable for δ inhibition, with a lack of multiple interactions of this structure in the pocket. Altogether, PI3Kδ inhibition relies on strong hinge hydrogen bonding and tight hydrophobic contacts in the affinity pocket, with a more compact affinity pocket binding substructure, while introducing a bulkier selectivity-modifying moiety.

### 2.3. Comparative Analysis of 3D-QSAR and Docking Studies with Structural Insights and Design Guidelines for Selective PI3Kα Inhibitor Development

Further analysis of the obtained results was performed to elucidate key structural features that promote PI3Kα inhibitory activity while avoiding effects on the other three isoforms.

It is known that PI3Kα inhibitors form a hydrogen bond with αVal851. Additionally, for selective inhibition, other studies have already demonstrated that targeting the interaction with the non-conserved amino acid αGln859 is an effective strategy for achieving selective PI3Kα inhibition [12].

The relevance of these computationally identified features is additionally supported by the experimentally determined human PI3Kα complexes of the approved inhibitors alpelisib [26] and inavolisib [27]. The human PI3Kα–alpelisib co-crystal structure (PDB ID: 4JPS) demonstrates the combination of hinge region binding with interactions involving isoform-variable residues, including αGln859. Similarly, the human PI3Kα–inavolisib crystal structure (PDB ID: 8EXV) supports the combined importance of hinge engagement, αGln859-directed interactions, and favorable occupation of the affinity pocket region. These experimentally determined binding modes are consistent with the principal structural features identified by the docking, MD, and 3D-QSAR analyses and provide additional support for their use in the subsequent inhibitor design strategy.

**Figure 11:**
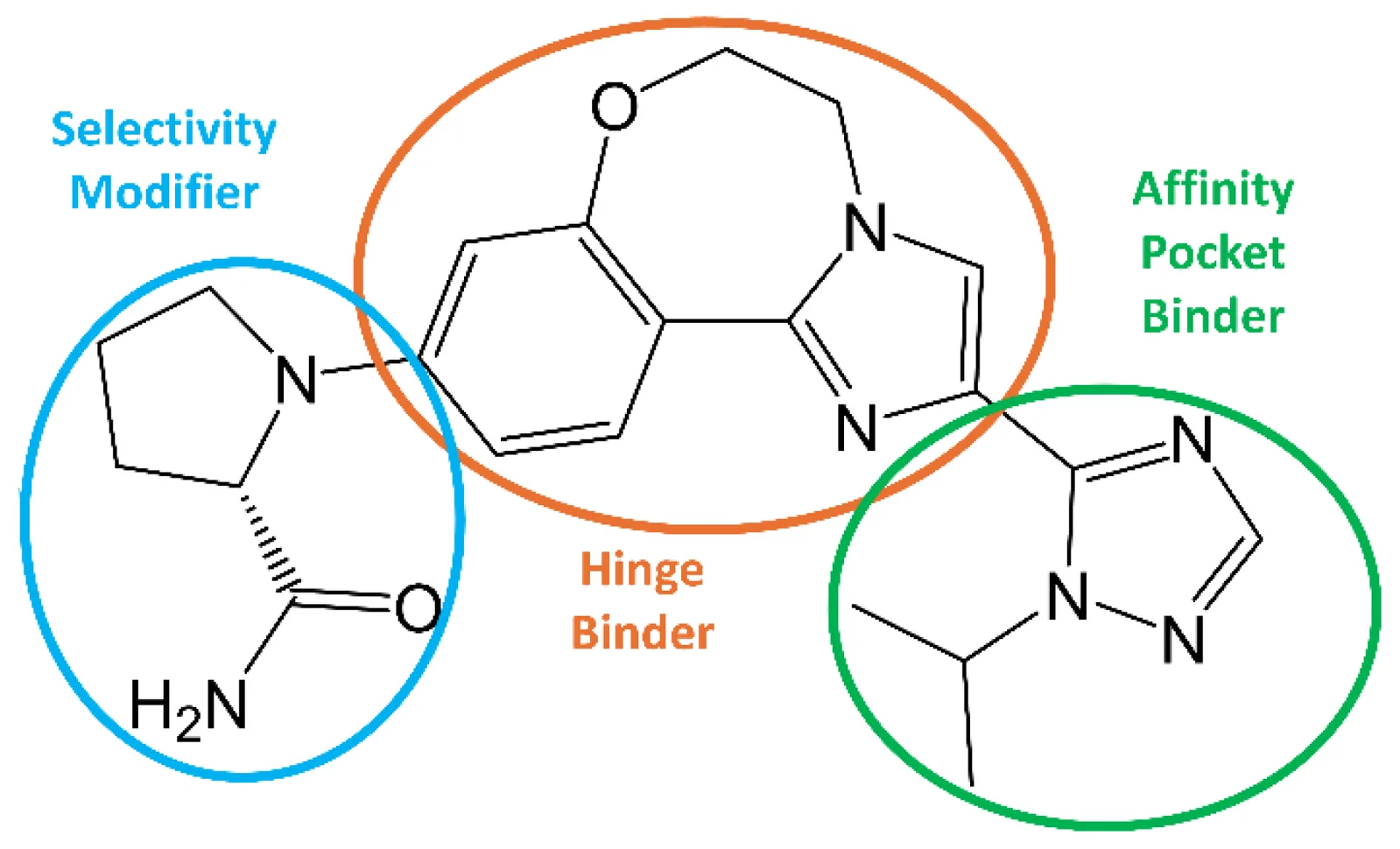
Compound **B1** and its subregions.

In this study, we aimed to utilize this approach while also exploring interactions with non-conserved amino acids, simultaneously maintaining and optimizing the positive variables of the PI3Kα model and minimizing its negative variables, and downregulating the positive variables and enhancing the negative variables of the PI3Kβ, PI3Kγ, and PI3Kδ models. For instance, var481 (O-N1: 13.2–13.6 Å) and var488 (O-N1: 16–16.4 Å) from the PI3Kα model illustrate a distance between a hydrogen bond acceptor and a hydrogen bond donor on opposite sides of a compound. The O probe interacts with the amide group, which also engages with αGln859, while the N probe interacts with a nitrogen from a triazole ring of compound **B1** (Figure 11), which binds to the affinity pocket. Thus, maintaining a hydrogen bond acceptor in the affinity pocket would facilitate the design of new, potentially selective PI3Kα inhibitors. This feature is expressed only in the PI3Kδ model, allowing for improved selectivity against the other two isoforms (var566 (O-TIP) and var291 (DRY-O) in the PI3Kβ model, and var325 (DRY-O) and var590 (O-TIP) in the PI3Kγ model).

On the other hand, the PI3Kα model presents variable var528 (O-TIP), which describes a negative effect of the steric properties of the moiety adjacent to the amide group that binds to αGln859 (pyrrolidine in **B1**) (Figure 11). The PI3Kδ model depicts three variables that indicate the positive effect of the steric properties of the selectivity-modifying substructure on PI3Kδ activity: var461 (DRY-TIP), var569 (O-TIP), and var677 (N1-TIP). Modifying this substructure and its steric properties while maintaining the ability to form a hydrogen bond with αGln859 would be another objective in the design of new compounds.

Additionally, the PI3Kα model’s variable var397 (DRY-TIP) describes a beneficial impact of the hydrophobic properties of the triazole ring and the steric properties of adjacent substituents, a characteristic that does not support PI3Kδ inhibitory activity, presented with var634 (N1-TIP). Therefore, improving the steric properties of these substituents may enhance PI3Kα activity while maintaining lower PI3Kδ activity.

Simultaneously, the designed compounds should retain the capability to form key hydrophobic interactions with specific amino acids in the affinity pocket of the PI3Kα enzyme, namely, αMet772, αPro778, αIle800, αIle848, αMet922, and others. This is supported by multiple PI3Kα model variables that describe the hydrophobicity of this substructure: var15 (DRY-DRY), var 291 (DRY-O), and var 397 (DRY-TIP).

### 2.4. Design of New PI3Kα Selective Inhibitors

The combined 3D-QSAR and ensemble-docking results defined three principal structural requirements for the design of potentially selective PI3Kα inhibitors:

1. Hinge binder: The hinge-binding scaffold should contain an appropriately positioned hydrogen bond acceptor capable of maintaining the key interaction with αVal851.
2. Affinity pocket binder: The fragment directed toward the affinity pocket should retain sufficient or enhance hydrophobicity and steric volume to establish favorable interactions within this region.
3. Selectivity modifier: A compact polar fragment should be directed toward the αGln859 interaction environment to favor PI3Kα-specific interactions while minimizing favorable accommodation in the other isoforms by reducing its bulkiness.

Based on these requirements, a scaffold hopping and R-group replacement strategy was applied to identify new hinge-binding scaffolds, affinity pocket binders, and selectivity-modifying fragments.

During the design phase of this study, a representative compound from the benzoxazepine cluster was selected as a template to guide the development of novel, potentially selective PI3Kα inhibitors. Specifically, compound **B1** (Figure 11), identified as the most active entity in the dataset and docked into the αBzo2 centroid conformation of PI3Kα (Table S1), served as the template for the scaffold hopping strategy. While both the benzoxazepine and thiazole derivatives were initially evaluated through 3D-QSAR and molecular docking, the Thz cluster representative (**T1**) was not used as a template due to its relatively lower activity (p*K*_i_ = 8.30) compared to the benzoxazepine compound (p*K*_i_ = 10.52). Furthermore, molecular dynamics simulations revealed that the thiazole derivative formed a less stable interaction with αGln859, a residue critical for PI3Kα isoform selectivity, further reducing its suitability as a design template. In contrast, the benzoxazepine derivative demonstrated more stable and favorable interactions within the binding pocket, particularly with residues known to influence isoform-specific binding, including αGln859. Geometrically favorable αGln859 hydrogen bond contacts re-formed more frequently in the **B1** complex, whereas the corresponding **T1** interaction was more sporadic. These findings support **B1** as the more appropriate template for guiding the design of selective PI3Kα inhibitors.

**Figure 12:**
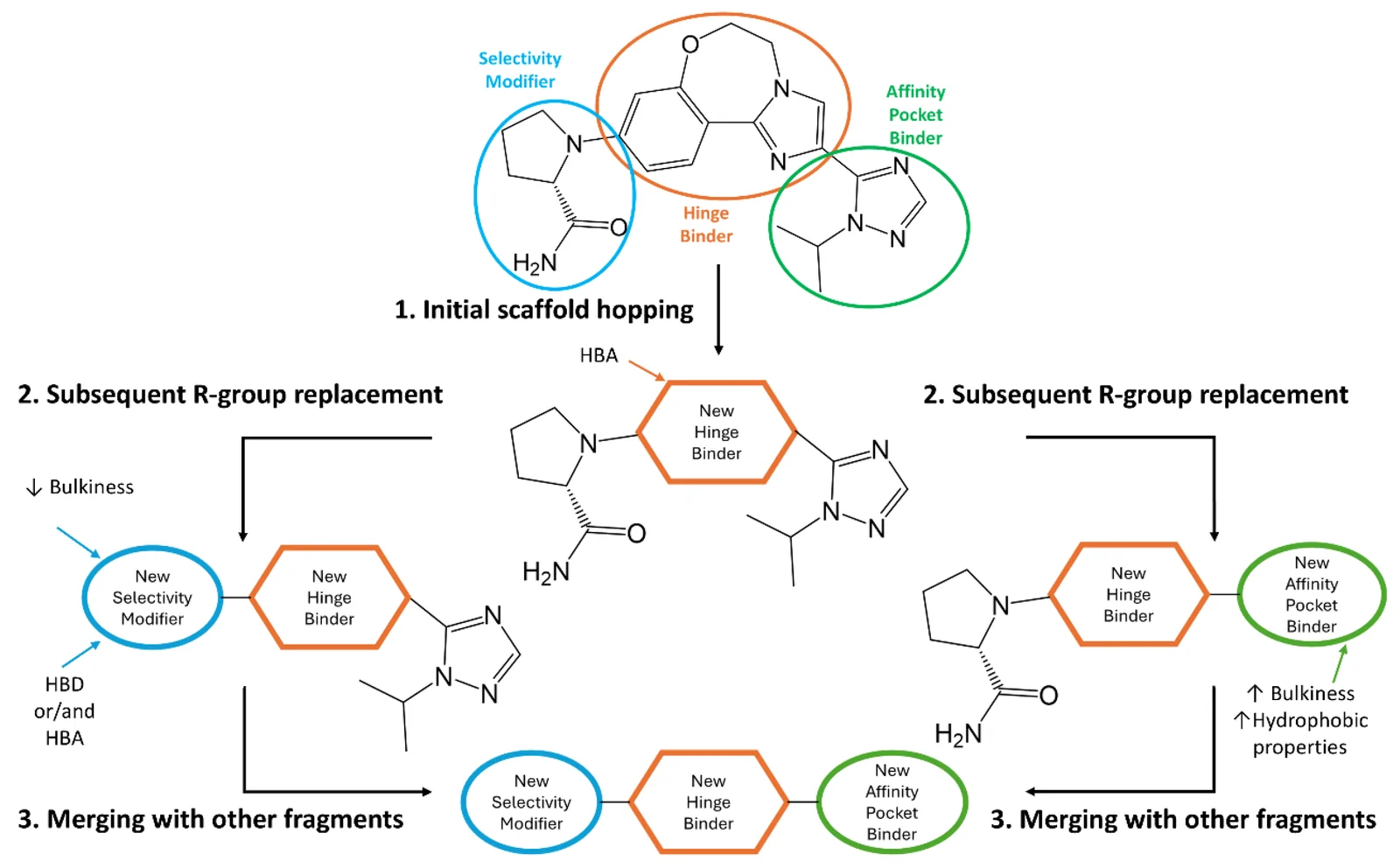
Template structure and subregions subjected to scaffold hopping and R-group replacement.

Based on binding site interactions, the **B1** structure was divided into three before-mentioned components—the “hinge binder”, the “affinity pocket binder”, and the “selectivity modifier”. A three-step approach was employed in the design of new inhibitors: first, hinge binder scaffold hopping; second, R-group replacement of the activity pocket binder and selectivity modifier; and finally, the merging of all identified moieties (Figure 12).

The first step involved searching for a new hinge binder scaffold. In this search, field (the new scaffold must interact with the field at roughly the same spatial location) and pharmacophore (the new scaffold must possess similar hydrogen bond acceptor properties) constraints for the hydrogen bond acceptor were established to identify moieties that effectively bind to αVal851 in the hinge region.

Initial scaffold searching gave a series of scaffolds that were incorporated into the rest of the structure. Compounds comprising the first 50 hinge binders with the highest-ranking score were prepared and then subjected to the ensemble docking procedure in all four PI3K isoforms. Compounds with scaffolds found to maintain a hydrogen bond connection to αVal851 were tested using the previously developed 3D-QSAR models to predict their activity and potential selectivity.

The newly designed compounds were analyzed for their active site interactions and compared to the lead compound **B1**, acting as a positive control. It was found that 24 compounds retain a hydrogen bond with αVal851 after docking, as well as a hydrogen bond with αGln859, which is important for their selectivity towards the α isoform. Further activity prediction using the developed 3D-QSAR models indicates that six of these compounds (Figure S11) exhibit PI3Kα selectivity with at least a 10-fold difference in activity (Table S8). The selected scaffolds can be categorized as derivatives of chromeno[3,4-d]imidazole (**H1** and **H2**), 2H-benzo[b][1,4]oxazine (**H3**), quinoline (**H4**), 3-methoxypyridine (**H5**), and anisole (**H6**).

Subsequent R-group replacement analysis was performed to search for new affinity pocket binders and selectivity modifiers. For the selectivity modifiers, both field and pharmacophoric constraints were set for hydrogen bond donors and acceptors to find new moieties that bind to αGln859, thus maintaining or even improving the selectivity of the newly designed compounds. Fragments satisfying the three structural requirements summarized above were retained for the subsequent combination and ensemble docking stages. This outcome validates the scaffold hopping approach as a viable means to discover new inhibitor chemotypes with retained or enhanced selectivity features.

The 20 highest-ranking affinity pocket binders and selectivity modifiers were combined with the six chosen hinge binders and subsequently with each other as well (Figure 12) to form new compounds, which were also subjected to ensemble docking. Based on the formed favorable interactions in the PI3Kα active site and activity predictions, 23 designed compounds (Figure S12) were selected as representative potential selective PI3Kα inhibitors (Table S9).

The final selection of affinity pocket binders includes various 1,2,4-triazole derivatives, connected either at position 1′ or 2′ to the hinge binder (Figure S12). The substituents have similar or more voluminous properties that enhance the steric properties described by the 3D-QSAR models, promoting PI3Kα activity. These substituents, alongside the ring itself, form multiple hydrophobic contacts within the affinity pocket within the active site of the enzyme (Table S11).

Three selectivity modifiers demonstrated favorable predicted docking poses and were substantiated by 3D-QSAR-predicted activities. These modifiers include derivatives of amide (**D13** and **D14**), urea (**D16**, **D18**, **D19**, **D20**, **D22** and **D23**), and carbonohydrazone diamide (**D15**, **D17** and **D21**) (Figure S10). Each compound forms at least one hydrogen bond with αGln859, prioritizing inhibitory activity towards the PI3Kα isoform (Table S9).

All designed compounds were tested to see whether they fit into the previously defined AD (Figure S13).

The predicted p*K*_i_ values for the newly designed PI3K inhibitors indicate a strong preference for the PI3Kα isoform, aligning with the design goal of achieving isoform selectivity. Most compounds exhibited high predicted affinities toward PI3Kα, with several reaching values above 9.5, suggesting nanomolar potency. In particular, compounds **D13**, **D1**, **D5**, **D2**, **D4** and **D3** emerged as the most potent candidates.

A comparison of predicted affinities across isoforms reveals that many compounds display significant selectivity for PI3Kα. For instance, **D13** shows a large selectivity window over PI3Kβ (Δp*K*_i_ ≈ 3.8), while **D1** is highly selective over both PI3Kγ and PI3Kβ. Other compounds such as **D3**, **D8**, and **D14** also demonstrated favorable selectivity profiles, with Δp*K*_i_ values commonly exceeding 2 over the closest off-target isoform. Several additional compounds (**D6**, **D7**, **D9**, and **D11**) also maintained good predicted affinity toward PI3Kα, though with more moderate selectivity margins compared to the top candidates. These molecules may still serve as promising derivatives but could require further structural refinement to reduce residual activity at the PI3Kβ or PI3Kδ isoform.

Compounds in the **D15**–**D22** series maintained selectivity profiles with moderate predicted affinities, highlighting their promise as well-designed candidates for further optimization as PI3Kα inhibitors. Compound **D23** demonstrated moderate activity and exhibited strong selectivity across all other isoforms, with the smallest difference being Δp*K*_i_ ≈ 1.6 relative to PI3Kδ. A few compounds, including **D10** and **D11**, showed more balanced affinities across all isoforms, which may indicate a risk of off-target activity.

These findings suggest that the top-performing compounds, like **D1**, **D2**, **D3**, **D4**, **D5, D13**, and **D23,** may serve as promising leads for further optimization.

**Figure 13:**
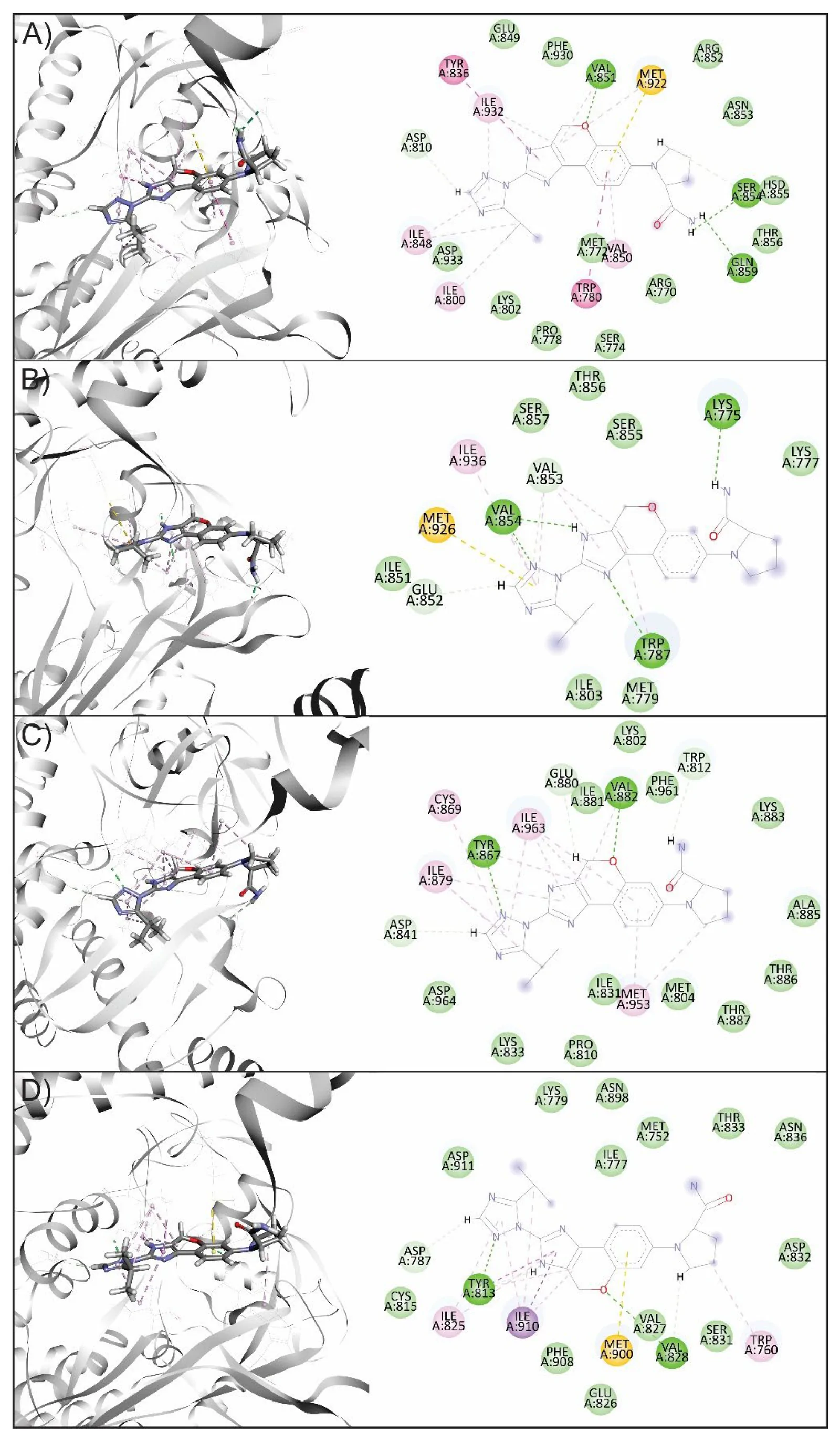
The 3D (**left**) and 2D (**right**) representation of the bioactive conformations of **D1** and interactions with the (**A**) PI3Kα, (**B**) PI3Kβ, (**C**) PI3Kγ, and (**D**) PI3Kδ isoforms; dark green—conventional hydrogen bonds, light green—carbon hydrogen bonds, medium green—van der Waals interactions, purple—T-shaped π-π interactions, pink—alkyl and π–alkyl interactions, and yellow—π–sulfur interactions.

The compound with one of the highest predicted PI3Kα activities, **D1**, forms three hydrogen bonds and multiple hydrophobic contacts in this isoform (Figure 13). The hinge binding motif (3,4-dihydrochromeno[3,4-d]imidazole) oxygen acts as a hydrogen bond acceptor in a bond with αVal851. The affinity pocket binder—the triazole ring—and adjacent isopropyl group also form a carbon–hydrogen bond with αAsp810, along with numerous hydrophobic interactions with αMet772, αPro778, αIle800, αLys802, αIle848, and αMet922, which align with the 3D-QSAR studies indicating the importance of this hydrophobic moiety (var15, var291, var397) with a connected voluminous substituent (var397). The amide group connected to the pyrrolidine ring forms two hydrogen bonds with αGln859 and αSer854.

When bound to the β isoform, **D1** behaves similarly to the lead compound, binding to the βVal854 in the hinge region with the imidazole ring; thus, it does not fully reach the affinity pocket and forms fewer hydrophobic contacts, βGlu852, βVal853, βMet926, and βIle936, explaining its low predicted PI3Kβ activity. The amide group binds to βLys775 but does not interact with βAsp862, the analogue of αGln859.

The hinge binder oxygen binds to σVal882 in the PI3Kγ isoform, forming hydrophobic contacts with σVal882, σMet953, and σIle963. The triazole ring also binds to σIle963 and further into the affinity pocket to σTyr867, σCys869, and σIle879. The amide group lacks hydrogen bonds.

The chromen oxygen also forms a hydrogen bond with δVal828 in the hinge region. The nitrogen at the 2′ position of the triazole rings forms a hydrogen bond with δTyr813, and the ring and isopropyl group form hydrophobic alkyl contacts with δIle910, δTyr813, and δIle825. The pyrrolidine ring binds to δTrp760. The suboptimal binding modes of **D1** in the PI3Kβ and PI3Kγ isoforms could explain its lower predicted activities determined in the 3D-QSAR study.

Compound **D19**, a lower-performing designed compound, has lower predicted PI3Kα activity but retains selectivity against other isoforms. In the PI3Kα active site, the quinoline hinge binder’s nitrogen forms a hydrogen bond with αVal851. A carbon–hydrogen bond with αVal850 can also be noticed. The quinoline ring also forms a T-shaped π-π bond with αTrp780 and multiple hydrophobic interactions with different amino acids, including αIle800, αMet922, and αIle932. The affinity pocket binder forms an alkyl bond with αIle848, which aligns with lower predicted α activity. The urea forms two hydrogen bonds with αSer854 and one with αGln859.

In a complex with PI3Kβ, the quinoline nitrogen binds to the hinge region with a hydrogen bond to the βVal854 amino acid, a carbon–hydrogen bond with βTrp787, and a π–alkyl bond with βVal853. The triazole ring forms two hydrophobic contacts with βMet779 and βIle803, while the isopropyl group binds to βIle936. The urea forms two hydrogen bonds with βSer857 and one with βGlu857.

When bound to the σ isoform, the quinoline binds to σVal882 via a π–alkyl bond only, while the urea forms a hydrogen bond with it. Other hydrophobic contacts formed by this hinge binder are with σIle963, σIle963, σTyr867, and σMet953. The urea also forms a hydrogen bond with σAla885. The triazole’s 3′ nitrogen and hydroxyl group oxygen form two hydrogen bonds, while the hydroxyl group hydrogen forms a hydrogen bond with σAsp964. The ring also forms a π–alkyl bond with σIle879, while the isopropyl group forms two alkyl bonds with σIle831 and σIle879. The distorted binding mode of the compound in the PI3Kσ active site corresponds with very low predicted PI3Kσ activity.

The PI3Kδ-D19 complex contains one conventional hydrogen bond and a carbon–hydrogen bond with δVal828 in the hinge region. A T-shaped π-π interaction is formed between the pyridine ring and δMet900. Other hydrophobic contacts include interactions with δLys833, δVal827, and δIle910. The ethyl group forms four alkyl contacts with δTyr813, δIle825, δVal828, and δIle910. The δTrp760 amino acid also forms one T-shaped π-π interaction with the triazole ring and an alkyl interaction with the isopropyl group.

### 2.5. Quantum Chemical Characterization of Selected PI3Kα Inhibitors

To gain deeper insight into the electronic properties, electron density, and charge distribution of the designed compounds [28], which may influence their reactivity patterns relevant for intermolecular interactions, a density functional theory (DFT) study was performed on a representative subset of inhibitors. The calculated frontier molecular orbital energies (E_HOMO_ and E_LUMO_), molecular electrostatic potential extrema (MEP_min_ and MEP_max_), and quantum chemical reactivity descriptors are summarized in Table 3, indicating moderate variation in the electronic properties across the investigated dataset. Namely, variations in orbital energies, E_HOMO_ (from −7.610 eV (**D23**) to −4.784 eV (**D1**)) and E_LUMO_ (from −0.312 eV (**B1**) to −2.558 eV (**D23**)) point to differences in electron-donating and electron-accepting capacities within the dataset. Consequently, the energy gap (ΔE) [29] varies between 3.610 eV (**D4**) and 5.052 eV (**D23**), suggesting that **D4** may be associated with increased chemical softness and reactivity, whereas **D23** exhibits a more pronounced electronic stability.

Compound **B1**, identified as the most active entity in the dataset and used as a template for the design, exhibited the highest E_LUMO_ value and one of the highest E_HOMO_ values among the compounds analyzed by DFT. This electronic profile suggests a relatively reduced electron-accepting ability combined with a favorable electron-donating capacity, which may indicate a propensity for donor-type intermolecular interactions and moderate overall reactivity [29].

**Figure 14:**
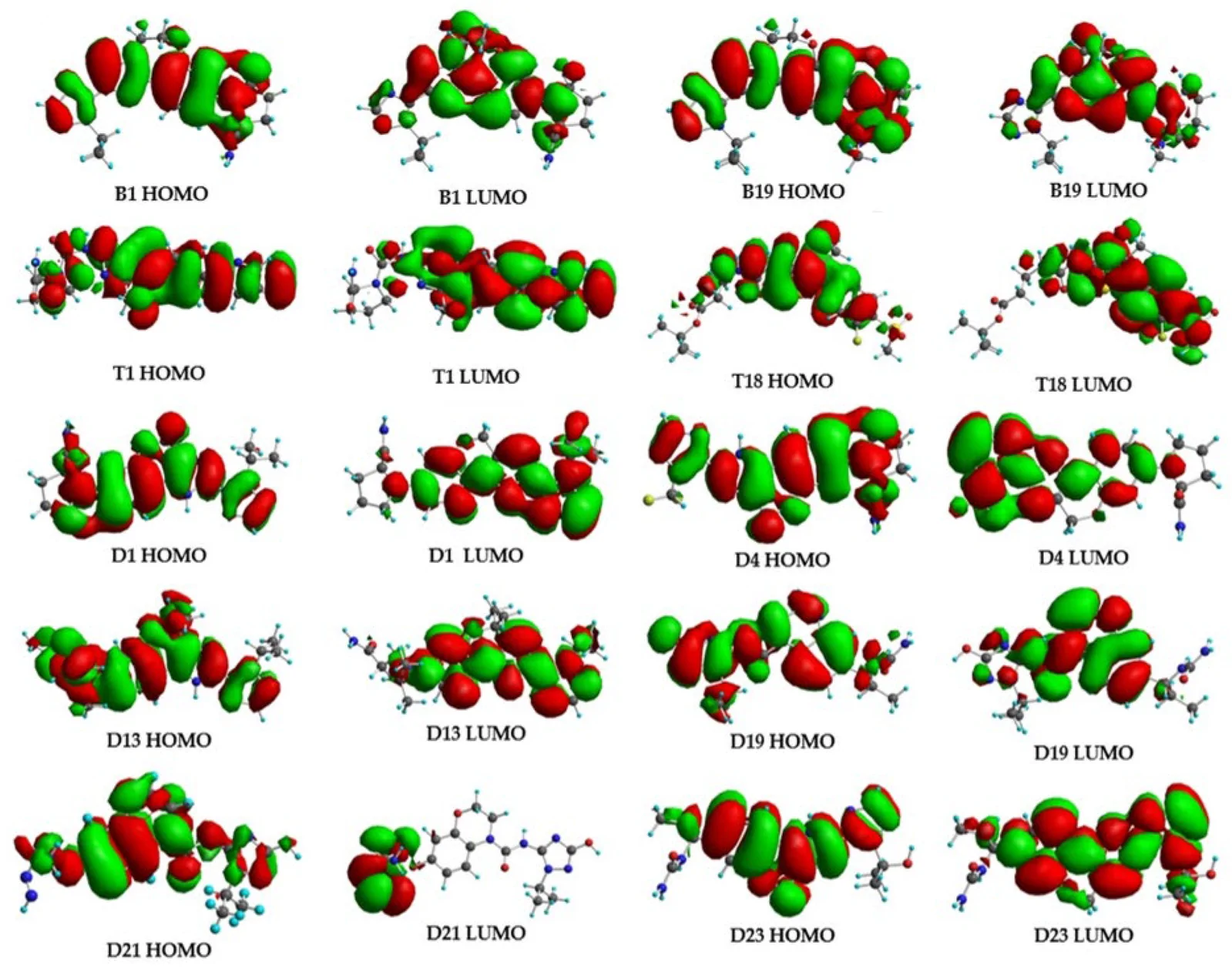
Spatial distributions of the highest occupied molecular orbital (HOMO) and lowest unoccupied molecular orbital (LUMO) of the investigated compounds. Red and green isosurfaces represent opposite phases of the molecular-orbital wavefunction.

Molecular orbital distributions (Figure 14) reveal distinct patterns within the investigated compounds. In compounds **B1**, **B19**, and **D19**, both HOMO and LUMO are largely delocalized over similar regions of the molecules, suggesting a relatively uniform electron density distribution. Compounds **D1**, **D4**, **D13**, **D23**, **T1**, and **T18** exhibit mainly delocalized molecular orbitals; however, a moderate spatial differentiation between HOMO and LUMO can be observed, indicating enhanced electronic polarization [29] in relation to more uniformly distributed systems. A particularly distinct distribution is observed for compound **D21**, where LUMO is localized on the carbonohydrazone diamide moiety, making it the main electrophilic center and the most prominent electron-accepting region of the molecule.

The molecular electrostatic potential (MEP) values presented in Table 3 show a relatively wide range of both negative (MEP_min_) and positive (MEP_max_) electrostatic regions in the investigated compounds, indicating differences in charge distribution within the dataset. The most pronounced extrema were observed in compounds **D23** and **T18**, which exhibit both the most negative MEP_min_ values (−59.27 and −51.43 kcal/mol, respectively) and the most positive MEP_max_ values (58.91 and 66.42 kcal/mol, respectively), indicating a highly polarized electronic structure [30]. Compound **D23** expresses moderate activity and strong selectivity across all other isoforms, with the smallest difference being Δp*K*_i_ ≈ 1.6 relative to PI3Kδ. Compound **T18** was identified as the least active Thz cluster compound. In addition, compound **D21** exhibits the least negative MEP_min_ value (−45.06 kcal/mol), indicating a reduced extent of electron-rich regions, while **B19** displays the lowest MEP_max_ value (34.69 kcal/mol), suggesting less pronounced electron-deficient sites. Furthermore, compound **B19** was previously identified as the least active Bzo cluster compound in the PI3Kα 3D-QSAR model. In contrast, the reference compound **B1** exhibits moderate MEP extremes (−46.19 and 51.15 kcal/mol), reflecting a more balanced charge distribution. Although different regions capable of participating in electrostatic interactions were identified in the set of compounds tested, certain derivatives, such as **D23** and **T18**, exhibit a higher degree of polarization, which may affect their patterns of intermolecular interactions.

**Figure 15:**
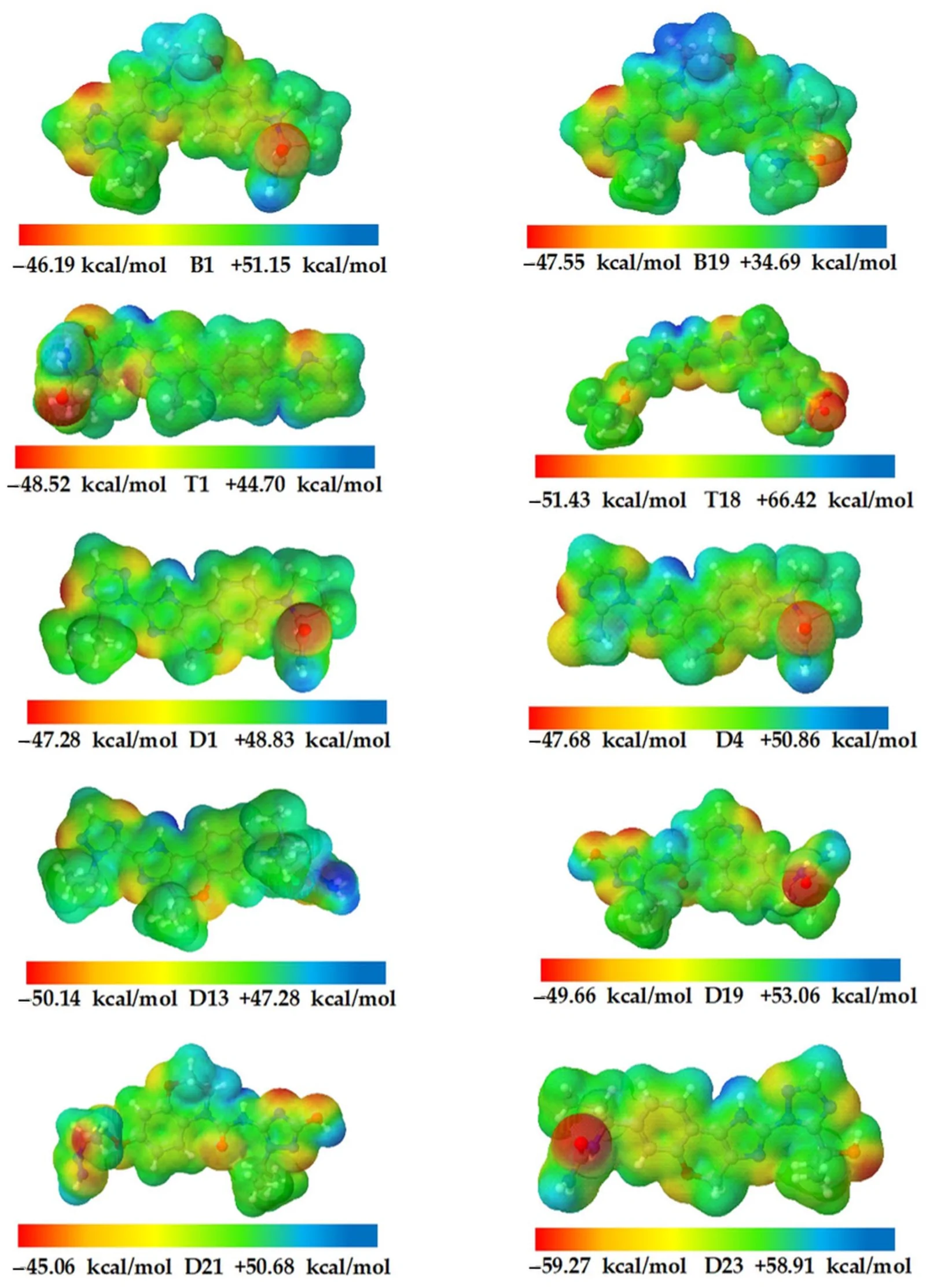
ESP of selected PI3K inhibitors analyzed in the DFT study. Negative electrostatic potential regions are depicted in red, while positive electrostatic potential regions are depicted in blue.

Visualization of the electrostatic potential surfaces further supports these observations (Figure 15). In all examined compounds, negative electrostatic potential (depicted in red) is predominantly localized around heteroatoms such as oxygen and nitrogen within amide, urea, and triazole moieties, identifying these regions as potential hydrogen bond acceptors. Conversely, positive electrostatic potential (depicted in blue) is mainly distributed over the hydrogen atoms and less electron-rich regions of the analyzed molecules. If these findings are considered alongside the docking results, it can be observed that regions of negative electrostatic potential are mainly localized on oxygen and nitrogen atoms within the amide, urea, and triazole moieties, corresponding to functional groups involved in forming hydrogen bonds with key amino acid residues such as αVal851 and αGln859. This can be clearly illustrated with reference compound **B1**. Regions of negative electrostatic potential are primarily localized on heteroatoms such as carbonyl oxygen and heterocyclic nitrogen atoms, which can act as hydrogen bond acceptors, while regions of positive potential are associated with the hydrogen atoms of the amide group, capable of acting as hydrogen bond donors. These functional groups may participate in hydrogen bonding interactions with key residues such as αVal851, αSer854, and αGln859, as observed in the docking analysis.

The values of remaining quantum chemical descriptors in Table 3 further indicate the observed differences in electronic behavior within the analyzed dataset. The values of the energy gap (ΔE) show that most compounds exhibit moderate chemical stability [29], with **D4** having the smallest gap, indicating increased reactivity, and **D23** having the highest ΔE, showing its rigid electronic structure. Chemical hardness (*η*) and softness (*S*) follow the same trend, with compounds such as **D4** and **D1** displaying greater *S*, indicating a greater ability to adjust their electron density during intermolecular interactions [31]. The electrophilicity index (ω) is particularly high for **D19** and **D23**, reflecting their enhanced electron-withdrawing characters [32], while compounds such as **B1** and **D1** show more balanced reactivity profiles. Additionally, despite its overall electronic stability (the highest ΔE), compound **D23** possesses strongly polarized regions capable of localized intermolecular interactions.

Considering the values in Table 3 in relation to the docking results, it may be inferred that efficient binding depends not only on overall reactivity but also on the balance between electronic flexibility and the presence of well-defined donor and acceptor regions. Compounds such as **D1** and **D4**, which have lower ΔE and greater *S*, can more readily adjust their electron density to accommodate hydrogen bonds and hydrophobic interactions within the PI3Kα active site. In contrast, although **D23** exhibits pronounced polarization, its greater rigidity may restrict the optimal alignment of interacting groups, despite the presence of favorable electrostatic regions. This is consistent with its moderate activity (Δp*K*_i_ ≈ 1.6 relative to PI3Kδ), as predicted in the docking study, and a more favorable selectivity profile across all isoforms. Similarly, the increased electrophilicity observed for **D19** and **D23** indicates a stronger electron-withdrawing character, which may contribute to specific interactions with electron-rich residues, but does not necessarily result in more favorable overall binding patterns.

Therefore, **B1**, **D1**, and **D4** combined high predicted p*K*_i_ values with intermediate-to-low ΔE and moderate-to-high *S* values, consistent with sufficient electronic adaptability for favorable interactions within the PI3Kα binding site. In contrast, **D23** combined the highest Δ*E* and lowest *S* with moderate predicted PI3Kα activity but favorable isoform selectivity, suggesting that electronic stability may support a selective interaction pattern even when it is not associated with maximal predicted potency.

### 2.6. Structure-Based Virtual Screening Results

To identify new kinase inhibitors with activity and potential selectivity towards PI3Kα, a structure-based virtual screening (SBVS) approach was employed using the LDA-based protocol. Virtual screening was employed as a key strategy in this study to explore a broader range of chemical scaffolds and enhance the identification of novel PI3Kα inhibitors. Traditional drug discovery efforts often focus on limited regions of the chemical space defined by known active compounds, which can constrain the discovery of structurally novel and potentially more effective inhibitors. By applying virtual screening to large and diverse compound libraries, we aimed to overcome this limitation and significantly expand the chemical space sampled during the identification of possibly active compounds. This approach allowed us to explore new molecular frameworks and pharmacophore features not represented in currently known PI3Kα inhibitors, thereby increasing the likelihood of identifying unique and potent chemotypes. The results below detail the outcomes of this virtual screening, including the prioritization of top-ranked hits and their predicted interactions with the PI3Kα binding site.

**Figure 16:**
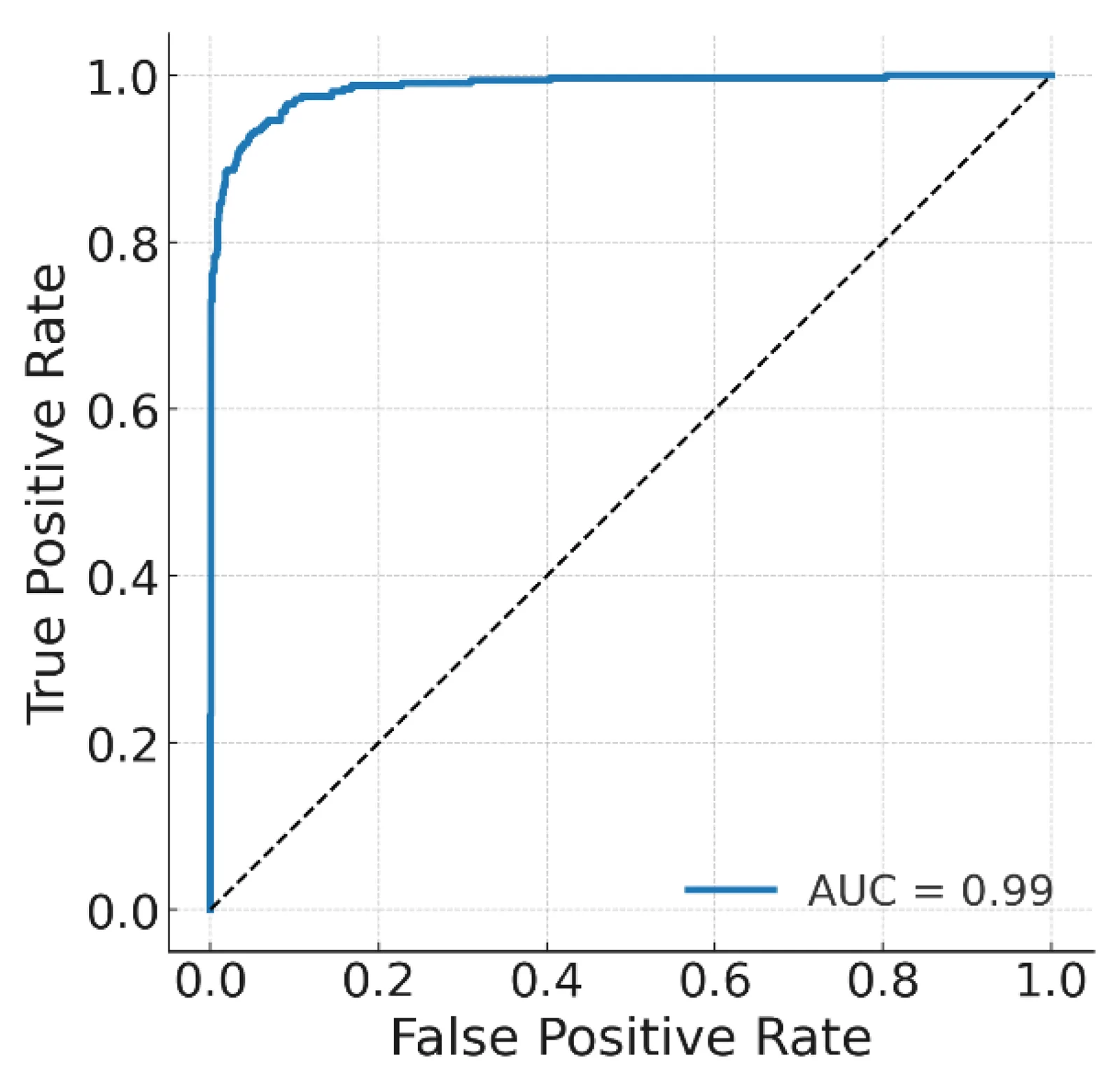
The ROC curve of the LDA-SBVS model (AUC = 0.99). The dashed diagonal line represents the no-discrimination reference corresponding to random classification (AUC = 0.50).

The final 318 actives and 2178 inactives were used in the procedure. The protocol selected three centroids (αBzo1, αThz2, and αThz4) (Table S1) obtained from MD simulations as templates for the SBVS procedure. The H and DRY-O scores were used in the LDA model. The overall AUC was 0.99, and enrichments at 0.5%, 1%, 2%, and 5% were 0.78, 0.83, 0.89, and 0.93, respectively (Figure 16).

The developed model was used to screen the enamine kinase inhibitors library. The top 30 predicted active compounds were subjected to the ensemble docking method. The compounds were compared to the lead compound **B1**, acting as a positive control, and those with favorable docking poses were further subjected to 3D-QSAR activity prediction (Figure S14). The final eight compounds were selected for further analysis (Table S10).

These compounds were also analyzed in a Williams plot to ensure they fit into the previously defined AD (Figure S15).

These compounds exhibit interactions with the hinge region, forming a hydrogen bond with αVal851. They also form a hydrogen bond with a non-conserved amino acid. Compounds **VS1**, **VS3**, and **VS8** form a hydrogen bond with αGln859. All three compounds contain an amide group responsible for this interaction. Compounds **VS2**, **VS4**, **VS5**, and VS7 form a hydrogen bond with αAsn853; compound **VS4** also forms a hydrogen bond with αHis855 from the hydrophobic region II; and **VS6** forms a bond with αArg770 located at the P-loop.

Out of the selected compounds, **VS1** has the highest LDA score and the highest predicted PI3Kα p*K*_i_ value. The amine hydrogen, interconnecting piperidine and thienopyrimidine, binds to αVal851. The carbonyl oxygen of the amide group forms a hydrogen bond with αGln859, giving it the potential for selectivity towards the α isoform. The thienopyrimidine ring forms different hydrophobic contacts, including π–alkyl interaction with αVal850 in the hinge region, π–sulfur and T-shaped π-π interactions with αTyr836, and π–alkyl interactions with αIle932 and αMet922 in the affinity pocket.

The p*K*_i_ values of the screened compounds were evaluated towards other isoforms (PI3Kβ, PI3Kσ, and PI3Kδ) to assess their selectivity profiles. Among the eight compounds analyzed, **VS1** and **VS2** demonstrated the highest predicted selectivity for the PI3Kα isoform. **VS1** exhibited a p*K*_i_ of 8.29 for PI3Kα, with lower predicted affinities for PI3Kβ, PI3Kσ, and PI3Kδ, corresponding to a > 100-fold difference between PI3Kα and PI3Kβ. Similarly, **VS2** showed a p*K*_i_ of 7.39 for PI3Kα, with selectivity of Δp*K*_i_ > 1 relative to PI3Kβ and PI3Kδ, suggesting a strong isoform preference.

In contrast, compounds **VS4**, **VS5**, and **VS6** demonstrated higher predicted affinities for PI3Kδ than for PI3Kα, indicating poor selectivity for the PI3Kα isoform. Specifically, **VS4** had a p*K*_i_ of 8.57 for PI3Kδ compared to 7.93 for PI3Kα, suggesting a δ-selective profile.

Compounds **VS3**, **VS7**, and **VS8** displayed moderate activity toward PI3Kα, with modest selectivity over PI3Kβ (Δp*K*_i_ ≈ 1 in most cases). However, their predicted activities toward the PI3Kσ and PI3Kδ isoforms were comparable to those for PI3Kα, indicating limited isoform discrimination.

Taken together, these results suggest that **VS1** and **VS2** represent the most promising candidates for selective inhibition of PI3Kα (Table S10). The remaining compounds either lack sufficient selectivity or exhibit preferential activity toward other PI3K isoforms, particularly PI3Kδ, and may require further structural optimization to improve α-selectivity.

These findings offer preliminary evidence for chemical types that may preferentially target PI3Kα over other isoforms. However, as this is only the first step in the identification of selective inhibitors, further studies, both in silico and experimental, are necessary to validate these interactions, assess biological activity, and optimize selectivity and pharmacological properties.

### 2.7. In Silico ADMET Profiling of the Dataset, Designed and VS Compounds

Various physicochemical parameters, synthetic accessibility scores (Table S11), and pharmacokinetic properties (Table S12) were calculated for the in silico evaluation of the newly designed inhibitors. Lipophilicity features were assessed using MlogP values, which indicated that all compounds, including **D1**, **D2**, **D3**, **D4**, **D5**, **D13**, **D23**, **VS1**, and **VS2**, possess optimal permeability and solubility profiles, suggesting good oral bioavailability. Lipinski’s “rule of five” was satisfied for most molecules, including **D1**, **D2**, **D3**, **D4**, **D5**, **D13**, **D23**, **VS1**, and **VS2**. Regarding plasma protein binding, these compounds exhibited unbound drug percentages in plasma within a similar range to **B1**.

Metabolic liability was also examined. **D3** and **D13** possess a higher CYP-related risk than **B1**, suggesting potentially faster clearance, whereas **D1**, **D2**, **D4**, **D5**, **D23**, **VS1**, and **VS2** show metabolic profiles more optimal compared to the lead. All these compounds, like **B1**, were identified as P-gp substrates.

Toxicity predictions highlighted potential hERG inhibition for **D4** and **D13**, in contrast to **B1**, while **D1, D2, D3, D5**, **D23, VS1,** and **VS2** did not raise significant cardiovascular safety concerns. **D2**, **D3,** and **VS2** were flagged for a higher overall toxicity risk than **B1**, whereas **D1**, **D4**, **D5**, **D13**, **D23**, and **VS1** remained within a safer range.

Synthetic accessibility scores revealed that **D1** is more synthetically challenging than **B1**, reflecting greater molecular complexity. **D2**, **D3**, **D4, D5**, **D13**, **D23**, **VS1**, and **VS2**, however, show synthetic difficulty scores lower than the lead compound, suggesting an easier synthesis procedure.

Among the compounds with more favorable predicted activity and selectivity profiles, **D1** and **D5** showed more balanced predicted ADMET profiles, combining favorable permeability, solubility, and metabolic properties without identifying possible hERG-related problems, whereas **D13** retained favorable permeability and solubility but showed higher predicted CYP-related liability and potential hERG inhibition.

**Figure 17:**
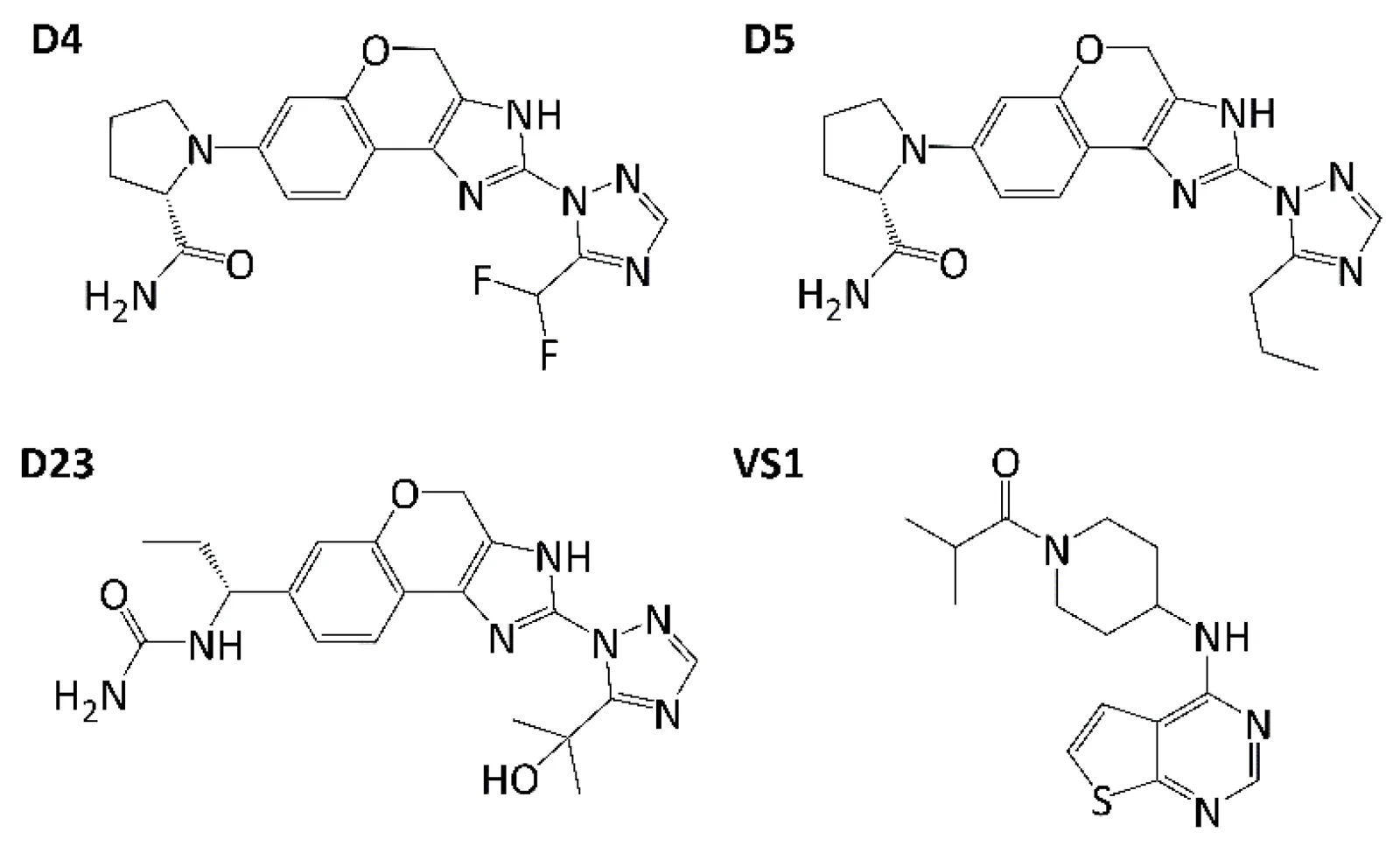
Structures of the most promising compounds for further optimization.

**Figure 18:**
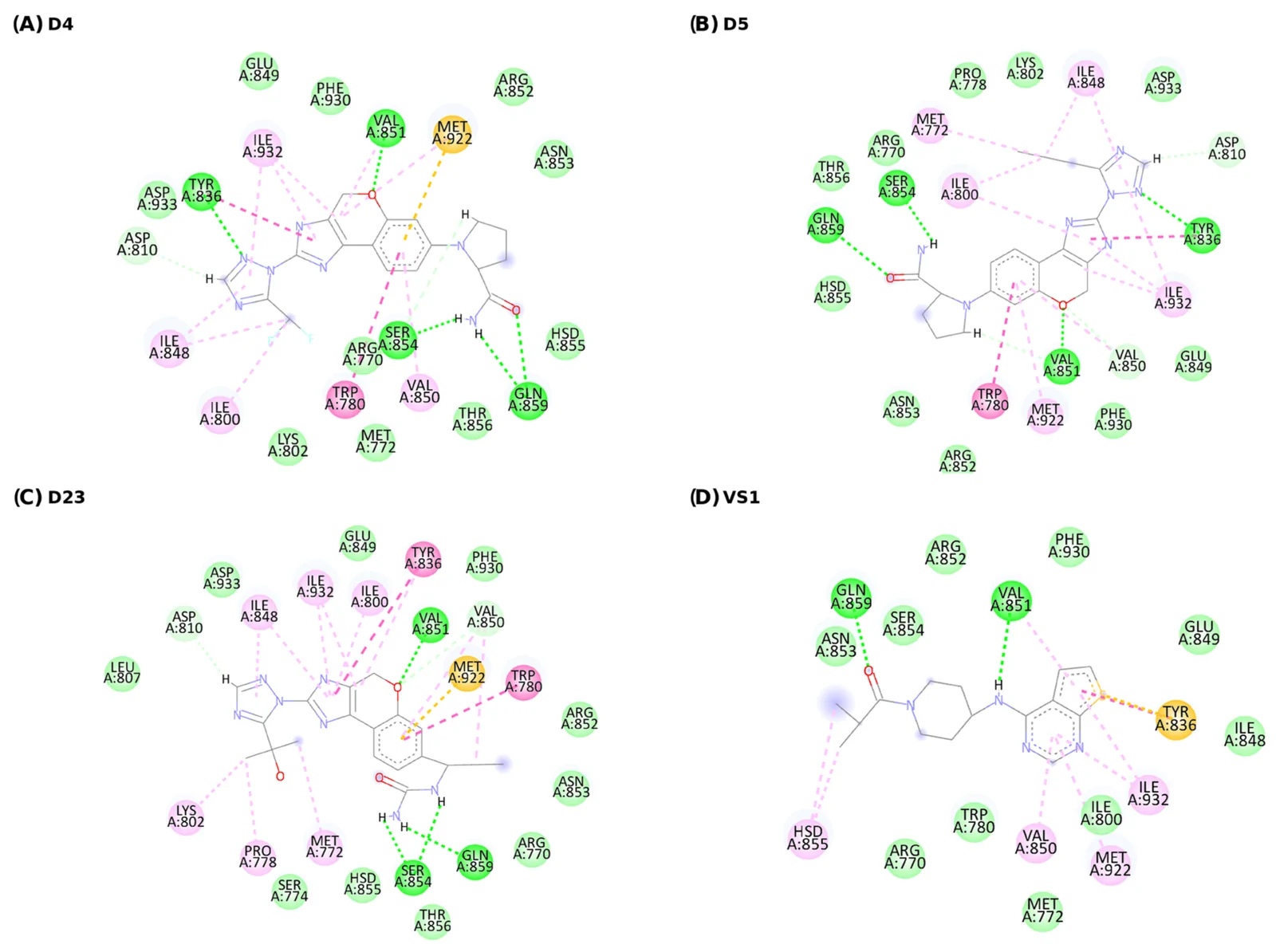
The 3D–2D representation of the bioactive conformations and interactions of (**A**) **D4**, (**B**) **D5**, (**C**) **D23**, and (**D**) **VS1** with the PI3Kα isoform; dark green—conventional hydrogen bonds, light green—carbon–hydrogen bonds, medium green—van der Waals interactions, purple—T-shaped π-π interactions, pink—alkyl and π–alkyl interactions, and yellow—π–sulfur interactions.

Taken together, compounds **D1**, **D13**, **D23**, **VS1**, and **VS2** exhibit drug-like properties broadly comparable to **B1**, with **D13** distinguished by higher metabolic and hERG-related risks, **D1** by greater synthetic difficulty, and **D2**, **D3**, and **VS2** by increased toxicity concerns. Conversely, **D4**, **D5**, **D23,** and **VS1** stand out as structurally accessible candidates with balanced ADMET profiles, making them promising alternatives for further optimization (Figure 17 and Figure 18 and Table 4). Compound **D23** combined high predicted PI3Kα activity and selectivity with acceptable synthetic accessibility and no major predicted ADMET liability. Compounds **D4** and **D5** showed similarly favorable activity–selectivity profiles but slightly less balanced pharmacokinetic predictions, whereas compound **VS1** retained acceptable drug-likeness and safety characteristics but had lower predicted PI3Kα activity. Overall, **D23** was the most balanced candidate, followed by **D4** and **D5**, with **VS1** ranked lower.

The PAMPA-SVM model [33] was utilized to further evaluate these compounds for their potential ability to cross the blood–brain barrier (BBB). Based on logPe values, compounds **VS1**, **VS8**, **D18**, and **D20** demonstrate low permeability; compounds **B1**, **H5**, **D14**, **D16**, **D17**, **D19**, **D21**, **D22**, **D23**, **VS5**, **VS6**, and **VS7** demonstrate medium permeability; and compounds **H1**, **H2**, **H3**, **H4**, **H6**, **D1**, **D2**, **D3**, **D4**, **D5**, **D6**, **D7**, **D8**, **D9**, **D10**, **D11**, **D12**, **D13**, **D15**, **VS2**, **VS3**, and **VS4** demonstrate high permeability across the BBB (Table S13). All compounds fit into the PAMPA-SVM model’s AD (Figure S16).

Predicted BBB permeability should be interpreted in relation to the intended therapeutic indication. For peripheral solid tumors, limited or moderate BBB permeability may be preferable because substantial CNS exposure is not required and could increase the risk of central adverse effects. Accordingly, the medium predicted permeability of **D23** and low permeability of **VS1** may be advantageous for peripheral antitumor therapy, whereas the high predicted permeabilities of **D4** and **D5** may require further optimization. Conversely, higher BBB permeability could be beneficial if these compounds were developed for primary CNS tumors or brain metastases.

### 2.8. Chemical Space Expansion of Designed and Virtually Screened Compounds

**Figure 19:**
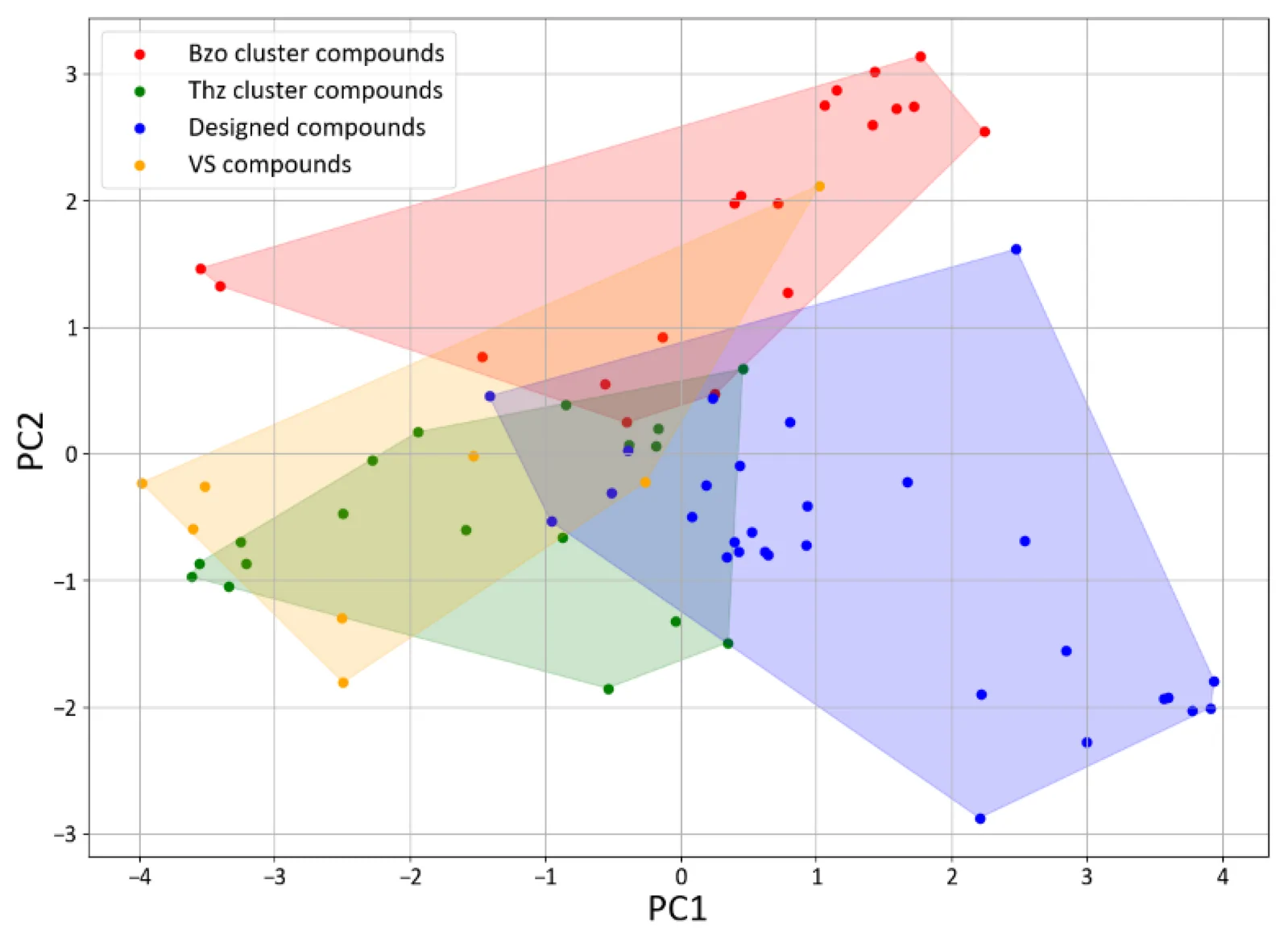
PCA plot of the dataset, designed and VS compounds based on all GRIND-2 3D-QSAR descriptors.

A principal component analysis (PCA) was performed based on all GRIND-2 descriptors from the 3D-QSAR analysis of compounds interacting with PI3Kα to investigate the chemical space covered by all examined compounds. The PCA was conducted using the first two principal components, which together define a plane summarizing the major variation in the data. The PCA plot shows that the four compound groups cover distinct regions of chemical space (Figure 19). The Bzo cluster compounds form a compact cluster, while the Thz cluster extends into new areas, adding diversity. The designed compounds cover the largest and most varied space, indicating high internal diversity. The VS compounds occupy a separate, unique region with some overlap with other clusters, also suggesting distinct chemical features. Overall, each group explores different parts of chemical space, which is important for increasing diversity in drug discovery and compound design.

**Figure 20:**
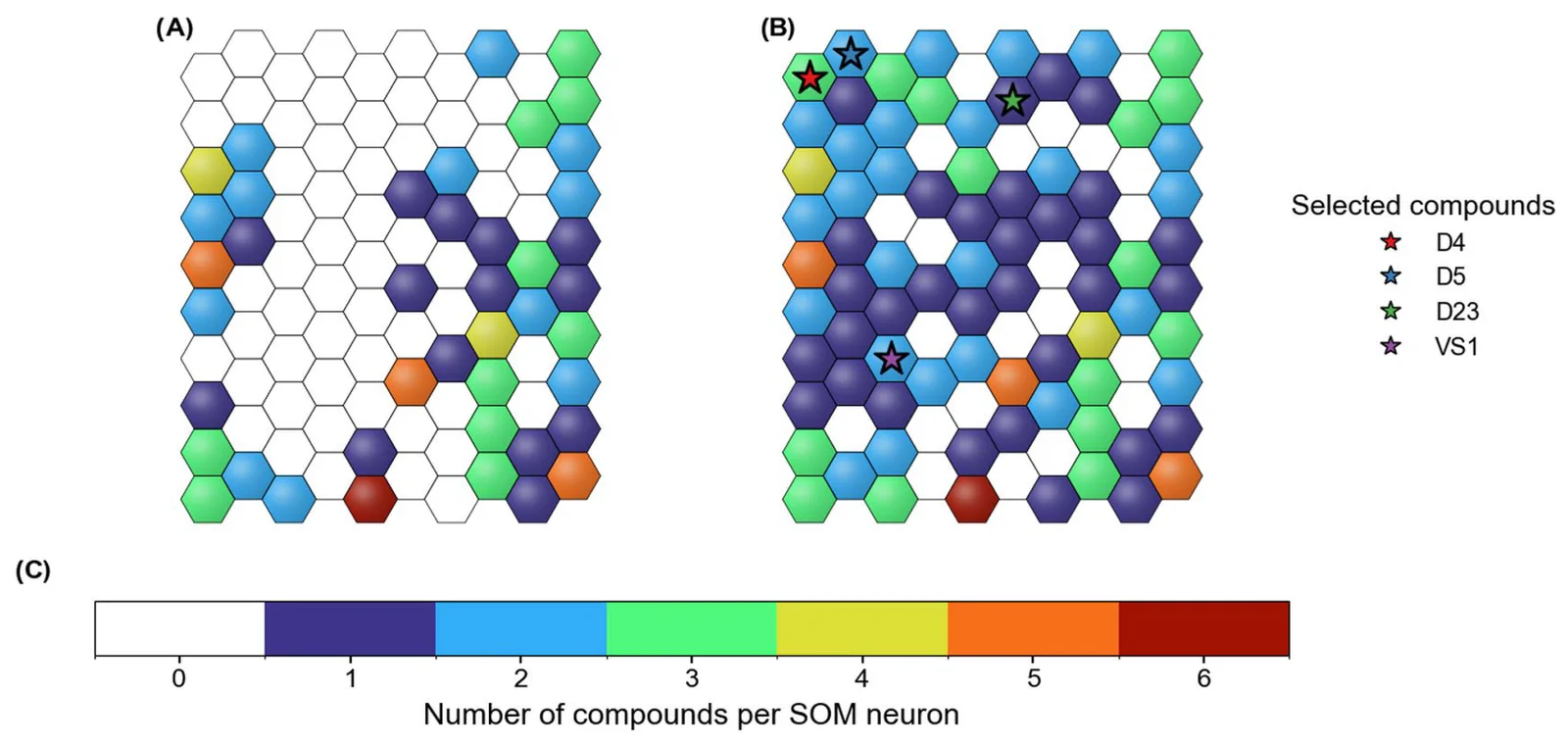
Self-organizing map (SOM) occupancy analysis of the PI3Kα inhibitor chemical space. (**A**) Distribution of known PI3Kα inhibitors. (**B**) Distribution after inclusion of designed compounds (D) and virtual screening (VS) hits. (**C**) Color scale indicating the number of compounds assigned to each SOM neuron.

To complement this GRIND-2-based representation, SOM analysis was performed using MACCS fingerprints as a 2D structure-based description of chemical space. The main SOM analysis confirmed that the designed and virtually screened compounds expanded beyond the regions predominantly occupied by known PI3Kα inhibitors (Figure 20). All designed D and H compounds were assigned to SOM neurons without known PI3Kα inhibitors, and the same was observed for 29 of the 30 top-ranked LDA-SBVS hits. The strongest novelty contribution was observed for the Top_LDA_VS group, since all compounds from this set showed maximum ECFP4 similarities below 0.50 relative to the known PI3Kα inhibitor set. Among the selected candidates, **D4**, **D5**, **D23**, and **VS1** were all located in neurons without known PI3Kα inhibitors and showed maximum ECFP4 similarities below 0.50, further supporting their structural distinction from the reference inhibitor space. The supplementary SOM analysis (Figure S15) performed with all available ChEMBL PI3Kα actives confirmed this trend, showing that the selected compounds remained positioned in under-represented regions even when a broader reference set of known inhibitors was used.

Together, these analyses indicate that the applied design and screening workflow did not merely reproduce known PI3Kα inhibitor-like structures but generated and identified compounds that occupy under-represented regions of PI3Kα inhibitor chemical space.

### 2.9. Representative Post-Design MD Assessment of Compound ***D23***

Compound **D23** was selected as a representative designed compound for additional post-design MD assessment based on its favorable predicted PI3Kα activity and selectivity, suitable docking interaction pattern across PI3K isoforms, and suitable physicochemical and ADMET profiles. This analysis was performed to evaluate whether the predicted PI3Kα binding mode was preserved during the simulation and to qualitatively compare the behavior of **D23** across the remaining class I PI3K isoforms. The PI3Kα-D23 complex is discussed in this manuscript, whereas the full comparative analysis is provided in the Supplementary Information.

The 100 ns MD simulation of the PI3Kα-D23 complex showed that the protein backbone and active site region remained stable during the analyzed trajectory (Figure S18). The ligand remained positioned within the ATP-binding site, with only moderate local rearrangement relative to the initial docking pose. The Cα RMSF profile showed no pronounced destabilization of the binding site region (Figure S19). In particular, the P-loop and the hydrophobic region II adjacent to the hinge region displayed limited fluctuations, supporting preservation of the local active site architecture during the simulation. The radius of gyration profile showed only minor variations and no progressive increase or decrease during the simulation, indicating preservation of the overall protein compactness and absence of major global structural disruption (Figure S20).

Analysis of the PI3Kα-D23 interaction pattern showed that the predicted binding mode can be rationalized through the contribution of the three designed structural elements: the hinge-binding scaffold, the affinity pocket binder, and the selectivity modifier. The chromeno[3,4-d]imidazole hinge-binding scaffold preserved the canonical hinge region interaction through close contact with αVal851. A direct hydrogen bond with the backbone NH group of αVal851 was observed recurrently during the trajectory, supporting retention of the main hinge binding interaction and the ability of **D23** to remain bound within the PI3Kα ATP-binding site.

The affinity pocket binder contributed mainly to the stabilization of **D23** within the hydrophobic part of the ATP-binding site. This fragment maintained favorable contacts with residues forming the affinity pocket and adjacent regions, including αMet772, αIle800, αIle848, and αMet922. These interactions are consistent with the design strategy and 3D-QSAR results, which emphasized the importance of hydrophobic and steric complementarity in this region for maintaining PI3Kα activity. Therefore, the affinity pocket binder appears to primarily support the overall binding affinity of **D23** by filling the hydrophobic pocket and stabilizing the ligand orientation relative to the hinge region.

In contrast, the urea-based selectivity modifier was oriented toward the hydrophobic region II adjacent to the hinge region. During the PI3Kα-D23 simulation, this fragment maintained favorable contacts with residues such as αSer854, αHis855, αAsn853, and αArg852. Their involvement suggests that the selectivity modifier contributes to the isoform-dependent binding behavior of **D23** by adapting to the shape and polarity of the PI3Kα-specific hydrophobic region environment.

The interaction between **D23** and αGln859 showed a dynamic character during the simulation, similar to that observed for the reference benzoxazepine compound **B1**. This suggests that αGln859 can contribute to ligand recognition, while additional polar and hydrophobic contacts with surrounding non-conserved or partially conserved residues in hydrophobic region II further support positioning of the selectivity modifier. Thus, the role of this fragment is not restricted to a single static αGln859 hydrogen bond; it involves a broader interaction pattern within the hydrophobic region II. Together with the conserved hinge region interaction and affinity pocket contacts, this interaction pattern supports the predicted PI3Kα selective binding profile of compound **D23**.

The comparative simulations with PI3Kβ, PI3Kγ, and PI3Kδ provided additional context for the interpretation of the potential **D23** selectivity profile. In PI3Kβ and PI3Kδ, **D23** showed more pronounced ligand repositioning and less stable preservation of the canonical hinge-binding pattern, suggesting less favorable positioning in these off-target isoforms. In contrast, the PI3Kγ complex retained stable hinge binding. Therefore, the representative MD analysis supports the stability of the predicted **D23** binding mode in PI3Kα, while the selectivity interpretation remains based on the combined evidence from ensemble docking, 3D-QSAR, and the complete interaction profiles with each PI3K isoform.

Negative MM/PBSA binding energy estimates were obtained for compound **D23** with all four PI3K isoforms. The most favorable value was observed for PI3Kα (−47.37 ± 4.40 kcal mol^−1^), followed by PI3Kβ (−44.84 ± 5.31 kcal mol^−1^), PI3Kδ (−43.32 ± 4.18 kcal mol^−1^), and PI3Kγ (−43.01 ± 4.02 kcal mol^−1^). Thus, **D23** showed an energetic preference of approximately 2.5–4.4 kcal mol^−1^ for the α isoform. This preference was primarily driven by the noticeably more favorable van der Waals and total non-polar contributions for PI3Kα (−48.81 and −54.65 kcal mol^−1^, respectively). Conversely, its favorable electrostatic interactions were opposed by the largest polar solvation penalty, resulting in an unfavorable net polar contribution of 7.28 kcal mol^−1^. Although these results support the predicted preference of **D23** for PI3Kα, the overlap of the snapshot standard deviations indicates that the differences should be interpreted as a relative energetic trend rather than definitive evidence of statistically significant isoform selectivity.

The duration of the MD simulations represents a limitation of the present study. Although the stabilized trajectory segments enabled assessment of local active site flexibility and ligand-binding behavior, these simulations may not capture slower, large-scale conformational transitions of PI3K. Furthermore, because only the p110 catalytic subunits were simulated, conformational coupling with p85 and regulatory–catalytic subunit rearrangements could not be evaluated. The reported conformations and stability patterns should therefore be interpreted as representative of the conformational space sampled under the applied conditions.

## 3. Materials and Methods

**Figure 21:**
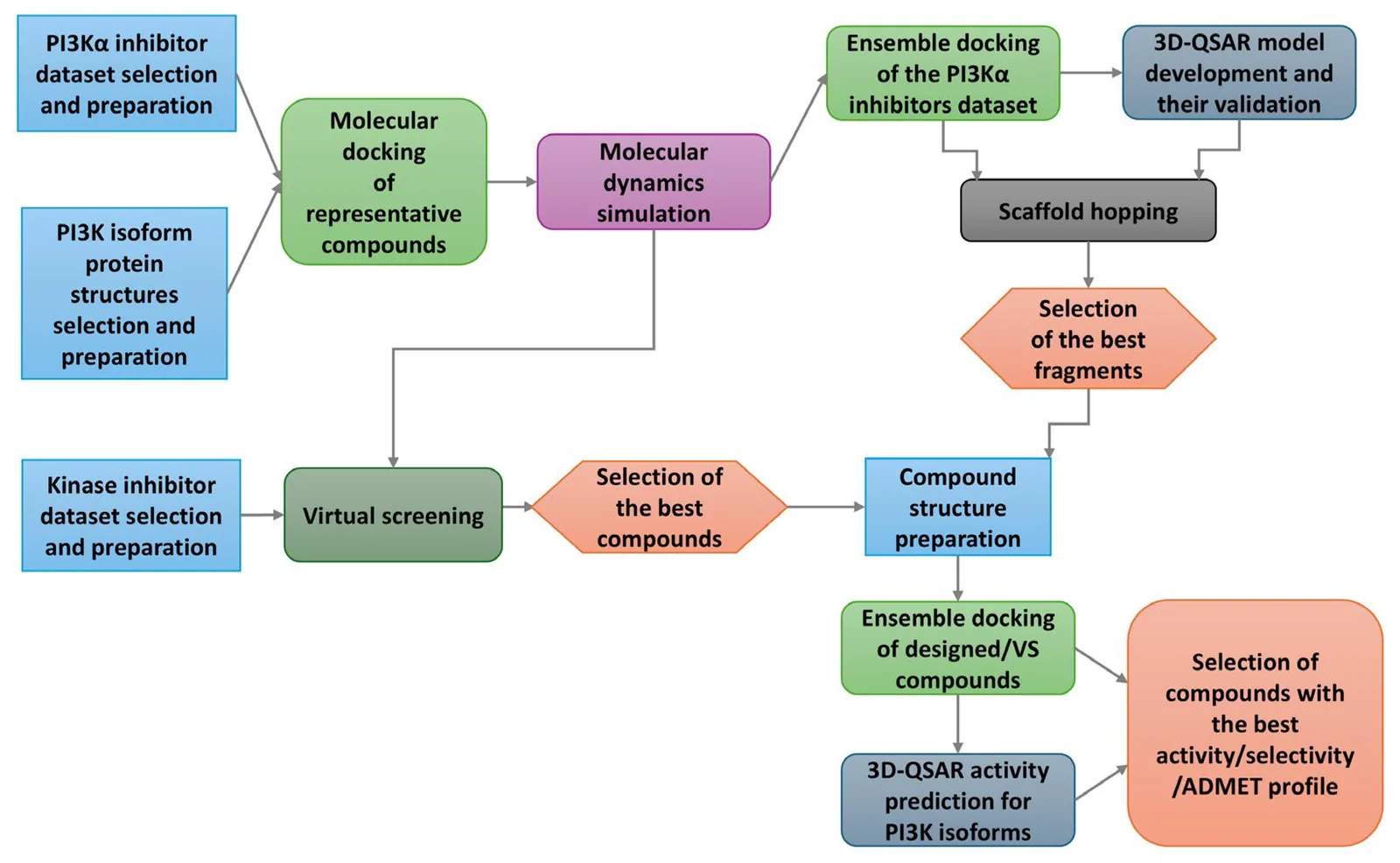
Schematic representation of the CADD protocol used in the study.

A protocol incorporating multiple methods was used to investigate structural characteristics relevant to selective PI3Kα inhibition. As shown in Figure 21, the protocol includes techniques such as molecular dynamics simulations, ensemble docking, 3D-QSAR, scaffold hopping, virtual screening, and ADMET characterization.

### 3.1. Dataset Preparation

**Figure 22:**
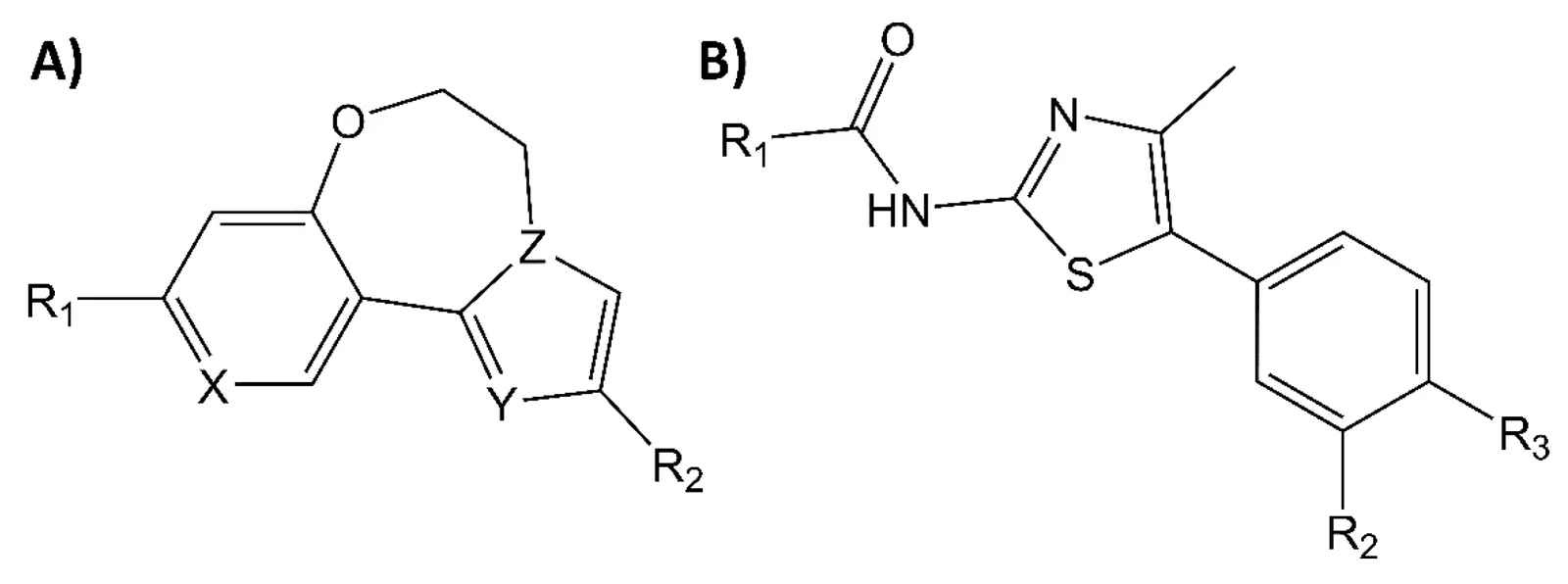
General structural formulas of scaffolds representing (**A**) the Bzo cluster and (**B**) the Thz cluster of PI3Kα inhibitors.

In this study, 37 PI3Kα inhibitors with their activities on each isoform expressed as the negative logarithm of inhibition constant (p*K*_i_ = −log*K*_i_) were obtained from the ChEMBL database (www.ebi.ac.uk/chembl/; accessed on 4 August 2026). The dataset includes benzoxazepine tricyclic derivatives (Bzo cluster), including taselisib (CHEMBL2387080) [26] and thiazole [27] derivatives (Thz cluster) (Figure 22). The p*K*_i_ value ranges were 5.67–10.52 for the PI3Kα isoform, 5.70–8.32 for the PI3Kβ isoform, 5.24–9.19 for the PI3Kγ isoform, and 5.44–9.92 for the PI3Kδ isoform. The general structural formulas of both clusters are represented in Figure 4, and the structures of each compound with their p*K*_i_ values are shown in Tables S14 and S15. The MarvinSketch 24.3.2 program (ChemAxon Kft., Budapest, Hungary; http://www.chemaxon.com; accessed on 4 August 2026) was utilized to select the dominant forms of the compounds under a physiological pH of 7.4. Additionally, chosen forms underwent energy minimization using the PM3 method from Gaussian 09W, Revision A.02 (Gaussian, Inc., Wallingford, CT, USA, 2009) software [34]. The resulting structures were retained as the ligand input structures for all subsequent molecular docking analyses.

### 3.2. Protein Structure Preparation

The X-ray structures of the PI3Kα, PI3Kγ, and PI3Kδ isoforms were obtained from the Protein Data Bank database (https://www.rcsb.org/; accessed on 4 August 2026) (PDB IDs: 5DXH, 6FH5, and 5DXU, respectively), and the p110 subunits were extracted. Since the X-ray structure of the human PI3Kβ isoform is not available, we constructed a homology model of the p110 subunit based on the mouse PI3Kβ X-ray structure (PDB ID: 2Y3A). The NCBI BLAST server (National Center for Biotechnology Information, Bethesda, MD, USA; https://blast.ncbi.nlm.nih.gov/; accessed on 4 August 2026) was used to check the sequence alignment between the human and mouse p110 subunits of PI3Kβ to justify using this template for the homology modeling.

The homology model of the human p110β catalytic subunit and the addition of non-terminal missing residues in the experimental PI3K structures were performed using MODELLER 10.1 (University of California, San Francisco, CA, USA) [35] through the Chimera 1.17.3 interface (Resource for Biocomputing, Visualization, and Informatics, University of California, San Francisco, CA, USA) [36]. One residue adjacent to missing regions was allowed to move by default. The DOPE-HR loop modeling protocol was used, and 1000 models were generated. The model with the lowest zDOPE score was selected for further preparation. The PI3Kβ homology model was evaluated using ROCHECK (EMBL-EBI, Hinxton, UK; https://www.ebi.ac.uk/thornton-srv/software/PROCHECK/; accessed on 4 August 2026), ERRAT through the SAVES v6.1 server (University of California, Los Angeles, CA, USA; https://saves.mbi.ucla.edu/; accessed on 4 August 2026), and ProSA-web (Center of Applied Molecular Engineering, University of Salzburg, Salzburg, Austria; https://prosa.services.came.sbg.ac.at/; accessed on 4 August 2026). PI3Kα, PI3Kγ, and PI3Kδ X-ray structures and the PI3Kβ homology model were protonated at physiological pH 7.4 using the PlayMolecule web server (Acellera, Barcelona, Spain; https://playmolecule.com/; accessed on 4 August 2026).

Initial docking was performed to generate starting complexes for conformational ensemble generation. The most active benzoxazepine and thiazole derivatives in the dataset, CHEMBL3770993 and CHEMBL2071338, respectively, were used as representative ligands and are referred to as **B1** and **T1**. Their previously prepared, PM3-minimized dominant forms at pH 7.4, as described in the Dataset Preparation Subsection, were used as ligand inputs for the initial docking calculations. These compounds were docked into PI3Kα, PI3Kβ, PI3Kγ, and PI3Kδ using GOLD 2023.3 (Cambridge Crystallographic Data Centre, Cambridge, UK) [37]. For each PI3K isoform, the binding site was defined from the crystallographic ligand and comprised all protein atoms located within 6.0 Å of any ligand atom. No user-defined pharmacophoric, hydrogen bond, or distance constraints were applied during the docking procedure. The ChemPLP scoring function was used for the pose ranking. The final binding poses were selected as the highest-scoring ChemPLP solution among those that reproduced the binding orientation and key interactions observed for the experimental crystallographic ligand. The docking procedure was validated by re-docking the crystallographic ligands and calculating heavy-atom RMSD values relative to the reference poses.

MD simulations were performed for the PI3Kα-B1, PI3Kα-T1, PI3Kβ-B1, PI3Kβ-T1, PI3Kγ-B1, PI3Kγ-T1, PI3Kδ-B1, and PI3Kδ-T1 complexes. The simulations were used for local relaxation of protein–ligand complexes and generation of representative receptor conformations for ensemble docking. System preparation was performed using CHARMM-GUI web server [38,39]. Each complex was solvated in a rectangular box of TIP3P water molecules with an 8 Å edge distance. Sodium and chloride ions were added by the Monte Carlo method to neutralize the systems and obtain a final salt concentration of 0.15 M. The CHARMM36m force field [39,40] was used for protein topology and parameters, while ligand parameters were generated using CGenFF [41]. Before production simulations, each system was minimized for 10,000 steps. NVT equilibration was performed for 250 ps, during which the temperature was increased to 310.15 K. Production simulations were then carried out for 50 ns under NPT conditions using NAMD 2.14 (Theoretical and Computational Biophysics Group, University of Illinois Urbana–Champaign, Urbana, IL, USA) [42]. Periodic boundary conditions and the particle mesh Ewald method were applied for long-range electrostatic interactions. Langevin temperature coupling with a friction coefficient of 1 ps−1 was used to control the temperature, while the Langevin piston method was applied for pressure control. The timestep was 2 fs, and trajectory frames were saved every 10 ps.

The trajectories were inspected using backbone RMSD, active site RMSD, ligand RMSD, Cα RMSF, and radius of gyration. These analyses were used only to confirm that the selected trajectory segments were suitable for extracting representative conformations for ensemble docking.

Conformational clustering was performed on the locally stabilized segment of each trajectory using the Ensemble Cluster tool implemented in Chimera, which is based on the method described by Kelley et al. [43]. Pairwise best-fit RMSD values were calculated after superposition of every analyzed trajectory frame onto every other frame using the selected atoms of the predefined active site residues, resulting in an all-against-all RMSD distance matrix. The resulting matrix was subjected to average-linkage hierarchical clustering. The final cluster partition was determined automatically by minimizing a penalty function that balances the number of clusters against the normalized average structural spread within the clusters; therefore, no manually selected RMSD cut-off was applied.

For each cluster, Chimera identified the trajectory frame closest to the cluster centroid as the representative structure. The representative frames from the five most populated clusters were selected for subsequent ensemble docking and interaction analysis. These representative frames are referred to as cluster centroids throughout the manuscript, although they correspond to actual trajectory conformations rather than coordinate-averaged structures. These conformations were used as receptor ensembles in the subsequent docking workflow.

The representative designed compound selected after completion of the design workflow, **D23**, was additionally subjected to 100 ns MD simulations with PI3Kα, PI3Kβ, PI3Kγ, and PI3Kδ using the same general protocol described above. Trajectory frames were saved every 100 ps, giving 1000 frames per system. These simulations were not used for conformational clustering, ensemble docking generation, model development, or compound ranking.

MM/PBSA binding energy calculations were performed using the CaFE 1.0 plugin [44] in VMD (Theoretical and Computational Biophysics Group, University of Illinois Urbana–Champaign, Urbana, IL, USA; https://www.ks.uiuc.edu/Research/vmd/; accessed on 4 August 2026) according to the single-trajectory approach. For the **D23** complexes with the PI3Kα, PI3Kβ, PI3Kγ, and PI3Kδ isoforms, snapshots corresponding to the final 10 ns of each 100 ns trajectory were analyzed. Molecular mechanics contributions were calculated using NAMD 2.14 software [42], whereas polar solvation energies were determined using APBS 3.4.1 software [45], with internal and external dielectric constants of 2 and 80, respectively [46]. The non-polar solvation contribution was estimated from the solvent-accessible surface area. The conformational entropy contribution was not included; therefore, the reported values represent MM/PBSA binding energy estimates rather than absolute binding free energies.

### 3.3. Ensemble Docking

The ensemble docking procedure was performed after the MD simulations to obtain protein–ligand complexes of all compounds in the dataset with all PI3K isoforms. Extracted centroids of the five most numerous clusters (Table S1) were used for the ensemble docking analysis of the dataset compounds. More specifically, five conformations of complexes from the PI3Kα-B1, PI3Kβ-B1, PI3Kγ-B1, and PI3Kδ-B1 simulations (αBzo1-5, βBzo1-5, γBzo1-5, and δBzo1-5) and were used to dock the Bzo cluster compounds (**B1**–**B19**), and five conformations of complexes from the PI3Kα-T1, PI3Kβ-T1, PI3Kγ-T1, and PI3Kδ-T1 simulations (αThz1-5, βThz1-5, γThz1-5, and δThz1-5) were used to dock the Thz cluster compounds (**T1**–**T18**). The same prepared ligand structures described in the Dataset Preparation Subsection were used for ensemble docking. GOLD 2023.3 software [37] was used for this procedure as well. The five centroid structures obtained from each MD simulation were superposed onto the centroid of the most populated cluster using the predefined active site residues. As in the earlier docking procedure, amino acid residues were kept rigid, no constraints were introduced, and the binding pocket was defined in the same manner—defined from the corresponding B1- or T1-derived reference pose and comprised all protein atoms located within 6.0 Å of any reference ligand atom. The same spatial binding site definition was applied to the five aligned conformations within each receptor ensemble. The predicted binding poses were ranked using the ChemPLP scoring function, as previously applied. For each receptor ensemble, the final pose was selected as the highest-scoring ChemPLP solution consistent with the binding orientation and key interactions of **B1** or **T1** in the corresponding MD-derived reference complex. The ensemble docking procedure was validated as well by calculating the heavy-atom RMSD values of the reference ligands after the docking procedure—**B1** in PI3Kα-B1, PI3Kβ-B1, PI3Kγ-B1, and PI3Kδ-B1 protein conformations and **T1** in PI3Kα-T1, PI3Kβ-T1, PI3Kγ-T1, and PI3Kδ-T1 protein conformations. The docked conformations with the highest score and corresponding to the reference structure pose were selected for interaction analysis and further 3D-QSAR studies. The docking results were visualized in Discovery Studio Visualizer 2024 (Dassault Systèmes BIOVIA, San Diego, CA, USA) [47].

### 3.4. 3D-QSAR Study

We conducted 3D quantitative structure and activity relationship (3D-QSAR) studies using the Pentacle 1.07 program (Molecular Discovery Ltd., London, UK) [48]. The models were built to determine structural features that influence the activity of each PI3K isoform. This program utilizes grid-independent descriptors (GRINDs) derived from molecular interaction fields (MIFs), which describe the values of interaction energy between the ligand structure and a probe group, calculated at each node of the grid surrounding the ligand [49]. The energy function used to compute MIFs is calculated by the following equation:

(1) $$E=E_{VDW}+E_{EL}+E_{HB}+S$$

where $E_{VDW}$ is the Van der Waals (VdW) energy, $E_{EL}$ represents electrostatic energy, $E_{HB}$ characterizes hydrogen bond energy, and the parameter S refers to entropy [50]. Each GRIND variable describes a pair of grid nodes associated with specific MIF types and located within a defined distance range. To represent steric and hydrophobic interactions, hydrogen bond acceptors, and hydrogen bond donors, we used the TIP probe (to describe steric interaction), DRY probe (to describe hydrophobic interaction), O probe (carbonyl oxygen interacting with a hydrogen bond donor), and N1 probe (amide nitrogen interacting with a hydrogen bond acceptor), respectively. We utilized a grid step size of 0.5 Å to sample the box enclosing the molecules. MIF discretization was performed to separate the most important regions that represent favorable interactions between the probe and the ligand using the AMANDA algorithm [51]. We used the CLACC (Consistently Large Auto and Cross Correlation) methodology for MIF encoding [52]. It extracts the most consistent variables within a series of structurally related molecules using auto- and cross-correlation between nodes. This methodology enabled the formation of node couples in the same region of the space for all compounds in the examined series, allowing simpler interpretation of the model compared to the MACC (Maximum Auto- and Cross-Correlation) method. The smoothing window for discretizing distances was set to the default 0.8, and the probe cut-off values were held at the default value as well. Combined AMANDA and CLACC algorithms allow for obtaining a newer generation of GRIND descriptors (GRIND-2). Four 3D-QSAR models for each isoform were created using the PLS (Partial Least Squares) regression method. The descriptors were used as independent variables, and in vitro p*K*_i_ activities on PI3Kα, PI3Kβ, PI3Kγ, and PI3Kδ were used as the dependent variables for each 3D-QSAR model. We used the GOLPE-FFD (Generating Optimal Linear PLS Estimations-Fractional Factorial Design) [53] to reduce the number of variables, selecting only relevant variables, and obtain PLS models with the highest prediction ability. The process was repeated until no further improvement in the statistical parameters *R*^2^ (the coefficient of determination) and *Q*^2^ (the cross-validated correlation coefficient) could be made.

All 37 compounds were used for the development of models for PI3Kα, PI3Kγ, and PI3Kδ activity prediction, and 34 compounds were used for the PI3Kβ model due to the absence of defined Ki values for the PI3Kβ isoform for three compounds of the dataset (**T3**, **T8**, and **T13**). The datasets were split into 2/3, forming the training set, and 1/3, forming the test set. The test set was selected to cover the p*Ki* value range homogeneously and to comprise both clusters. Training set compounds were used to build the PLS models, and their predicted experimental and predicted values were used for internal validation, while the test set compounds were used for external validation. Multiple models were formed for the same datasets, and the ones with the highest *Q*^2^ were selected as the final models. PI3Kα, PI3Kγ, and PI3Kδ comprised 25 compounds, and PI3Kβ comprised 23 compounds.

### 3.5. 3D-QSAR Model Validation

Various statistical metrics are used to assess the predictivity of the model, including *R*^2^ for the training set, *Q*^2^, and the root mean square error of estimation (RMSEE) [54]. The following equations are used to calculate *Q*^2^ and RMSEE:

(2) $$Q^{2}\;=\;1\;-\;\frac{\sum {\left(Y_{obs(training)}-Y_{pred(cv-training)}\right)}^{2}}{\sum {\left(Y_{obs(training)}-{\bar{Y}}_{training}\right)}^{2}}$$

(3) $$\mathrm{RMSEE}=\sqrt{\frac{\sum {\left(Y_{obs(training)}-Y_{pred(training)}\right)}^{2}}{n_{\mathrm{training}}}}$$

where *Y*_obs(training)_, $Y_{\mathrm{pred}(\mathrm{training})}$ and ${\bar{Y}}_{\mathrm{training}}$ are the experimental, predicted, and average predicted values of p*K*_i_, respectively. A LOO cross-validation technique was used to calculate the *Q*^2^ parameter in which the $Y_{\mathrm{pred}(\mathrm{cv}-\mathrm{training})}$ parameter is obtained from the models made in cross-validation. The $n_{\mathrm{training}}$ value represents the number of training set compounds.

The models were also subjected to external validation analysis [54,55], which evaluates the predictivity of the models by calculating the coefficient of determination for the test set (*R*^2^_pred_) and the root mean square error of prediction (RMSEP):

(4) $$R_{\mathrm{pred}}^{2}\;=\;1\;-\;\frac{\sum {\left(Y_{obs(test)}-Y_{pred(test)}\right)}^{2}}{\sum {\left(Y_{obs(test)}-{\bar{Y}}_{training}\right)}^{2}}$$

(5) $$\mathrm{RMSEP}=\sqrt{\frac{\sum {\left(Y_{obs(test)}-Y_{pred(test)}\right)}^{2}}{n_{\mathrm{test}}}}$$

In the equations above, *Y*_obs(test)_ and *Y*_pred(test)_ are the experimental and predicted values of p*K*_i_ for the test set and ${\bar{Y}}_{\mathrm{training}}$ indicates mean activity value of the training set. The $n_{\mathrm{test}}$ value represents the number of set compounds.

For further evaluation of the predictive abilities, *r*-metric parameters (*r*^2^_m_, *r*^/2^_m_ and Δ*r*^2^_m_) [56,57] were calculated with the following equations:

(6) $$r_{m}^{2}\;=\;r^{2}\left(1\;-\;\sqrt{\left|r^{2}\;-r_{0}^{2}\right|}\right)$$

(7) $$r_{m}^{/2}=r^{2}\left(1-\sqrt{\left|r^{2}-r_{0}^{/2}\right|}\right)$$

where *r*^2^ and *r*^2^_0_ are squared correlation coefficients between the observed and predicted activities of the test set with and without an intercept, respectively.

The Concordance correlation coefficient (CCC) [58] was also calculated to check the model reliability according to the following equation:

(8) $${\bar{\rho }}_{c}=\frac{2\sum_{i=1}^{n}\;\left(X_{\mathrm{obs}(\mathrm{test})}\;-\;{\bar{X}}_{\mathrm{obs}(\mathrm{test})}\right)\left(Y_{\mathrm{pred}(\mathrm{test})}\;-\;{\bar{Y}}_{\mathrm{pred}(\mathrm{test})}\right)}{\sum_{i=1}^{n}\;{\left(X_{\mathrm{obs}(\mathrm{test})}\;-\;{\bar{X}}_{\mathrm{obs}(\mathrm{test})}\right)}^{2}+\sum_{i=1}^{n}\;{\left(Y_{\mathrm{pred}(\mathrm{test})}\;-\;{\bar{Y}}_{\mathrm{pred}(\mathrm{test})}\right)}^{2}+n_{\mathrm{test}}{\left({\bar{X}}_{\mathrm{pred}(\mathrm{test})}\;-\;{\bar{Y}}_{\mathrm{pred}(\mathrm{test})}\right)}^{2}}$$

In the above equation, *Y*_obs(test)_ and *Y*_pred(test)_ are the experimental and predicted values of p*K*_i_ for the test set, ${\bar{Y}}_{\mathrm{pred}(\mathrm{test})}$ indicates mean activity value of the test set and $n_{\mathrm{test}}$ represents the number of test set compounds.

### 3.6. Applicability Domain

For the QSAR model to be applied accurately, it is essential to establish its applicability domain (AD) [59]. The AD is a theoretical region in chemical space determined by the structures of compounds in the training set [60]. PASW Statistics for Windows, version 18.0 (SPSS Inc., Chicago, IL, USA) [61] was used to perform the leverage approach. It determines if a structure’s activity can be reliably predicted by comparing its leverage value *h* to the critical value h*. The critical value can be calculated using the following equation:

(9) $$h^{*}=\frac{3(p+1)}{n}$$

where *p* represents the number of model variables and *n* represents the number of structures used to create the model.

### 3.7. Design of New Potentially Selective PI3Kα Inhibitors

The results of MD simulations, molecular docking, and 3D-QSAR studies for PI3Kα, PI3Kβ, PI3Kγ, and PI3Kδ isoforms identified key structural features essential for selective PI3Kα inhibition. Based on these findings, the scaffold hopping and R-group replacement method was utilized to modify the structures of lead compounds and design new selective PI3Kα inhibitors. Scaffold hopping enables the identification of potentially active compounds by finding new moieties that can comprise a new inhibitor with similar or enhanced activity and pharmacokinetic profiles. It can also help to overcome challenges like resistance or existing patent limitations [62].

**Figure 23:**
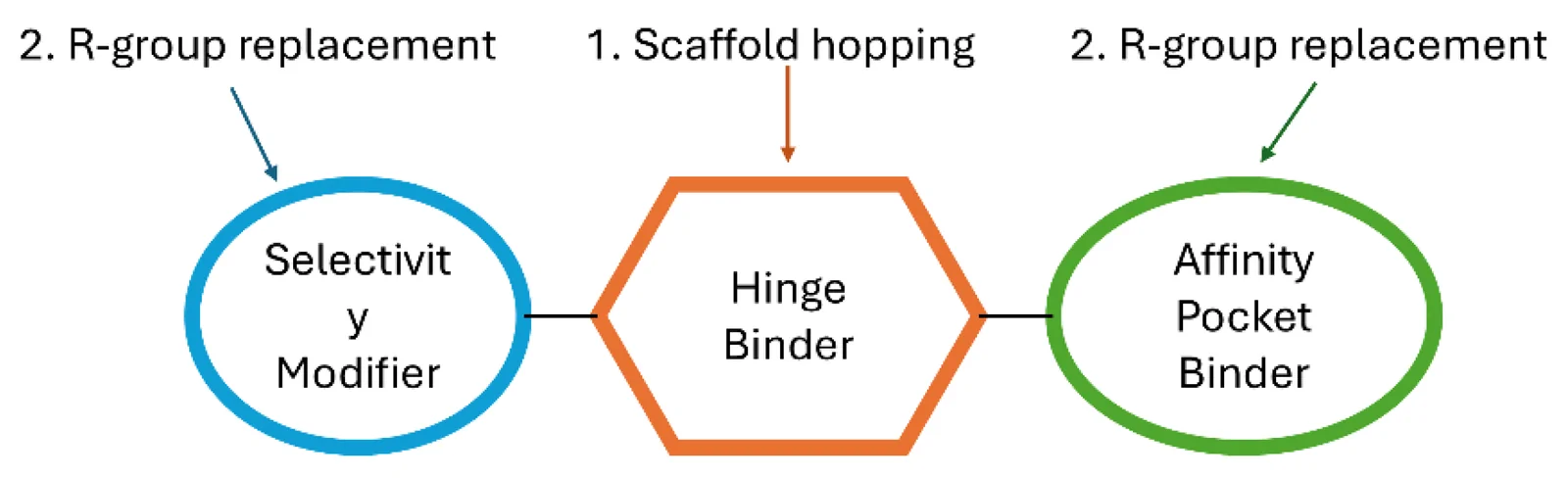
Schematic representation of PI3Kα inhibitors and their substructures, highlighting structural regions used for scaffold hopping and subsequent R-group replacement.

The search for new scaffolds was performed in Cresset’s Spark 10.7.1 software (Cresset Biomolecular Discovery Ltd., Litlington, Cambridgeshire, UK; https://cresset-group.com/software/spark; accessed on 4 August 2026), which uses the XED (the eXtended Electron Distribution) 3 force field and a ligand in a defined 3D conformation as a template [63]. The XED force field captures anisotropic electron density and polarization effects, especially in heteroatoms and bond regions, by describing the electrostatic field via “field points” representing positive, negative, steric, and hydrophobic regions around each molecule [64]. The XED force field was utilized to generate electrostatic fields and field point patterns for the template. A fragment of the molecule (including attachment points with the rest of the structure) was then selected as the bioisosteric fragment replacement site. The protein structure was also imported as an “excluded volume” to check if the replacement fragments clashed with it and ensure that discovered bioisosteres fit into the active site. The template structure of PI3Kα was separated into three fragments: the central fragment—”hinge binder”—and two adjacent R groups—“affinity pocket binder” and “selectivity modifier” (Figure 23).

ChEMBL (https://www.ebi.ac.uk/chembl/; accessed on 4 August 2026), SureChEMBL (https://www.surechembl.org/; accessed on 4 August 2026), and COD (Crystallography Open Database, https://www.crystallography.net/cod/; accessed on 4 August 2026), implemented in Spark with 3D fragments, were searched for replacements with the selected scaffolds. The tested scaffold was then reattached to the unaltered section of the template molecule. The resulting molecule was minimized using the XED force field and analyzed with field point descriptors. The similarity of the new molecule to the original template was assessed based on shape and electrostatic properties. Each new molecule underwent minimization using the XED force field, and field point descriptors were applied for alignment, scoring, and ranking.

The ranking of molecules was performed by a combined score based on the XED force field. Scoring was performed by comparing the new molecule to the template structure. It accounted for the Field Score (electrostatic and steric field similarity) and the Shape Score (shape similarity) after applying penalties for failing to meet field/pharmacophore constraints or clashing with the excluded volume.

The designed compounds were then prepared the same way as the dataset compounds—by finding the major protomer at physiological pH and performing energy minimization with the semiempirical PM3 method. The ensemble docking study was performed to inspect interactions with the active sites of four isoforms and obtain their bioactive conformations. The conformations were used to predict their activities in previously developed 3D-QSAR models.

### 3.8. DFT-Based Quantum Chemical Characterization of Selected PI3Kα Inhibitors

A density functional theory (DFT) study was performed on selected PI3K inhibitors, which were geometrically optimized at the HF/6-31G(d,p) level of theory in the gas phase [65] using Gaussian 09 (Gaussian 09, Revision A.02, Gaussian, Inc., Wallingford, CT, USA, 2016) [34]. Frequency calculations were performed to confirm that all optimized structures correspond to true minima without imaginary frequencies. Single-point energy calculations were subsequently performed at the B3LYP/6-31G(d,p) level to obtain frontier molecular orbital energies, namely, the Highest Occupied Molecular Orbital Energy (E_HOMO_) and the Lowest Unoccupied Molecular Orbital Energy (E_LUMO_), [66] and electrostatic potential (ESP) distributions [30]. The spatial distributions of the HOMO and LUMO orbitals were visualized using Chem3D Ultra 7.0 (CambridgeSoft Corporation, 100 CambridgePark Drive, Cambridge, MA 02140 USA) [67]. Based on the calculated E_HOMO_ and E_LUMO_ values, quantum chemical reactivity descriptors [56,57,58], including chemical potential (*μ*), electronegativity (*χ*), chemical hardness (*η*), softness (*S*), and the electrophilicity index (*ω*), were derived [31,32,68]. The ESP was mapped onto the electron density surface (0.001 a.u.), and the corresponding grid data were generated from the wavefunction for quantitative analysis. Quantitative analysis of the ESP, including determination of molecular electrostatic potential minima (MEP_min_) and maxima (MEP_max_), was performed using the Multiwfn program [69,70], while for the visualization of the ESP maps, Jmol (Jmol development team, 2016; https://jmol.sourceforge.net/; accessed on 4 August 2026) was used [71].

### 3.9. Structure-Based Virtual Screening

To further explore the chemical space of possible α-selective PI3K inhibitors, a structure-based virtual screening (SBVS) was performed. SBVS was performed in FLAP 2.2.2 (Fingerprints for Ligands and Proteins) software (Molecular Discovery Ltd., London, UK) [72,73]. A dataset of known PI3Kα inhibitors was obtained from the ChEMBL database. A stricter threshold of IC_50_ < 100 nM [74,75] was used to find the most promising potent PI3Kα inhibitors. The initial set comprised 387 compounds. The dataset used here was distinct from that utilized for the 3D-QSAR and molecular docking analysis. It primarily consisted of compounds with quinazoline, triazine, and benzimidazole scaffolds, often featuring sulfonamide-linked heteroaryl groups. For the examined compounds, protomers were determined at the physiological pH value of 7.4, after which their 3D structures were generated and optimized using OpenBabel 3.1 [76]. Dominant tautomers were generated using the AMBIT tautomer generator [77] in the KNIME 5.0 environment (KNIME AG, Zurich, Switzerland) [78].

For inactive compounds, the DUDE-Z server (https://tldr.docking.org/start/dudez/; accessed on 4 August 2026) was used to create decoys based on active compound structures. A total of 50 structures were generated per active compound. The DUDE-Z server considers protomers at physiological pH and the 3D conformation of the input structure. It also prefilters input with MW ≥ 500 and logP ≤ 5 [79]. The final database was created using FLAP software. Duplicates were also removed. Up to 25 minimized conformers, with a minimum RMSD distance between two, were retained for each ligand.

Finally, the SBVS database contained 318 active molecules and 2178 decoys.

#### Linear Discriminant Analysis—SBVS Modeling

The linear discriminant analysis (LDA) methodology implemented in FLAP allows the selection of several template protein conformations from provided input structures to account for PI3Kα enzyme flexibility. It finds the top-performing combination of templates and statistical scores that allows better distinction between active and inactive compounds [80]. FLAP describes small molecules and protein binding sites through quadruplet pharmacophoric fingerprints extracted from the MIFs calculated on a grid. Four basic GRID probes were used: H—describes the shape of the compound/binding pocket, N1—describes hydrogen bond donor capabilities, O—describes hydrogen bond acceptor capabilities, and DRY—describes hydrophobic affinities at 0.75 Å resolution. Ten centroid structures of PI3Kα, previously identified in the molecular dynamics study from both PI3Kα-B1 and PI3Kα-T1 simulations (αBzo1-5 and αThz1-5), were imported to generate an LDA-SBVS model. A combination of 3 templates and 2 scores was used for the LDA model generation. A total of 100 active molecules and 400 inactive molecules were used to train the LDA model. Afterwards, AUC (the area under the receiver operating characteristic (ROC) curve) was constructed to estimate the SBVS model’s performance. The ROC enrichment factors, EF 1%, EF 2%, and EF 5%, which measure the area under the curve at 1%, 2%, and 5% false positives, were also evaluated to assess the model’s performance. Afterwards, the Enamine kinase inhibitors library (https://enamine.net/; accessed on 4 August 2026), which includes 64,960 compounds, was used to search for potential PI3Kα active compounds.

The 10 highest-ranking compounds were subsequently subjected to ensemble docking and 3D-QSAR activity prediction for PI3Kα, PI3Kβ, PI3Kγ, and PI3Kδ isoforms, following the same procedure as for the dataset and the compounds from the design study.

### 3.10. Physicochemical and ADMET Characteristics Prediction

The dataset, along with the newly designed structures and selected compounds from SBVS, was evaluated using ADMET Predictor v12.0 software (Simulations Plus, Inc., Lancaster, CA, USA) [81] and the SwissADME server (Molecular Modelling Group, University of Lausanne and SIB Swiss Institute of Bioinformatics, Lausanne, Switzerland; http://www.swissadme.ch/; accessed on 4 August 2026) [82]. These programs allow in silico physicochemical, pharmacokinetic, and toxicology profiling of the tested structures. This analysis aims to identify the most promising drug candidates by ensuring they do not possess unfavorable pharmacokinetic or toxicity properties.

Additionally, our previously established quantitative structure–property relationship (QSPR) model, specifically developed for kinase inhibitors, was employed to predict passive permeability across the blood–brain barrier (BBB) and assess the potential of these compounds to penetrate the central nervous system [33]. This support vector regression (SVR) model generates a log*P*_e_ value, which is a quantitative parameter derived from the in vitro parallel artificial membrane permeability assay (PAMPA). The compounds were assessed to determine whether they fit the PAMPA-SVM model’s AD to ensure the validity of the predictions.

### 3.11. Chemical Space Coverage and Structural Novelty Analysis

Principal component analysis (PCA) was performed as an exploratory unsupervised method to visualize the distribution of known PI3Kα inhibitors, designed compounds and virtual screening hits within the chemical space defined by the calculated molecular descriptors using previously obtained GRIND-2 descriptors. The first two principal components were used to generate the score plot.

To further evaluate the chemical space coverage of the designed compounds and top-ranked virtual screening hits, self-organizing map (SOM) analysis was performed as a complementary unsupervised visualization method [83,84]. The main SOM analysis was carried out using a balanced dataset of 156 compounds, including the Bzo and Thz modeling dataset compounds, a representative subset of 60 highly active ChEMBL PI3Kα inhibitors, designed compounds obtained by scaffold hopping and R-group replacement, and the 30 highest-ranked LDA-SBVS hits. Molecular structures were represented by MACCS structural fingerprints generated from canonical SMILES format using RDKit 2024.03.6 [85]. SOM analysis was performed in Python 3.12.10 (Python Software Foundation, Wilmington, DE, USA) using the MiniSom 2.3.6 library, and each compound was assigned to its best matching unit on the trained SOM grid [86]. Occupancy maps were generated to compare the distribution of known PI3Kα inhibitors with the expanded chemical space obtained after inclusion of the designed and virtually screened compounds. To further assess whether the observed distribution was dependent on the selected ChEMBL subset, the SOM analysis was repeated using the full set of available ChEMBL PI3Kα actives used in the LDA-SVBS model creation.

## 4. Conclusions

The design of selective PI3Kα inhibitors represents a promising strategy for targeted anticancer therapy. In this study, an integrated structure- and ligand-based CADD workflow identified the principal structural requirements for PI3Kα activity and isoform selectivity. These included appropriate hinge binding, hydrophobic and steric complementarity within the affinity pocket, and favorable positioning of the selectivity-modifying substructure toward the isoform-variable region surrounding αGln859.

Six novel hinge-binding candidates spanning five scaffold classes were identified, each showing at least a 10-fold predicted preference for PI3Kα. Their subsequent combination with selected affinity pocket binders and selectivity modifiers resulted in 23 designed compounds with favorable predicted PI3Kα activity and isoform selectivity. Structure-based virtual screening further expanded the investigated chemical space and identified additional scaffolds capable of maintaining key hinge and isoform-variable interactions. DFT, chemical space, and ADMET analyses supported the prioritization of **D4**, **D5**, **D23**, and **VS1** as the most promising candidates, based on their combined predicted activity, isoform selectivity, structural novelty, and drug-like properties.

A representative post-design MD simulation of **D23** supported retention of the predicted PI3Kα binding mode, including stable hinge region binding and favorable positioning within hydrophobic region II. Overall, the results demonstrate the value of integrating complementary computational methods for the design and prioritization of potentially selective PI3Kα inhibitors.

Overall, this study highlights the effectiveness of combining structure-based modeling, 3D-QSAR, scaffold hopping, virtual screening, DFT analysis, chemical space evaluation, and ADMET profiling in the discovery and prioritization of selective PI3Kα inhibitors. The results provide a solid foundation for future synthesis, in vitro evaluation, and further optimization of the proposed compounds as potential candidates for PI3Kα-targeted anticancer therapy.

Possible next steps in this research could include the synthesis and analytical characterization of **D4**, **D5**, **D23**, and **VS1**, followed by in vitro IC_50_ testing against PI3Kα, PI3Kβ, PI3Kγ, and PI3Kδ to evaluate their predicted potency and isoform selectivity. Where feasible, co-crystallographic studies or refinement using additional experimental structural data could further assess the proposed binding modes and support subsequent compound optimization.

## Figures and Tables

**Table 1:** RMSD values of re-docked reference ligands in ensemble docking.

Isoform	RMSD (Å)	
	Bzo Cluster	Thz Cluster
PI3Kα	0.997	1.019
PI3Kβ	1.671	1.254
PI3Kγ	1.549	1.104
PI3Kδ	0.859	0.878

**Table 2:** 3D-QSAR model validation for the PI3Kα, PI3Kβ, PI3Kγ, and PI3Kδ isoforms.

	*R* ^2^	*Q* ^2^	RMSEE	*R* ^2^_pred_	RMSEP	*r* ^2^_m_	*r* ^/2^_m_	Δ*r*^2^_m_	(*r*^2^_m_ + *r*^/2^_m_)/2	(*r*^2^_m_ − *r*^/2^_m_)/*r*^2^_m_	*k*′	CCC
PI3Kα	0.93	0.82	0.38	0.73	0.50	0.63	0.76	0.13	0.70	0.00	0.98	0.88
PI3Kβ	0.94	0.82	0.21	0.71	0.38	0.65	0.69	0.04	0.67	0.01	0.98	0.86
PI3Kγ	0.95	0.77	0.26	0.75	0.30	0.63	0.80	0.17	0.70	0.00	1.01	0.89
PI3Kδ	0.96	0.88	0.27	0.71	0.48	0.66	0.74	0.08	0.70	0.01	1.02	0.87
Limit	>0.7	>0.5		>0.5		>0.5	>0.5	<0.2	>0.5	<0.1	0.85 < *k*′ < 1.15	>0.85

**Table 3:** Molecular electrostatic potential extrema (kcal/mol) and quantum chemical reactivity descriptors (eV) of investigated compounds.

Compound	MEP_min_	MEP_max_	E_HOMO_	E_LUMO_	∆E Gap	μ	χ	η	S	ω
**B1**	−46.19	51.15	−4.950	−0.312	4.638	−2.631	2.631	2.319	0.216	1.493
**B19**	−47.55	34.69	−5.358	−0.777	4.580	−3.067	3.067	2.290	0.218	2.054
**T1**	−48.52	44.70	−5.960	−1.170	4.790	−3.565	3.565	2.395	0.209	2.653
**T18**	−51.43	66.42	−6.293	−1.682	4.611	−3.987	3.987	2.306	0.217	3.447
**D1**	−47.28	48.83	−4.784	−0.790	3.993	−2.787	2.787	1.997	0.250	1.945
**D4**	−47.68	50.86	−4.984	−1.374	3.610	−3.179	3.179	1.805	0.277	2.799
**D13**	−50.14	47.28	−5.320	−1.186	4.134	−3.253	3.253	2.067	0.242	2.560
**D19**	−49.66	53.06	−6.567	−2.052	4.515	−4.310	4.310	2.258	0.221	4.113
**D21**	−45.06	50.68	−5.688	−1.507	4.181	−3.598	3.598	2.091	0.239	3.096
**D23**	−59.27	58.91	−7.610	−2.558	5.052	−5.084	5.084	2.526	0.198	5.116

**Table 4:** Predicted p*K*_i_ values, selected PI3K centroids, and key binding site interactions for the most promising compounds for further optimization.

Compound	Predicted p*K*_i_ Values				Binding Site Interactions			
	PI3Kα	PI3Kβ	PI3Kγ	PI3Kδ	PI3Kα	PI3Kβ	PI3Kγ	PI3Kδ
**B1** (Lead)	10.523	8.253	8.697	9.654	αBzo2: Ile800, Ile848, **Val851**, **Ser854**, **Gln859**, Met922	βBzo4: **Lys775**, **Trp787**, Glu852, Val853, **Val854**	γBzo3: Tyr867, Ile879, Glu880, Ile881, **Val882**, Met953	δBzo1: Trp760, Asp787, **Lys779**, Ile825, **Val828**, Met900, Ile910
**D4**	9.527	6.921	7.123	7.554	αBzo2: Ile800, **Tyr836**, Ile848, **Val851**, **Ser854**, **Gln859**, Met922	βBzo3: **Lys775**, **Trp787**, Glu852, Val853, **Val854**, Met926	γBzo2: Trp812, Ile831, **Lys833**, **Tyr867**, Glu880, Ile881, **Val882**, **Met953**, Ile963	δBzo1: Trp760, Asp787, **Tyr813**, Ile825, **Val828**, Ser831, Asp832, **Thr833**, Met900, Ile910
**D5**	9.980	7.026	7.530	8.075	αBzo2: Met772, Ile800, **Tyr836**, Ile848, **Val851**, **Ser854**, **Gln859**, Met922	βBzo3: Trp787, Ile803, Glu852, Val853, **Val854**, Met926	γBzo2: Trp812, Trp812, Ile831, Asp841, **Tyr867**, Ile879, Glu880, Ile881, **Val882**, Ile963	δBzo1: Trp760, **Tyr813**, Ile825, **Val828**, **Thr833**, Met900, Ile910
**D23**	8.621	6.738	5.233	7.011	αBzo2: Met772, Pro778, Ile800, Ile848, Val850, **Val851**, **Ser854**, **Gln859**, Met922	βBzo3: Met779, **Trp787**, Ile803, Glu852, Val853, **Val854**, Met926	γBzo3: Trp812, Ile831, **Tyr867**, Ile879, Glu880, **Val882**, **Ala885**, Ile963	δBzo1: Trp760, **Lys779**, Ile825, **Val828**, Met900, Ile910
**VS1**	8.292	6.160	7.458	6.993	αBzo2: **Gln859**, **Val851**, Val850, Met922, Ile932, Tyr836	βBzo2: **Val854**, Val853, Met926, Ile936	γThz4: **Lys833**, **Val882**, Asp964, Ile831, Ile879, Ile963, Met953	δBzo1: **Val828**, **Asn836**, **Arg830**, Ile910, Met900, Ile825, Trp760, Tyr813

Note: Amino acid residues forming hydrogen bonds are shown in bold.

## Data Availability

The data supporting the findings of this study are available within this article and its Supplementary Materials. The input structures and activity data were obtained from publicly available databases, including the Protein Data Bank and ChEMBL, as described in Section 3. Additional computational data, including raw molecular dynamics trajectories and docking output files, are available from the corresponding authors upon request.

## Supplementary Materials

The following supporting information can be downloaded at https://www.mdpi.com/article/10.3390/molecules31162782/s1: Figure S1: The alignment of human and mouse PI3Kβ enzyme amino acid sequences; Figure S2: Initial docking poses and protein–ligand interactions of the representative benzoxazepine derivative B1 in (A) PI3Kα, (B) PI3Kβ, (C) PI3Kγ, and (D) PI3Kδ; dark green indicates conventional hydrogen bonds, light green indicates carbon–hydrogen bonds, medium green indicates van der Waals interactions, purple indicates T-shaped π–π interactions, pink indicates alkyl and π–alkyl interactions, and orange indicates π–sulfur interactions; Figure S3: Initial docking poses and protein–ligand interactions of the representative thiazole derivative T1 in (A) PI3Kα, (B) PI3Kβ, (C) PI3Kγ, and (D) PI3Kδ; dark green indicates conventional hydrogen bonds, light green indicates carbon–hydrogen bonds, medium green indicates van der Waals interactions, purple indicates T-shaped π–π interactions, pink indicates alkyl and π–alkyl interactions, and orange indicates π–sulfur interactions; Figure S4: Backbone, active-site, and ligand RMSD profiles of PI3Kα–B1, PI3Kα–T1, PI3Kβ–B1, PI3Kβ–T1, PI3Kγ–B1, PI3Kγ–T1, PI3Kδ–B1, and PI3Kδ–T1 complexes during MD simulations; Figure S5: Plots of RMSF values for (A) PI3Kα–B1, (B) PI3Kα–T1, (C) PI3Kβ–B1, (D) PI3Kβ–T1, (E) PI3Kγ–B1, (F) PI3Kγ–T1, (G) PI3Kδ–B1, and (H) PI3Kδ–T1 simulation complexes; Figure S6: Plots of the radius-of-gyration values for (A) PI3Kα–B1, (B) PI3Kα–T1, (C) PI3Kβ–B1, (D) PI3Kβ–T1, (E) PI3Kγ–B1, (F) PI3Kγ–T1, (G) PI3Kδ–B1, and (H) PI3Kδ–T1 simulation complexes; Table S1: Cluster-centroid representative frames with the numbers of frames in the most populated clusters obtained from PI3Kα–B1, PI3Kα–T1, PI3Kβ–B1, PI3Kβ–T1, PI3Kγ–B1, PI3Kγ–T1, PI3Kδ–B1, and PI3Kδ–T1 simulation complexes; Figure S7: Ramachandran plot of the representative PI3Kβ homology model in complex with compound B1; Table S2: Summary data of the Ramachandran plot of the representative PI3Kβ homology model in complex with compound B1; Figure S8: Z-scores of experimental protein structures based on the number of amino acids obtained from the ProSA-web server and the position of the representative PI3Kβ homology model in complex with compound B1; Figure S9: Experimental pK_i_ versus predicted pK_i_ values for the (A) PI3Kα, (B) PI3Kβ, (C) PI3Kγ, and (D) PI3Kδ 3D-QSAR models; Figure S10: Williams plots defining the applicability domains of the PI3Kα, PI3Kβ, PI3Kγ, and PI3Kδ 3D-QSAR models; Table S3: Experimental versus predicted pK_i_ values for the PI3Kα, PI3Kβ, PI3Kγ, and PI3Kδ 3D-QSAR models; Table S4: Summary of the most important variables in the PI3Kα 3D-QSAR model; Table S5: Summary of the most important variables in the PI3Kβ 3D-QSAR model; Table S6: Summary of the most important variables in the PI3Kγ 3D-QSAR model; Table S7: Summary of the most important variables in the PI3Kδ 3D-QSAR model. Figure S11: Structures of designed compounds with the newly selected hinge-binding scaffolds; Table S8: Predicted pK_i_ values for the designed compounds with the newly selected hinge-binding scaffolds; Figure S12: Structures of designed compounds with the new affinity-pocket binders and selectivity modifiers; Table S9: Predicted pK_i_ values, selected PI3K centroids, and key binding site interactions for the designed compounds with the new affinity pocket binders and selectivity modifiers; amino acids forming hydrogen bonds are bolded; Figure S13: Williams plots defining the applicability domains for the designed compounds in the PI3Kα, PI3Kβ, PI3Kγ, and PI3Kδ 3D-QSAR models; Figure S14: Structures of compounds selected using the SBVS procedure; Table S10: Predicted pK_i_ values, selected PI3K centroids, and key binding site interactions for the compounds obtained from the SBVS procedure; amino acids forming hydrogen bonds are bolded; Figure S15: Williams plots defining the applicability domains of compounds obtained using the LDA-SBVS procedure for the PI3Kα, PI3Kβ, PI3Kγ, and PI3Kδ 3D-QSAR models; Table S11: Physicochemical properties and synthetic-accessibility scores of the designed and virtual-screening compounds obtained using the SwissADME server; Table S12: Pharmacokinetic properties of the designed and virtual-screening compounds obtained using ADMET Predictor software; Table S13: logP_e_ values predicted using the PAMPA-SVM model: low-permeability compounds, −6.14 < logP_e_ < −5.66; medium-permeability compounds, −5.66 < logP_e_ < −5.33; and high-permeability compounds, logP_e_ > −5.33; Figure S16: Williams plot defining the applicability domain of the PAMPA-SVM model, including the PAMPA-SVM dataset compounds, lead compound, designed compounds, and compounds obtained using the LDA-SBVS procedure; Figure S17: Self-organizing-map U-matrix projection of the PI3Kα-inhibitor chemical space. Figure S18: Backbone (A), active-site (B), and ligand (C) RMSD profiles of D23 in complex with PI3Kα, PI3Kβ, PI3Kγ, and PI3Kδ during 100 ns MD simulations; Figure S19: Cα RMSF profiles of PI3Kα (A), PI3Kβ (B), PI3Kγ (C), and PI3Kδ (D) in complex with D23 during 100 ns MD simulations; Figure S20: Radius-of-gyration profiles of PI3Kα, PI3Kβ, PI3Kγ, and PI3Kδ in complex with D23 during 100 ns MD simulations; Table S14: Bzo-cluster dataset compounds and their pK_i_ values for the PI3Kα, PI3Kβ, PI3Kγ, and PI3Kδ isoforms; Table S15: Thz-cluster dataset compounds and their pK_i_ values for the PI3Kα, PI3Kβ, PI3Kγ, and PI3Kδ isoforms.
