# Supplementary material for: A Computational Framework for the Design and Development of Isoform Selective PI3Kα Inhibitors as Novel Anticancer Agents
Source: Molecules. 2026 Aug 10;31(16):2782. doi: 10.3390/molecules31162782 (PMC13515537; doi:10.3390/molecules31162782)
Supplement: Supplementary file 1 [file molecules-31-02782-s001.zip › Supplementary material.pdf]

A Computational Framework for the Design and Development of Isoform Selective PI3Kα inhibitors as Novel Anticancer Agents

Milan Jovanović [1,2,*], Teodora Djikic-Stojsic [1], Branislav Stanković [3], Marija Popovic-Nikolic [1] and Katarina Nikolic [1,*]

[1] Department of Pharmaceutical Chemistry, Faculty of Pharmacy, University of Belgrade, Vojvode Stepe 450, 11221 Belgrade, Serbia; teodora.djik88@gmail.com (T.D.-S.); marija.popovic@pharmacy.bg.ac.rs (M.P.-N.)

[2] Department of Molecular Biology and Endocrinology, "VINCA" Institute of Nuclear Sciences—National Institute of the Republic of Serbia, University of Belgrade, Mike Petrovica Alasa 12–14, Vinca, 11351 Belgrade, Serbia

[3] Department of Nuclear and Plasma Physics, "VINCA" Institute of Nuclear Sciences—National Institute of the Republic of Serbia, University of Belgrade, Mike Petrovica Alasa 12–14, Vinca, 11351 Belgrade, Serbia; branislav.stankovic@vin.bg.ac.rs

* Correspondence: milan.jov94@gmail.com (M.J.); katarina.nikolic@pharmacy.bg.ac.rs (K.N.)

Supplementary material

Total number of Figures: 20
Total number of Tables: 15

Supplementary Results S1. PI3Kβ model validation and ensemble-generation trajectory inspection

The BLAST alignment between the human and mouse p110β sequences showed high sequence similarity, supporting the use of the mouse PI3Kβ structure as a template for homology modeling. The selected PI3Kβ model had a zDOPE score of −0.46. The estimated Cα RMSD from the native structure was 1.396 Å, and the estimated fraction of Cα atoms within 3.5 Å of the corresponding native structure atoms after rigid-body superposition was 0.935. Additional validation was performed on the representative PI3Kβ model conformation obtained after MD-based refinement.

The Ramachandran plot analysis showed that the model satisfied standard stereochemical quality criteria. The overall ProSA-web Z-score was −13.68, placing the model within the range expected for experimentally determined protein structures of comparable size. ERRAT analysis gave an overall quality factor of 92.308, with no active-site residues showing error values above the 95% rejection limit. These results supported the suitability of the PI3Kβ model for subsequent structure-based modeling.

Re-docking of crystallographic ligands into the prepared PI3Kα, PI3Kβ, PI3Kγ, and PI3Kδ structures yielded heavy-atom RMSD values of 0.518 Å, 1.049 Å, 0.857 Å, and 0.258 Å, respectively, confirming that the docking setup reproduced the reference binding modes.

The production trajectories of the **B1** and **T1** complexes were inspected using backbone RMSD, active-site RMSD, ligand RMSD, Cα RMSF, and radius of gyration. These analyses were used to identify locally stabilized trajectory segments suitable for the extraction of representative receptor conformations for ensemble docking. Backbone (Figure S4A) and active-site (Figure S4B) RMSD profiles indicated that, after the initial relaxation phase, the complexes reached stable plateaus suitable for structural clustering. Ligand RMSD values were compatible with preservation of the docked binding poses during the analyzed trajectory segments (Figure S4C).

The RMSF profiles showed larger fluctuations in flexible loop regions than in ordered secondary-structure elements, whereas the active-site regions, including the P-loop and hydrophobic region, displayed comparatively limited fluctuations (Figure S5). Radius of gyration profiles indicated preservation of overall protein compactness throughout the analyzed segments (Figure S6).

Based on trajectory inspection, frames from the 30–50 ns interval were clustered using active-site RMSD to generate representative receptor conformations for ensemble docking of the whole dataset. Cluster centroid representative frames (one frame per 10 ps), with the number of frames of the most numerous clusters, are represented in Table S3.

Figure S1. The alignment of human and mouse PI3Kβ enzyme amino acid sequences.

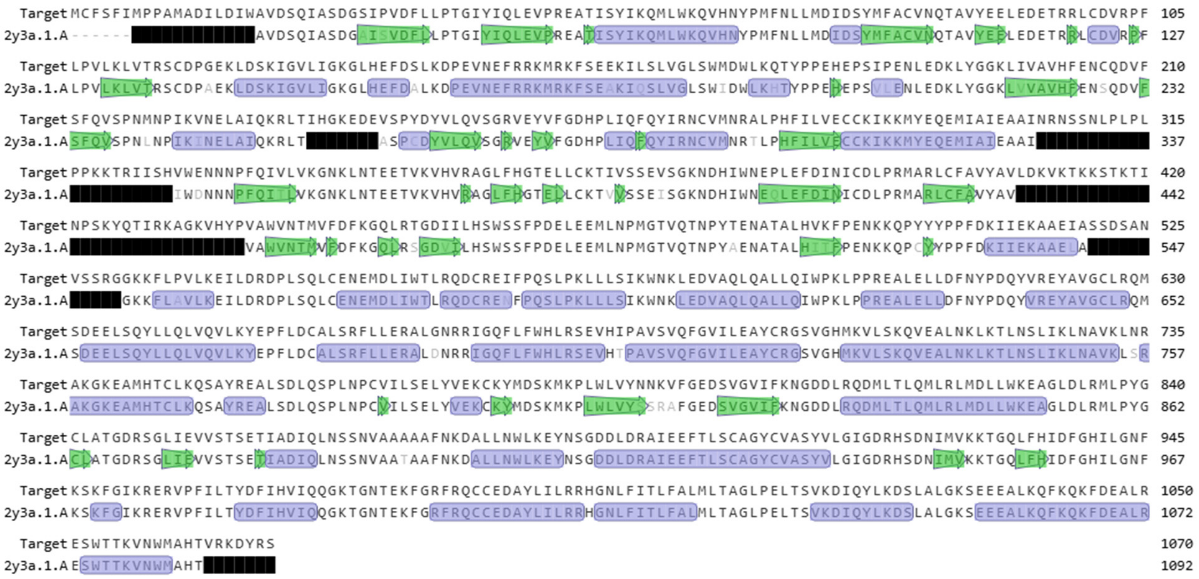

Figure S2. Initial docking poses and protein–ligand interactions of the representative benzoxazepine and thiazole derivatives for B1 in A) PI3Kα, B) PI3Kβ, C) PI3Kγ, and D) PI3Kδ; dark green - conventional hydrogen bonds, light green – carbon hydrogen bonds, medium green van der Waals interactions, purple - T-shaped π-π interactions, pink – alkyl and π-alkyl interactions, orange – π-sulfur interactions.

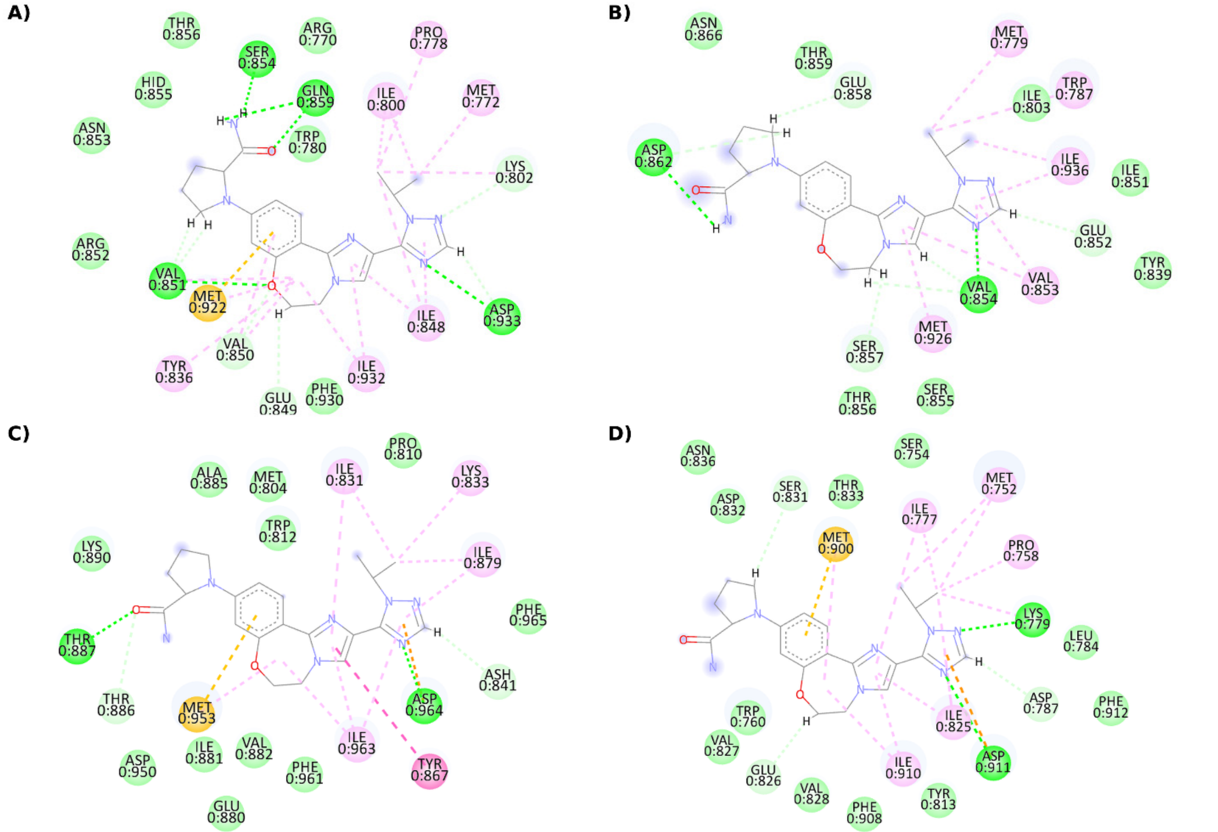

Figure S3. Initial docking poses and protein–ligand interactions of the representative benzoxazepine and thiazole derivatives for T1 in A) PI3Kα, B) PI3Kβ, C) PI3Kγ, and D) PI3Kδ; dark green - conventional hydrogen bonds, light green – carbon hydrogen bonds, medium green van der Waals interactions, purple - T-shaped π-π interactions, pink – alkyl and π-alkyl interactions, orange – π-sulfur interactions.

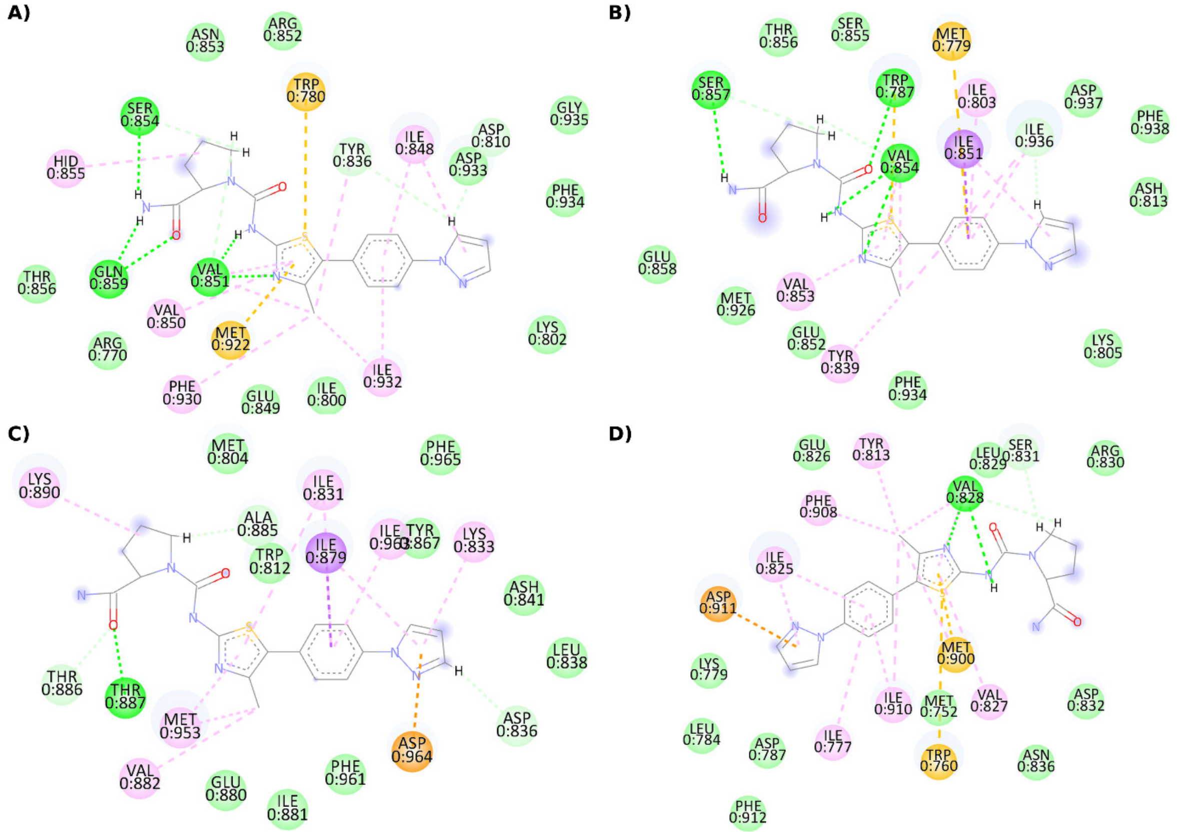

Figure S4. Backbone, active-site, and ligand RMSD profiles of PI3Kα-B1, PI3Kα-T1, PI3Kβ-B1, PI3Kβ-T1, PI3Kγ-B1, PI3Kγ-T1, PI3Kδ-B1, and PI3Kδ-T1 complexes during MD simulations.

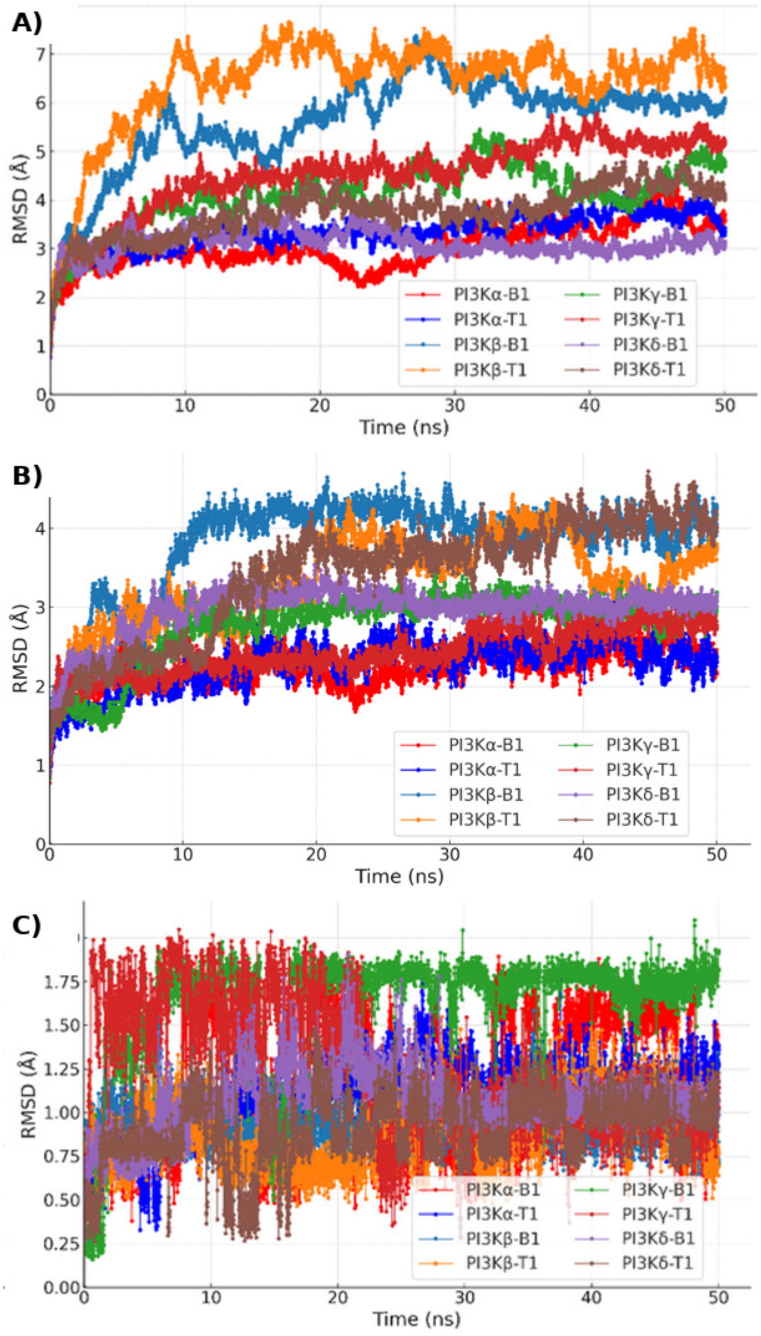

Figure S5. Plots of RMSF values for A) PI3Kα-B1, B) PI3Kα-T1, C) PI3Kβ-B1, D) PI3Kβ-T1, E) PI3Kγ-B1, F) PI3Kγ-T1, G) PI3Kδ-B1, and H) PI3Kδ-T1 simulation complexes.

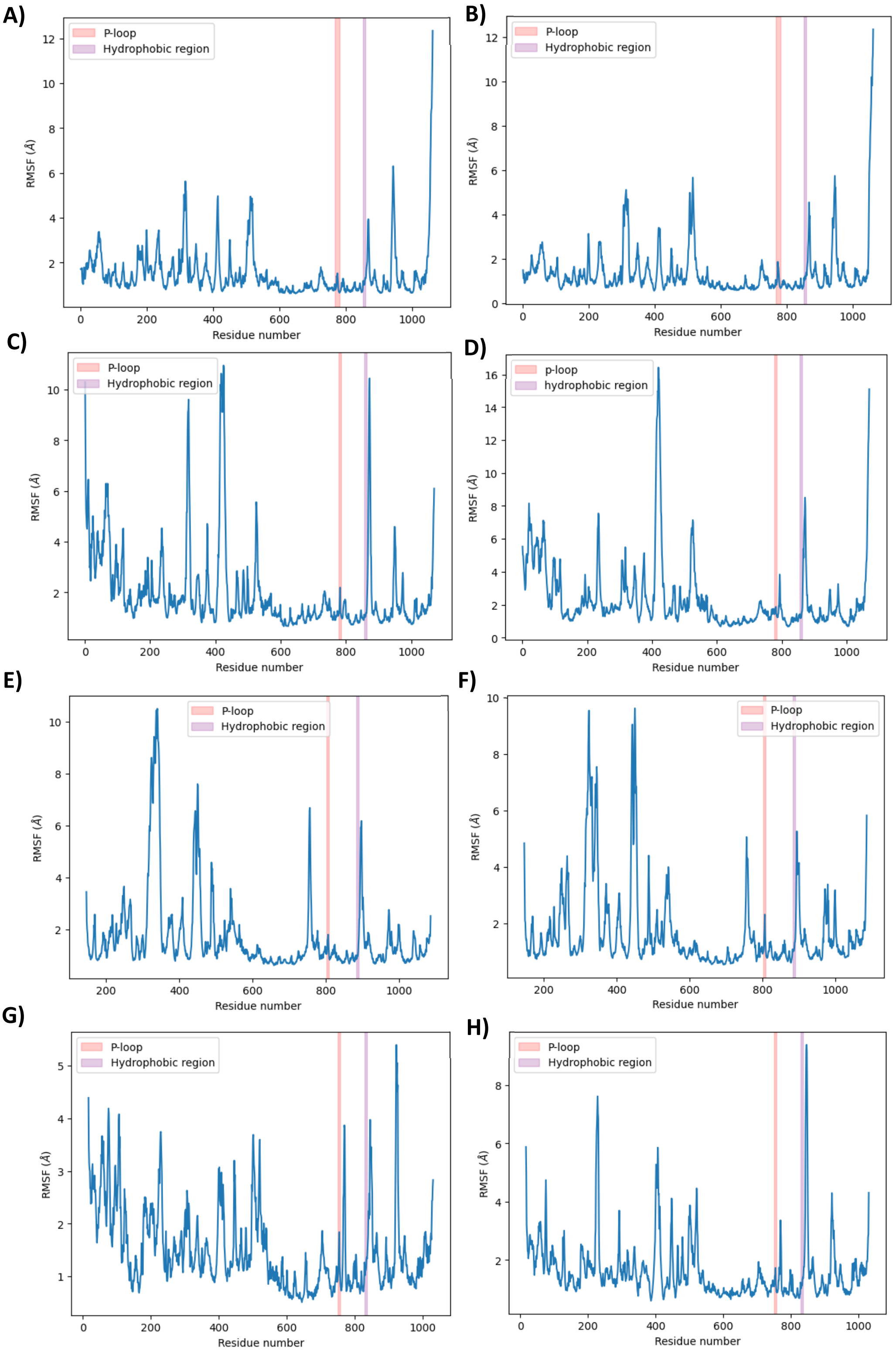

Figure S6. Plots of the radius of gyration values for A) PI3Kα-B1, B) PI3Kα-T1, C) PI3Kβ-B1, D) PI3Kβ-T1, E) PI3Kγ-B1, F) PI3Kγ-T1, G) PI3Kδ-B1, and H) PI3Kδ-T1 simulation complexes.

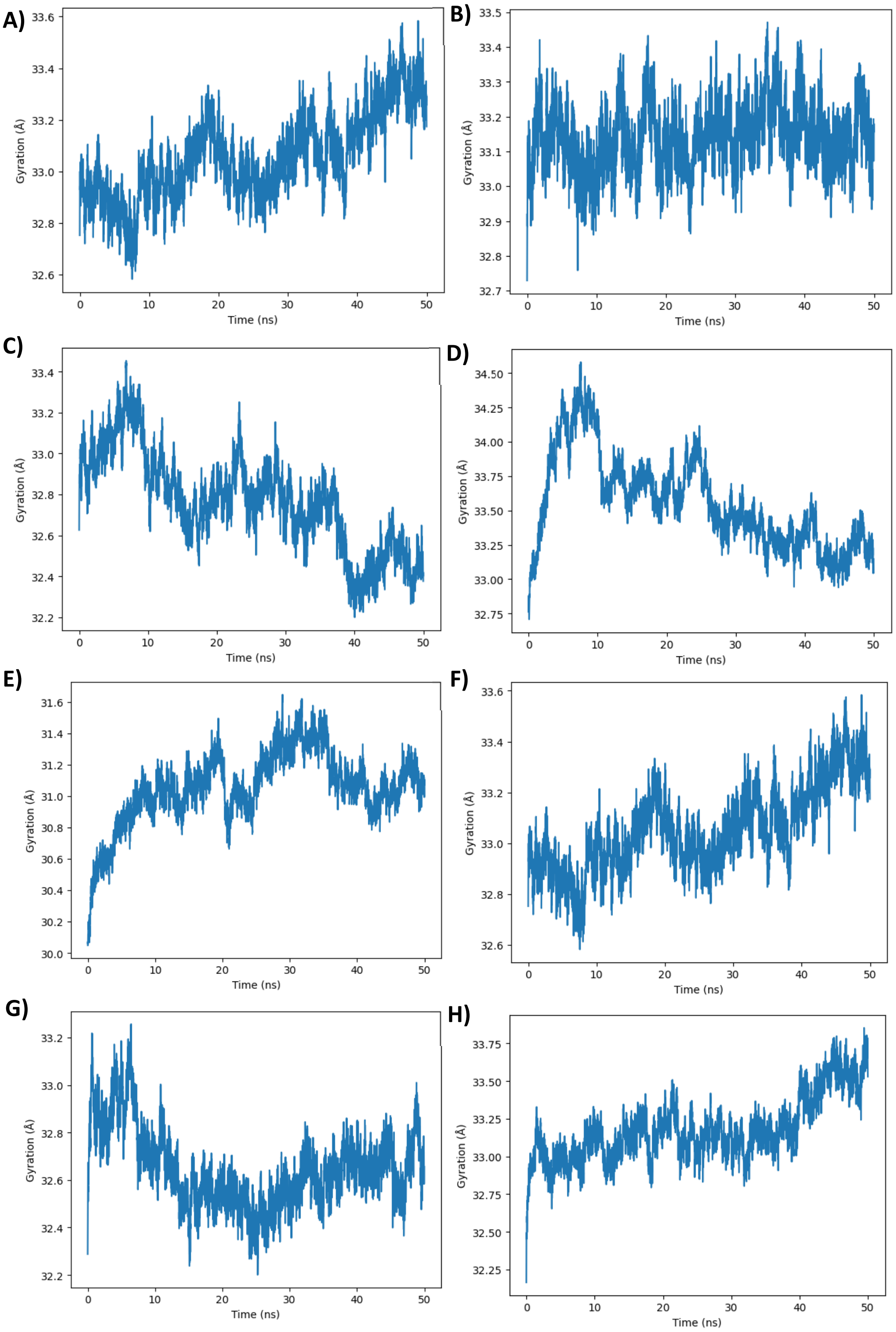

Table S1. Cluster centroid representative frames with the number of frames of the most numerous clusters obtained from PI3Kα-B1, PI3Kα-T1, PI3Kβ-B1, PI3Kβ-T1, PI3Kγ-B1, PI3Kγ-T1, PI3Kδ-B1, and PI3Kδ-T1 simulation complexes.

Isoform	Bzo cluster				Thz cluster			
	Conformation cluster	Centroid name	Centroid MD frame	Cluster members number	Conformation cluster	Centroid name	Centroid MD frame	Cluster members number
PI3Kα	1	αBzo1	3318	52	1	αThz1	3996	50
	2	αBzo2	3072	51	2	αThz2	3445	47
	3	αBzo3	4347	49	3	αThz3	4357	44
	4	αBzo4	3357	46	4	αThz4	3182	44
	5	αBzo5	4278	45	5	αThz5	4246	43
PI3Kβ	1	βBzo1	4233	66	1	βThz1	4357	93
	2	βBzo2	4961	60	2	βThz2	4487	58
	3	βBzo3	3857	53	3	βThz3	3824	54
	4	βBzo4	4141	48	4	βThz4	3195	52
	5	βBzo5	3398	44	5	βThz5	4958	49
PI3Kγ	1	γBzo1	3424	106	1	γThz1	4608	189
	2	γBzo2	4097	75	2	γThz2	3182	62
	3	γBzo3	3555	69	3	γThz3	4168	61
	4	γBzo4	4637	65	4	γThz4	4351	45
	5	γBzo5	3818	60	5	γThz5	3159	44
PI3Kδ	1	δBzo1	3175	151	1	δThz1	4747	103
	2	δBzo2	4143	72	2	δThz2	3187	88
	3	δBzo3	4702	69	3	δThz3	3869	75
	4	δBzo4	4573	59	4	δThz4	4082	65
	5	δBzo5	3274	52	5	δThz5	4887	52

Figure S7. Ramachandran plot of the representative PI3Kβ homology model in complex with compound B1.

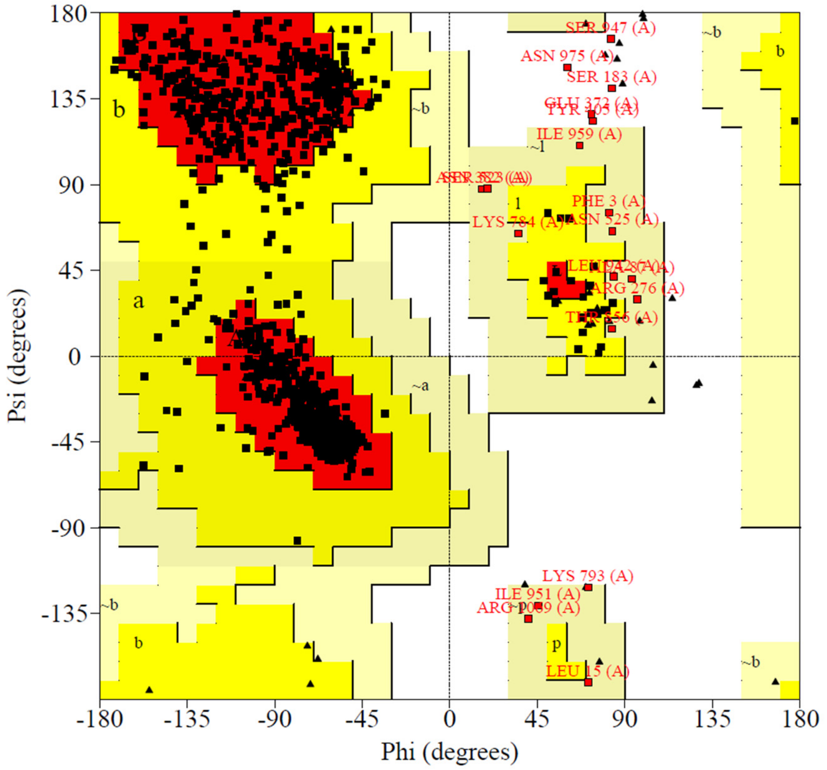

Table S2. Summary data of Ramachandran plot of the representative PI3Kβ homology model in complex with compound B1.

	**No. of residues**	**Percentage**
Most favoured regions [A,B,L]	872	90.2%
Additional allowed regions [a,b,l,p]	76	7.9%
Generously allowed regions [~a,~b,~l,~p]	14	1.4%
Disallowed regions [XX]	5	0.5%
Non-glycine and non-proline residues	967	100.0%
End-residues (excl. Gly and Pro)	3	
Glycine residues	50	
Proline residues	51	
Total number of residues	1071	

Figure S8. Z-scores of experimental protein structures based on the number of amino acids obtained from ProSA-web server, and the position of the representative PI3Kβ homology model in complex with compound B1.

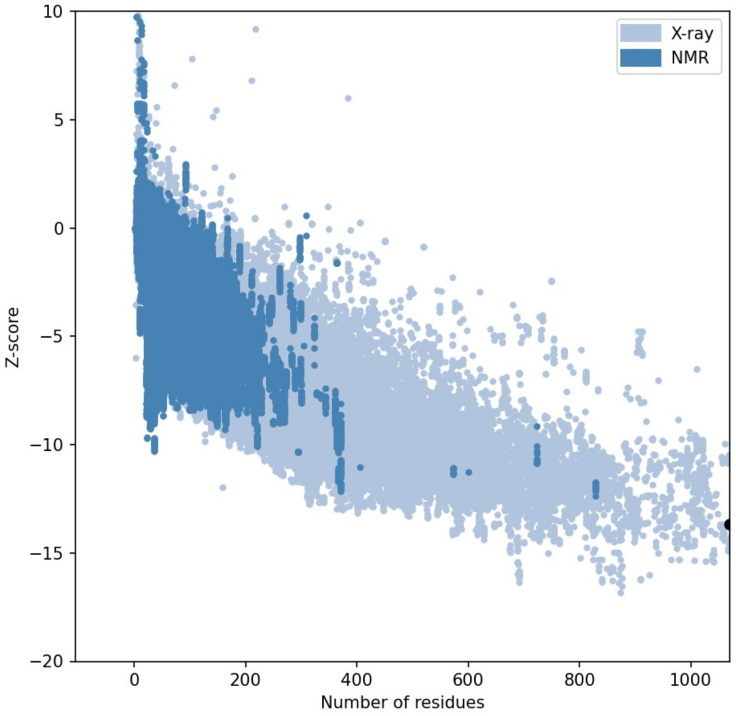

Figure S9. The experimental pK$_i$ vs. predicted pK$_i$ values plot for A) PI3Kα, B) PI3Kβ, C) PI3Kγ, and D) PI3Kδ 3D-QSAR models.

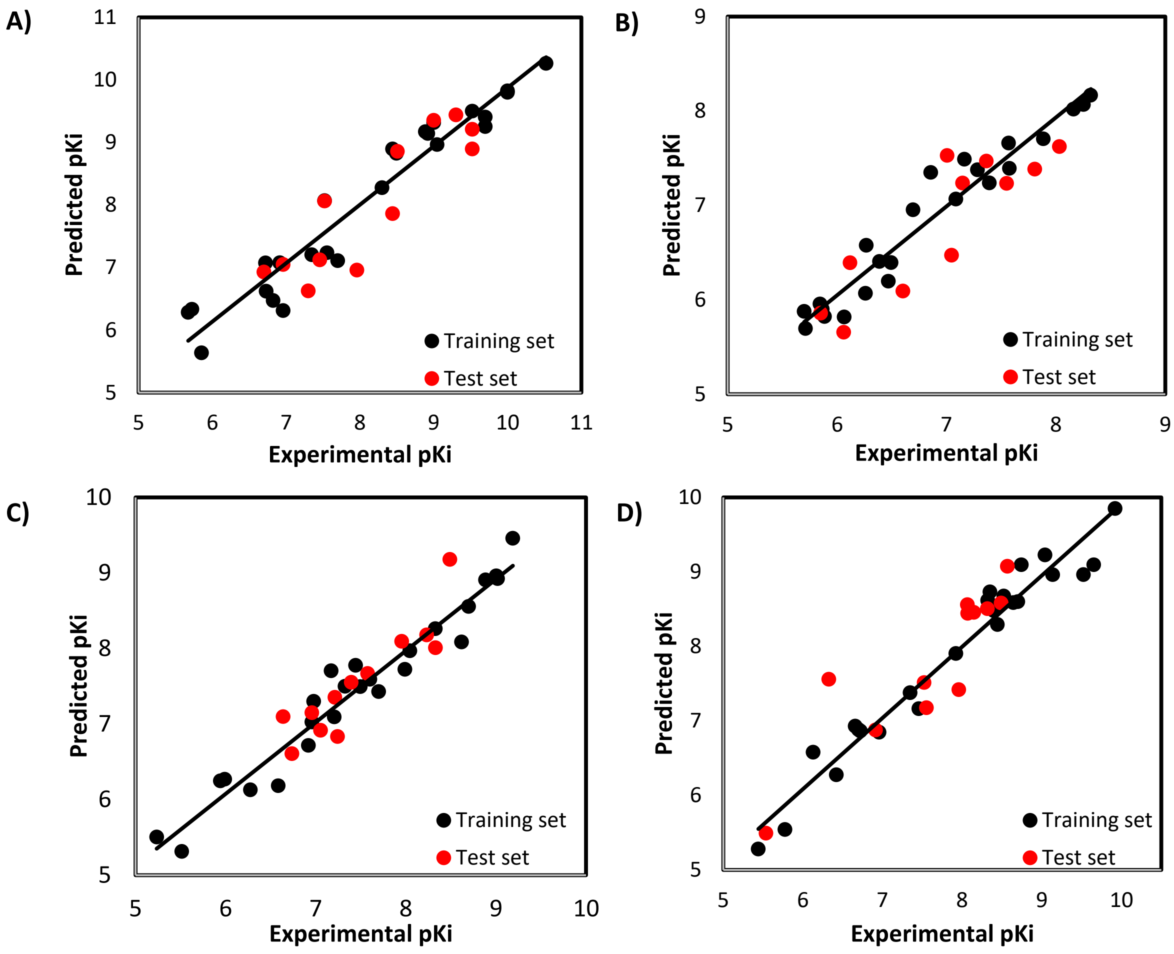

Figure S10. The Williams plots (defined AD domains) of PI3Kα, PI3Kβ, PI3Kγ, and PI3Kδ 3D-QSAR models.

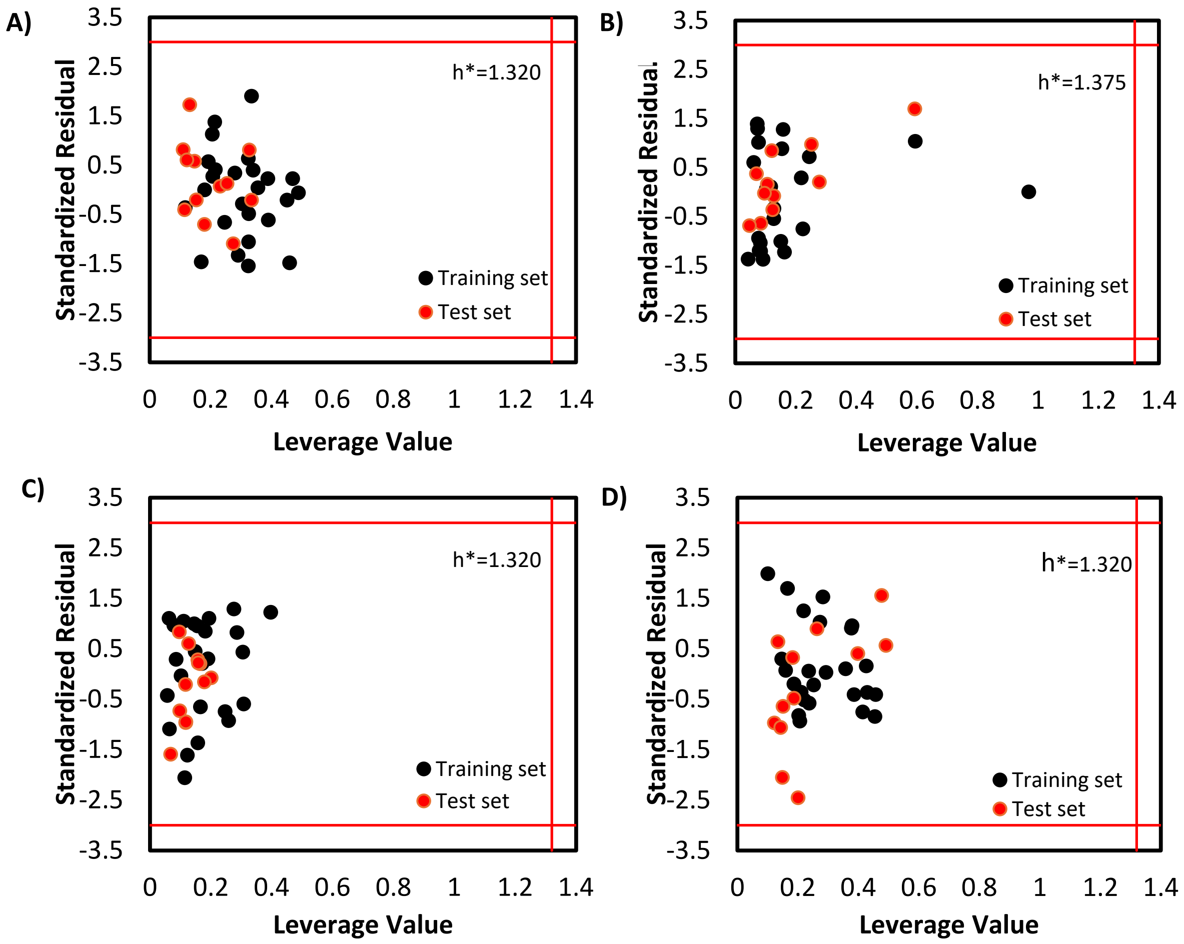

Table S3. The experimental pK_i vs. predicted pK_i values for A) PI3Kα, B) PI3Kβ, C) PI3Kγ, and D) PI3Kδ 3D-QSAR models.

PI3Kα			PI3Kβ			PI3Kγ			PI3Kδ		
Training set	Exp. pK_i	Pred. pKi	Training set	Exp. pK_i	Pred. pKi	Training set	Exp. pK_i	Pred. pK_i	Training set	Exp. pK_i	Pred. pK_i
**B1**	10.523	10.265	**B6**	8.319	8.165	**B14**	9.187	9.459	**B8**	9.921	9.849
**B2**	10.000	9.822	**B1**	8.253	8.066	**B13**	9.018	8.924	**B1**	9.654	9.095
**B3**	10.000	9.800	**B2**	8.161	8.018	**B8**	9.004	8.962	**B6**	9.523	8.963
**B4**	9.699	9.255	**B14**	7.886	7.705	**B11**	8.886	8.907	**B2**	9.137	8.962
**B5**	9.699	9.406	**B5**	7.575	7.392	**B1**	8.697	8.556	**B14**	9.041	9.228
**B8**	9.523	9.500	**B4**	7.569	7.661	**B6**	8.620	8.087	**B11**	8.745	9.096
**B10**	9.046	8.966	**B10**	7.393	7.239	**B2**	8.328	8.261	**B4**	8.699	8.601
**B11**	9.000	9.316	**B11**	7.284	7.377	**B4**	8.046	7.968	**B13**	8.642	8.588
**B13**	8.921	9.141	**B18**	7.165	7.489	**B5**	7.991	7.724	**T10**	8.523	8.676
**B14**	8.886	9.171	**B9**	7.086	7.066	**T18**	7.699	7.430	**B18**	8.444	8.294
**B16**	8.495	8.826	**B12**	6.857	7.349	**B12**	7.602	7.591	**B5**	8.398	8.478
**B18**	8.444	8.894	**B16**	6.696	6.952	**T2**	7.495	7.495	**T12**	8.398	8.492
**T1**	8.301	8.274	**T16**	6.495	6.394	**T16**	7.444	7.776	**B16**	8.349	8.733
**T3**	7.699	7.107	**T12**	6.469	6.197	**B7**	7.327	7.499	**B10**	8.321	8.617
**T4**	7.553	7.235	**T14**	6.387	6.406	**T7**	7.208	7.093	**B19**	7.921	7.906
**B19**	7.523	8.069	**B19**	6.268	6.578	**B16**	7.173	7.706	**T6**	7.456	7.164
**T7**	7.347	7.203	**T2**	6.260	6.069	**B19**	6.979	7.300	**T7**	7.347	7.378
**T10**	6.959	6.311	**T10**	6.066	5.818	**T14**	6.959	7.028	**T9**	6.959	6.847
**T11**	6.914	7.076	**T6**	5.886	5.823	**T10**	6.921	6.716	**T14**	6.721	6.865
**T12**	6.824	6.472	**T15**	5.866	5.902	**T13**	6.585	6.182	**T15**	6.699	6.883
**T13**	6.728	6.620	**T5**	5.845	5.956	**T1**	6.276	6.130	**T1**	6.658	6.931
**T14**	6.721	7.075	**T9**	5.712	5.696	**T12**	5.991	6.268	**T18**	6.420	6.277
**T16**	5.854	5.636	**T1**	5.699	5.878	**T3**	5.943	6.246	**T17**	6.131	6.580
**T17**	5.724	6.331				**T9**	5.514	5.315	**T3**	5.777	5.542
**T18**	5.672	6.282				**T8**	5.236	5.504	**T13**	5.441	5.280
Test set	Exp. pKi	Pred. pKi	Test set	Exp. pKi	Pred. pKi	Test set	Exp. pKi	Pred. pKi	Test set	Exp. pKi	Pred. pKi
**B6**	9.523	8.895	**B8**	8.032	7.623	**B17**	8.489	9.178	**B17**	8.489	8.582
**B7**	9.523	9.210	**B13**	7.807	7.383	**B18**	8.330	8.009	**B12**	8.066	8.558
**B9**	9.301	9.439	**B3**	7.550	7.233	**B10**	8.233	8.180	**B15**	8.147	8.457
**B12**	9.000	9.354	**B17**	7.365	7.469	**B3**	7.955	8.094	**B3**	8.569	9.075
**B15**	8.509	8.857	**B15**	7.147	7.237	**B9**	7.577	7.668	**B7**	8.319	8.506
**B17**	8.444	7.860	**T18**	7.046	6.471	**B15**	7.395	7.554	**B9**	8.071	8.443
**T2**	7.959	6.957	**B7**	7.007	7.528	**T6**	7.244	6.833	**T5**	7.523	7.515
**T5**	7.523	8.063	**T17**	6.602	6.094	**T4**	7.215	7.354	**T8**	5.538	5.493
**T6**	7.456	7.120	**T7**	6.119	6.393	**T17**	7.056	6.916	**T16**	6.328	7.557
**T8**	7.301	6.625	**T4**	6.060	5.656	**T5**	6.959	7.149	**T2**	7.959	7.419
**T9**	6.959	7.047	**T11**	5.851	5.858	**T11**	6.738	6.607	**T11**	6.914	6.878
**T15**	6.699	6.927				**T15**	6.638	7.098	**T4**	7.553	7.176

Table S4. Summary of the most important variables PI3Kα 3D-QSAR model.

Variable	PLS coefficient	Distance (Å)	MIF node pair	Description
var488	0.442	16.0-16.4	O-N1	The most influential positive variable, formed in Bzo cluster, represents an HBD and an HBA
var481	0.321	13.2-13.6	O-N1	Similar to Var488, but present in Thz cluster as well
var284	0.276	11.2-11.6	DRY-O	Favorable distance between a hydrophobic moiety and an HBD
var291	0.252	14.0-14.4	DRY-O	Similar to var284, present in most Bzo cluster compounds, and only in some Thz cluster compounds
var397	0.214	5.2-5.6	DRY-TIP	Present in all compounds, forms at a hydrophobic structure with a steric entity
var15	0.193	6.0-6.4	DRY-DRY	The distance between two hydrophobic moieties, present in all compounds
var170	-0.202	16.8-17.2	N1-N1	Forms in some compounds from both clusters, formed between two HBAs
var271	-0.218	6.0-6.4	DRY-O	The distance between a hydrophobic entity and a HBD, present in almost all compounds
var528	-0.271	6.4-6.8	O-TIP	Unfavorable distance between a HBD and a steric moiety
var79	-0.392	6.0-6.4	O-O	The most influential negative variable, it describes the distance between two HBDs, present only in some Thz cluster compounds

Table S5. Summary of the most important variables PI3Kβ 3D-QSAR model.

Variable	PLS coefficient	Distance (Å)	MIF node pair	Description
var519	0.183	14.4-14.8	O-N1	The variable with the highest PLS coefficient. Present in some compounds from both clusters, representing a distance between a HBD and a HBA on opposite sides of a molecule.
var350	0.161	2-2.4	DRY-N1	Primarily in Bzo cluster derivatives, explaining the distance between a hydrophobic moiety and a HBA in vicinity.
var508	0.158	10-10.4	O-N1	The optimal distance expressed in most Bzo cluster compounds and a few Thz cluster compounds derivatives between a HBD and a HBA
var146	0.169	3.2-3.6	N1-N1	The distance between two HBA, not present in some less active cluster compounds.
var375	0.121	12.4-12.8	DRY-N1	Similar to var350, but present in all compounds
var11	0.140	4.4-4.8	DRY-DRY	The distance between two hydrophobic moieties, present in all compounds
var228	0.115	8.4-8.8	TIP-TIP	The distance between two steric regions, present in all compounds.
var282	-0.123	2.4-2.8	DRY-O	Absent in more active compounds, it measures the distance between a hydrophobic region and a nearby HBD
var291	-0.130	6.0-6.4	DRY-O	Like var 282, but present in almost all compounds.
var566	-0.183	5.6-6	O-TIP	The negative impact of the distance between HBD and a steric moiety in close vicinity. This variable has the greatest negative influence.

Table S6. Summary of the most important variables PI3Kγ 3D-QSAR model.

Variable	PLS coefficient	Distance (Å)	MIF node pair	Description
var23	0.212	9.2-9.6	DRY-DRY	The most impactful positive variable: present in all compounds, it measures the distance between two hydrophobic regions.
var11	0.187	2.8-3.2	DRY-DRY	Distance between two close hydrophobic entities in a region, present in all compounds
var260	0.174	17.6-18.0	TIP-TIP	Present in almost all compounds, between two steric regions, presenting an edge-to-edge distance in compounds.
var474	0.136	16.8-17.2	DRY-TIP	The distance between a steric entity and a hydrophobic region, on opposite sides of each compound.
var523	-0.170	7.6-8.0	O-TIP	Similar to var592; Distance between a HBD and a HBA, not formed in some compounds with the highest activity.
var149	-0.241	2.0-2.4	N1-N1	Unfavorable distance between two HBAs,
var590	-0.279	6.4-6.8	O-TIP	Not present in the most active compound; measures the distance between a HBD and a close steric moiety.
var89	-0.284	6.8-7.2	O-O	Between two HBDs, formed in some compounds of both clusters
var325	-0.288	14.8-15.2	DRY-O	Describes the distance between a HBD and a hydrophobic moiety on the opposite side of the compound, primarily in Bzo cluster compounds.
var623	-0.337	18.8-19.2	O-TIP	Similar to var325; Primarily expressed for less active compounds; describes the distance between a HBD and a steric moiety on the opposite sides of the compounds.

Table S7. Summary of the most important variables PI3Kδ 3D-QSAR model.

Variable	PLS coefficient	Distance (Å)	MIF node pair	Description
var74	-0.250	1.6-2.0	O-O	Proximity of two HBDs, present only in some lower THz cluster compounds, it has the highest negative influence on activity.
var82	-0.225	4.8-5.2	O-O	Similar to var74.
var178	-0.186	15.2-15.6	N1-N1	Present in several compounds of both clusters, depicting the distance between two HBA on the opposite sides of the compound.
var310	0.202	12.0-12.4	DRY-O	The distance between a hydrophobic region and a HBD, formed in most compounds.
var355	0.243	2.0-2.4	DRY-N1	Optimal distance between a hydrophobic region and a HBA.
var461	0.254	16.4-16.8	DRY-TIP	Optimal distance between a hydrophobic region and a steric entity.
var527	0.277	14.8-15.2	O-N1	The variable with the highest positive impact on activity. The distance between a HBD and a HBA, not present in some less active compounds of both cluster
var589	0.201	3.6-4.0	O-TIP	Close distance between a HBA and a steric entity.
var634	-0.188	1.6-2.0	N1-TIP	Short distance between a HBA and a steric entity, expressed in all compounds.
var677	0.246	18.8-19.2	N1-TIP	This variable exhibits the highest negative PLS coefficient value. Distance between a HBA and a steric moiety on the opposite sides of compounds.

Figure S11. Structures of designed compounds with the new chosen hinge binder scaffolds.

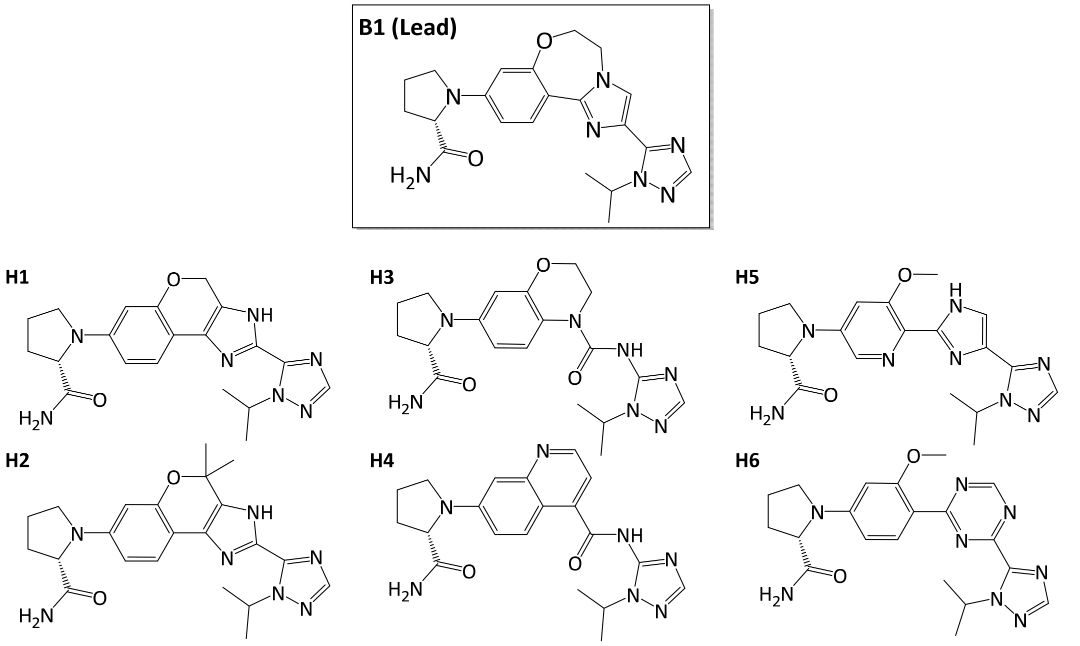

Table S8. Predicted pKi values for the designed compounds with the new chosen hinge binder scaffolds.

Compound	Predicted pK_i values			
	PI3Kα	PI3Kβ	PI3Kγ	PI3Kδ
**B1** (Lead)	10.523	8.253	8.697	9.654
**H1**	10.038	6.984	6.659	8.776
**H2**	9.644	7.045	6.958	7.791
**H3**	9.522	6.235	7.499	8.295
**H4**	9.527	6.921	7.123	7.554
**D5**	9.980	7.026	7.530	8.075

Figure S12. Structures of designed compounds with the new affinity pocket binders and selectivity modifiers.

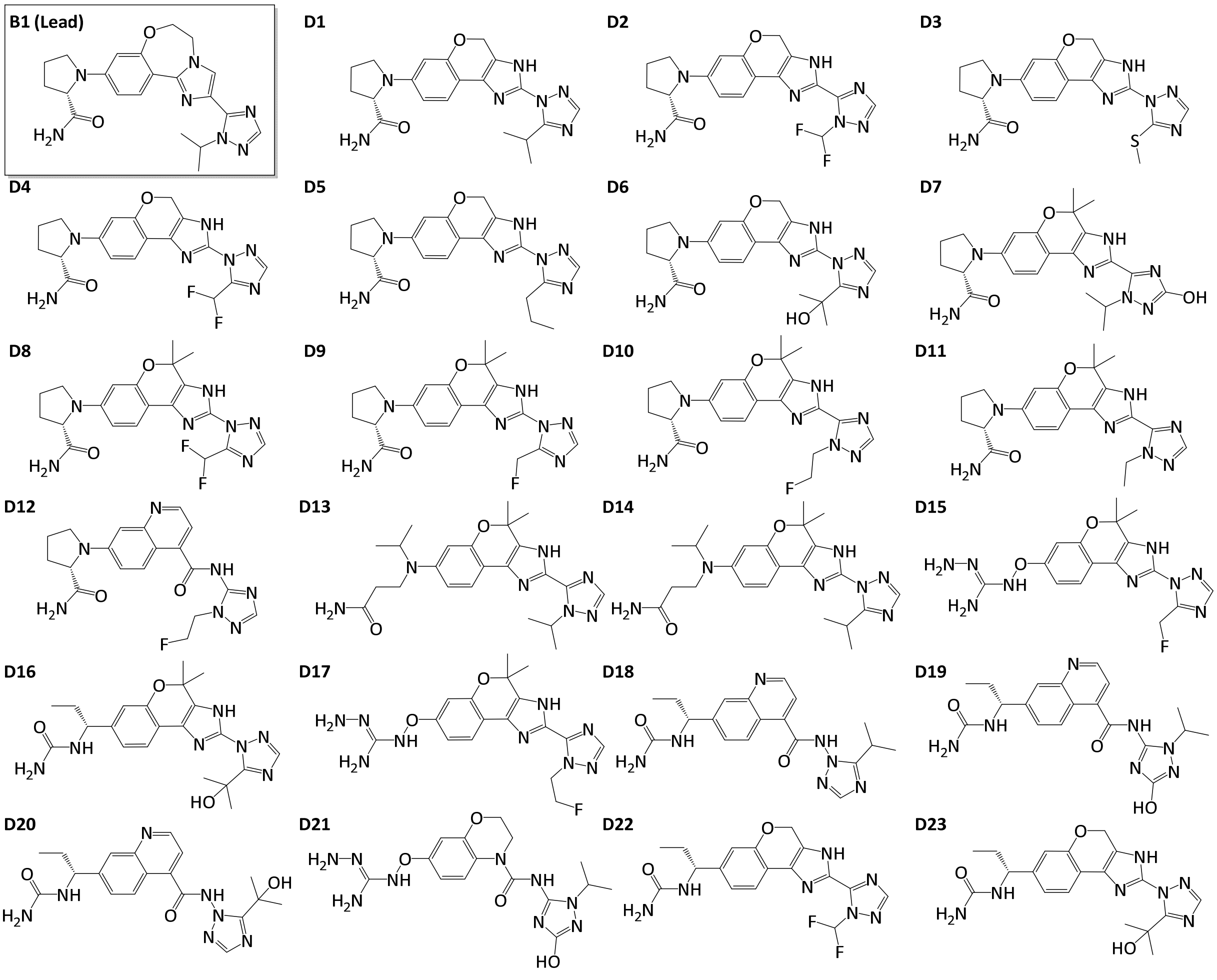

Table S9. Predicted pKi values, selected PI3K centroids, and key binding site interactions for the designed compounds with the new affinity pocket binders and selectivity modifiers; amino acids forming hydrogen bonds are bolded.

Compound	Predicted pK_i values				Binding site interactions			
	PI3Kα	PI3Kβ	PI3Kγ	PI3Kδ	PI3Kα	PI3Kβ	PI3Kγ	PI3Kδ
**B1** (Lead)	10.523	8.253	8.697	9.654	αBzo2: Ile800, Ile848, **Val851**, **Ser854**, **Gln859**, Met922	βBzo4: **Lys775**, **Trp787**, Glu852, Val853, **Val854**	γBzo3: Tyr867, Ile879, Glu880, Ile881, **Val882**, Met953	δBzo1: Trp760, Asp787, **Lys779**, Ile825, **Val828**, Met900, Ile910
**D1**	10.038	6.984	6.659	8.776	αBzo2: Ile800, Ile848, **Val851**, **Ser854**, **Gln859**, Met922	βBzo3: **Lys775**, **Trp787**, Glu852, Val853, **Val854**, Met926	γBzo2: **Tyr867**, Ile879, Glu880, Ile881, **Val882**, Met953	δBzo5: Trp760, Asp787, **Tyr813**, Ile825, **Val828**, Met900, Ile910
**D2**	9.644	7.045	6.958	7.791	αBzo2: Ile800, **Tyr836**, Ile848, **Val851**, **Ser854**, **Gln859**, Met922	βBzo4: **Lys775**, **Trp787**, Ile803, Val853, **Val854**, Met926	γBzo2: Trp812, Ile831, **Lys833**, **Tyr836**, Asp841, Ile879, Glu880, Ile881, **Val882**, Ile963	δBzo1: Trp760, Asp787, **Tyr813**, **Asp823**, Ile825, Val827, **Val828**, **Ser831**, Met900, Ile910
**D3**	9.522	6.235	7.499	8.295	αBzo2: **Tyr836**, Ile848, **Val851**, **Ser854**, **Gln859**, Met922	βBzo4: **Lys775**, **Trp787**, Ile803, Val853, **Val854**, Met926	γBzo2: Trp812, Trp812, Ile831, **Tyr867**, Ile879, **Val882**, Ile963	δBzo1: Trp760, **Tyr813**, Ile825, **Val828**, Ser831, Met900, Ile910
**D4**	9.527	6.921	7.123	7.554	αBzo2: Ile800, **Tyr836**, Ile848, **Val851**, **Ser854**, **Gln859**, Met922	βBzo3: **Lys775**, **Trp787**, Glu852, Val853, **Val854**, Met926	γBzo2: Trp812, Ile831, **Lys833**, **Tyr867**, Glu880, Ile881, **Val882**, **Met953**, Ile963	δBzo1: Trp760, Asp787, **Tyr813**, Ile825, **Val828**, Ser831, Asp832, **Thr833**, Met900, Ile910
**D5**	9.980	7.026	7.530	8.075	αBzo2: Met772, Ile800, **Tyr836**, Ile848, **Val851**, **Ser854**, **Gln859**, Met922	βBzo3: Trp787, Ile803, Glu852, Val853, **Val854**, Met926	γBzo2: Trp812, Trp812, Ile831, Asp841, **Tyr867**, Ile879, Glu880, Ile881, **Val882**, Ile963	δBzo1: Trp760, **Tyr813**, Ile825, **Val828**, **Thr833**, Met900, Ile910
**D6**	9.195	7.057	6.579	7.640	αBzo2: Met772, Ile800, **Tyr836**, Ile848, **Val851**, **Ser854**, **Gln859**, Met922	βBzo4: **Lys775**, Met779, **Trp787**, Ile803, Val853, **Val854**, Met926	γBzo2: Trp812, Ile831, **Lys833**, Asp841, **Tyr867**, Ile879, Glu880, Ile881, **Val882**, Ile963	δBzo1: Trp760, Ile825, Ile825, **Val828**, Met900, Ile910
**D7**	9.286	7.395	7.154	8.047	αBzo2: Met772, Ile800, Ile848, **Val851**, **Ser854**, **Gln859**, Met922	βBzo1: **Trp787**, Val853, **Val854**, Met926	γBzo3: Trp812, **Tyr867**, Ile879, **Val882**, Ile963	δBzo1: **Tyr813**, Ile825, **Val828**, Met900, Ile910
**D8**	9.356	7.043	6.057	7.884	αBzo2: Ile800, **Asp810**, Ile848, **Val851**, **Ser854**, **Gln859**, Met922	βBzo4: **Lys775**, **Trp787**, **Glu852**, Val853, **Val854**, Met926	γBzo3: Trp812, **Asp841**, **Tyr867**, Ile879, **Val882**, **Met953**, Ile963	δBzo1: **Lys779**, **Asp787**, Ile825, **Val828**, Met900, Ile910
**D9**	9.272	7.260	6.548	8.067	αBzo2: Ile800, Ile848, **Val851**, **Ser854**, **Gln859**, Met922, **Asp933**	βBzo1: **Lys775**, **Trp787**, Val853, **Val854**, Met926	γBzo3: Trp812, **Tyr867**, Ile879, **Val882**, Ile963	δBzo1: Trp760, **Lys779**, Ile825, Val828, Met900, Ile910
**D10**	9.009	7.197	7.279	7.351	αBzo2: Ile800, Ile848, **Val851**, **Ser854**, **Gln859**, Met922	βBzo3: **Lys775**, **Trp787**, Glu852, Val853, **Val854**, Met926	γBzo2: Trp812, Ile831, Ile879, Ile881, **Val882**, Ile963	δBzo5: Ile825, **Val828**, Met900, Ile910
**D11**	9.350	7.216	7.573	7.944	αBzo2: Met772, Ile800, Ile848, **Val851**, **Ser854**, **Gln859**, Met922	βBzo1: **Lys775**, **Trp787**, Glu852, Val853, **Val854**, Met926	γBzo3: Trp812, Ile831, **Tyr867**, Ile879, **Val882**, **Met953**, Ile963	δBzo5: Ile825, **Val828**, Met900, Ile910
**D12**	8.655	7.107	6.504	7.371	αBzo2: Met772, Ile800, **Tyr836**, Ile848, **Val851**, **Ser854**, **Gln859**, Met922	βBzo5: Met779, Ile803, Glu852, Val853, **Val854**, **Ser857**, **Glu858**	γBzo5: Ile831, Ile879, **Val882**, Met953, Ile963	δBzo1: **Lys779**, Asp787, **Tyr813**, Ile825, Glu826, **Asp897**, Met900, Ile910
**D13**	10.377	6.588	7.537	8.897	αBzo2: Ile800, **Tyr836**, Ile848, **Val851**, **Gln859**, Met922	βBzo3: **Lys775**, **Trp787**, Ile803, Val853, **Val854**, Met926	γBzo3: Trp812, Ile831, **Tyr867**, Ile879, **Val882**, **Asp884**, **Ala885**, Ile963	δBzo1: Trp760, Ile825, **Val828**, **Asp832**, **Thr833**, Met900, Ile910
**D14**	9.484	6.448	7.308	8.173	αBzo2: Ile800, Ile848, **Val851**, **Ser854**, **Gln859**, Met922	βBzo4: **Lys777**, Met779, **Trp787**, Ile803, Val853, **Val854**	γBzo3: Trp812, Ile831, **Tyr867**, Ile879, **Val882**, **Asp884**, **Ala885**, Ile963	δBzo1: Trp760, **Lys779**, Ile825, **Val828**, Met900, Ile910

**D15**	8.033	6.693	4.944	6.439	αBzo2: Asp810, **Tyr836**, Ile848, **Val851**, **Ser854**, **Gln859**, Met922	βBzo4: **Trp787**, Ile803, Val853, **Val854**, Met926	γBzo2: Trp812, Ile831, **Lys833**, Ile879, Ile881, **Val882**, **Ala885**	δBzo1: Trp760, **Lys779**, Ile825, Val828, **Ser831**, **Asp832**, Ile910
**D16**	8.317	6.660	5.918	7.051	αBzo2: Met772, Pro778, Ile800, Asp810, Ile848, **Val851**, **Ser854**, **Gln859**, Met922, **Asp933**	βBzo4: **Lys777**, Met779, Trp787, Ile803, Val853, **Val854**, Met926	γBzo2: Trp812, Ile831, **Lys833**, Ile879, **Val882**, **Ala885**, Ile963	δBzo1: Trp760, Ile825, **Val828**, **Asp832**, **Tyr836**, Met900, Ile910
**D17**	8.200	6.290	5.300	6.983	αBzo2: Ile800, Asp810, **Tyr836**, Ile848, Val850, **Val851**, **Ser854**, **Gln859**, Met922	βBzo3: **Lys775**, **Trp787**, Val853, **Val854**, Met926	γBzo3: Trp812, Asp841, **Tyr867**, Ile879, **Val882**, **Ala885**, Ile963	δBzo1: Trp760, **Lys779**, Ile825, **Val828**, **Asp832**, Met900, Ile910
**D18**	8.271	6.920	6.145	7.152	αBzo2: Met772, Ile800, Ile848, **Val851**, **Ser854**, **Gln859**, Met922	βBzo5: Met779, Ile803, Glu852, **Val854**, **Thr856**, **Ser857**, Met926	γBzo4: Trp812, Ile831, **Lys833**, Ile879, **Val882**, Ile963, Asp964	δBzo1: **Lys779**, **Tyr813**, Ile825, **Val828**, Met900, Ile910, Ile910
**D19**	8.112	6.896	5.072	6.762	αBzo2: Ile800, Ile848, **Val851**, **Ser854**, **Gln859**, Met922	βBzo3: Met779, Trp787, Ile803, Val853, **Val854**, **Ser857**, **Glu858**	γBzo4: Trp812, Ile831, **Lys833**, Ile879, **Val882**, **Ala885**, Met953, Ile963, **Asp964**	δBzo1: **Lys779**, **Asp787**, **Tyr813**, Ile825, Met900, Ile910
**D20**	7.699	6.099	5.004	6.382	αBzo2: Met772, Ile848, **Val851**, **Ser854**, **Gln859**, Met922, **Asp933**	βBzo3: **Lys739**, Met779, Trp787, Val853, **Glu858**	γBzo4: Ile831, **Lys833**, Ile879, Val882, Met953, Ile963, Asp964	δBzo1: **Lys779**, **Tyr813**, Ile825, Met900, Ile910
**D21**	8.140	6.581	5.711	6.898	αBzo2: Met772, Ile848, Val850, **Val851**, **Ser854**, **Gln859**	βBzo3: Met779, Val853, **Val854**, **Ser857**, Met926	γBzo3: Ile879, Glu880, **Val882**, Lys883, **Asp885**, Met953, Ile963	δBzo1: Trp760, Ile825, **Val828**, **Ser831**, Met900, Ile910
**D22**	8.143	6.655	5.822	6.968	αBzo2: Met772, **Trp780**, Ile800, Ile848, Glu849, Val850, **Val851**, **Ser854**, Met922	βBzo3: **Trp787**, Glu852, Val853, **Val854**, Met926	γBzo5: Ile879, Glu880, **Val882**, **Ala885**, Met953, Ile963	δBzo1: Trp760, Ile825, **Val828**, Ile910
**D23**	8.621	6.738	5.233	7.011	αBzo2: Met772, Pro778, Ile800, Ile848, Val850, **Val851**, **Ser854**, **Gln859**, Met922	βBzo3: Met779, **Trp787**, Ile803, Glu852, Val853, **Val854**, Met926	γBzo3: Trp812, Ile831, **Tyr867**, Ile879, Glu880, **Val882**, **Ala885**, Ile963	δBzo1: Trp760, **Lys779**, Ile825, **Val828**, Met900, Ile910

Figure S13. The Williams plots (defined AD domains) for the designed compounds of PI3Kα, PI3Kβ, PI3Kγ, and PI3Kδ 3D-QSAR models.

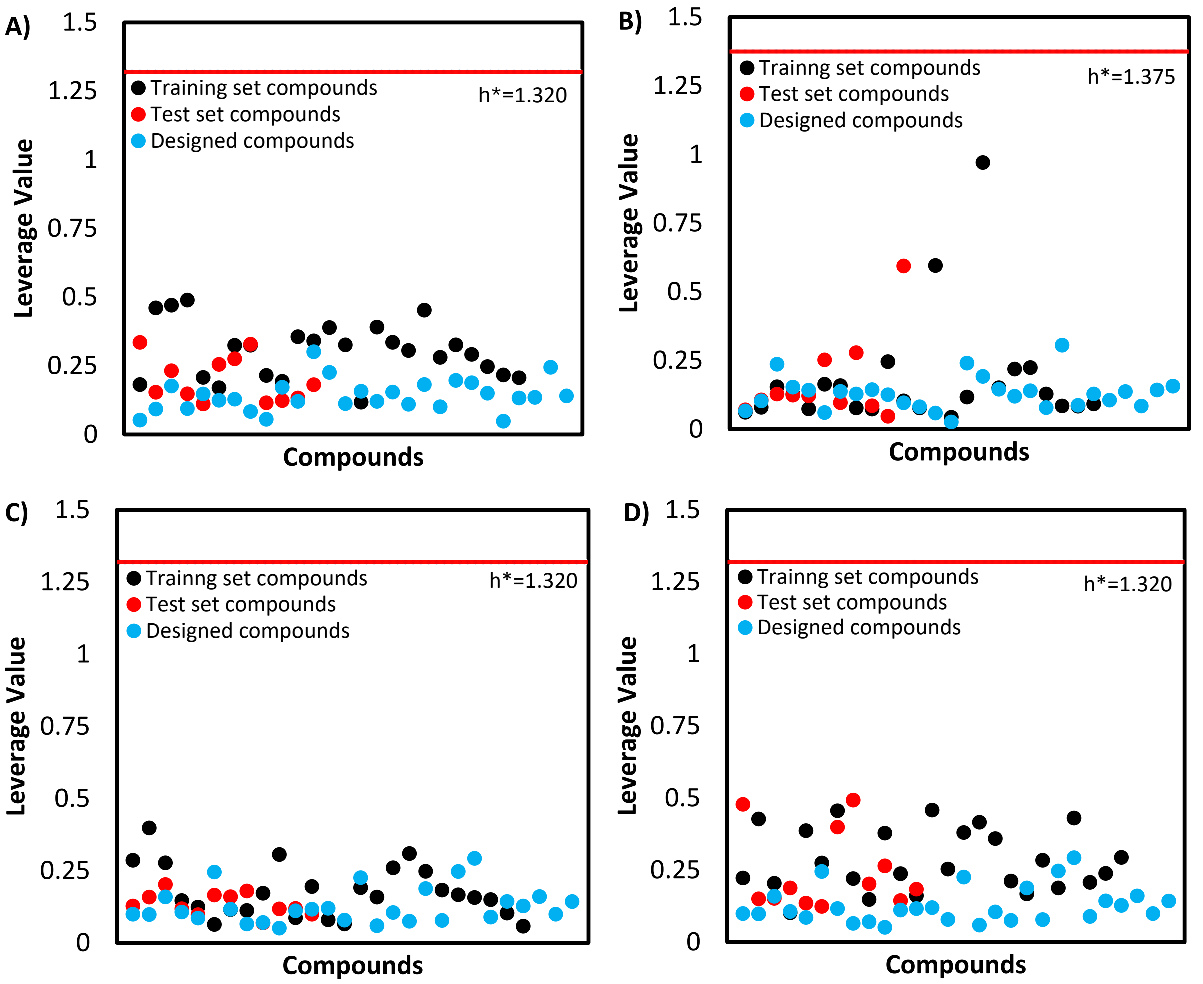

Figure S14. Structures of compounds selected from the SBVS procedure.

VS1

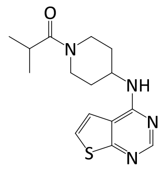

VS2

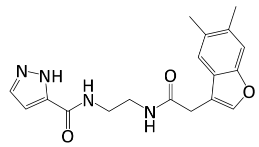

VS3

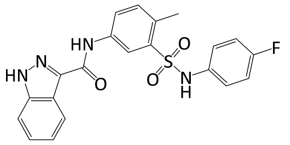

VS4

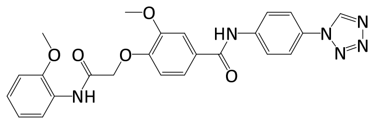

VS5

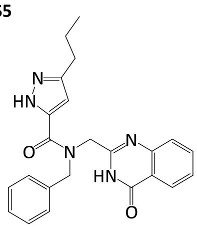

VS6

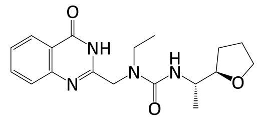

VS7

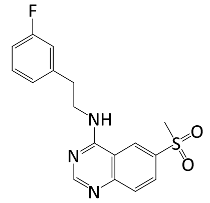

VS8

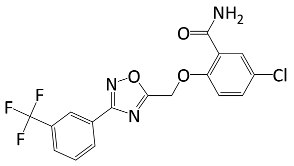

Table S10. Predicted pKi values, selected PI3K centroids, and key binding site interactions for the compounds obtained from the SBVS procedure; amino acids forming hydrogen bonds are bolded.

Manuscript ID	Enamine database ID	LDA score	Predicted pK_i values				Binding site interactions			
			PI3Kα	PI3Kβ	PI3Kσ	PI3Kδ	PI3Kα	PI3Kβ	PI3Kγ	PI3Kδ
**B1** (Lead)	/	/	10.523	8.253	8.697	9.654	αBzo2: Ile800, Ile848, **Val851**, **Ser854**, **Gln859**, Met922	βBzo4: **Lys775**, **Trp787**, Glu852, Val853, **Val854**	γBzo3: Tyr867, Ile879, Glu880, Ile881, **Val882**, Met953	δBzo1: Trp760, Asp787, **Lys779**, Ile825, **Val828**, Met900, Ile910
**VS1**	Z354333368	3.801	8.292	6.160	7.458	6.993	αBzo2: **Gln859**, **Val851**, Val850, Met922, Ile932, Tyr836	βBzo2: **Val854**, Val853, Met926, Ile936	γThz4: **Lys833**, **Val882**, Asp964, Ile831, Ile879, Ile963, Met953	δBzo1: **Val828**, **Asn836**, **Arg830**, Ile910, Met900, Ile825, Trp760, Tyr813
**VS2**	Z1633943065	2.983	7.385	5.664	6.556	6.106	αThz4: **Val851**, **Ser854**, **Asn853**, Ile800, Ile848, Val850, Met922, Ile932	βBzo5: **Val854**, Val853, Met779, Trp787, Ile803	γBzo3: **Val882**, **Ala885**, Ile831, Ile879, Ile963	δBzo3: **Val828**, **Ser831**, Ile910, Met900, Met752
**VS3**	Z43963691	2.879	6.788	5.721	6.170	6.647	αBzo2: **Gln859**, **Ser854**, **Val851**, Val850, Met922, Tyr836	βBzo3: **Val854**, **Trp787**, Met926, Ile803	γBzo5: **Lys833**, **Asp964**, Ile831, Ile879, Ile963	δBzo1: **Val828**, **Asn836**, **Glu826**, Ile910, Met900, Ile825, Trp760, Tyr813
**VS4**	Z26552865	2.469	7.927	6.631	7.185	8.566	αBzo2: **Val851**, Arg852, **Asn853**, **His855**, Ile800, Ile848, Val850, Ile932	βBzo3: **Val854**, Val853, **Ser857**, **Lys929**, Met926, Ile803	γBzo5: **Asp884**, **Val882**, Ala885, Ile879, Ile963, Met953	δBzo1: **Val828**, **Arg830**, Ile910, Met900, Ile825, Trp760, Tyr813
**VS5**	Z298834140	2.356	7.547	6.356	6.986	7.848	αBzo2: **Val851**, **Asn853**, Ile800, Ile848, Val850, Met922, Ile932	βBzo4: **Val853**, **Ser857**, Val853, Met779, Trp787, Ile803	γThz4: **Lys833**, **Asp964**, Ile831, Ile879, Ile963	δBzo4: **Val828**, Ile910, Met900, Ile825, Trp760, Tyr813
**VS6**	Z535396046	2.173	6.437	6.445	7.057	7.278	αBzo2: **Val851**, **Ser854**, **Arg770**, Trp780, Val850, Met922, Ile932, Tyr836	βBzo5: **Val854**, **Ser857**, **Trp787**, Ile803	γThz5: **Val882**, **Asp964**, Ile831, Ile879, Ile963, Met953, Trp812	δBzo4: **Val828**, Ser831, Ile910, Met900, Ile825, Trp760
**VS7**	Z1823628270	2.128	7.209	6.009	7.411	7.329	αThz4: **Val851**, **Ser854**, **Asn853**, Val850, Ile932, Tyr836	βBzo3: Val853, Lys777, Lys775, Trp787, Met779	γBzo2: Val882, **Tyr867**, Ile831, Ile879, Ile963, Met953, Trp812	δBzo1: **Val828**, Glu826, Ile910, Met900, Ile825, Trp760, Tyr813
**VS8**	Z226221758	2.056	7.158	6.148	6.635	7.142	αBzo2: **Val851**, Gln859, Ser854, Ile800, Ile848, Val850, Ile932, Tyr836	βBzo2: **Ser857**, Trp787, Lys777, Ile936, Ile803	γBzo3: **Val882**, **Asp964**, Ile879, Ile963	δBzo5: **Val828**, **Glu826**, Ile910, Met900, Ile825, Trp760

Figure S15. The Williams plots (defined AD domains) of compounds obtained from the LDA-SBVS procedure for PI3Kα, PI3Kβ, PI3Kγ, and PI3Kδ 3D-QSAR models.

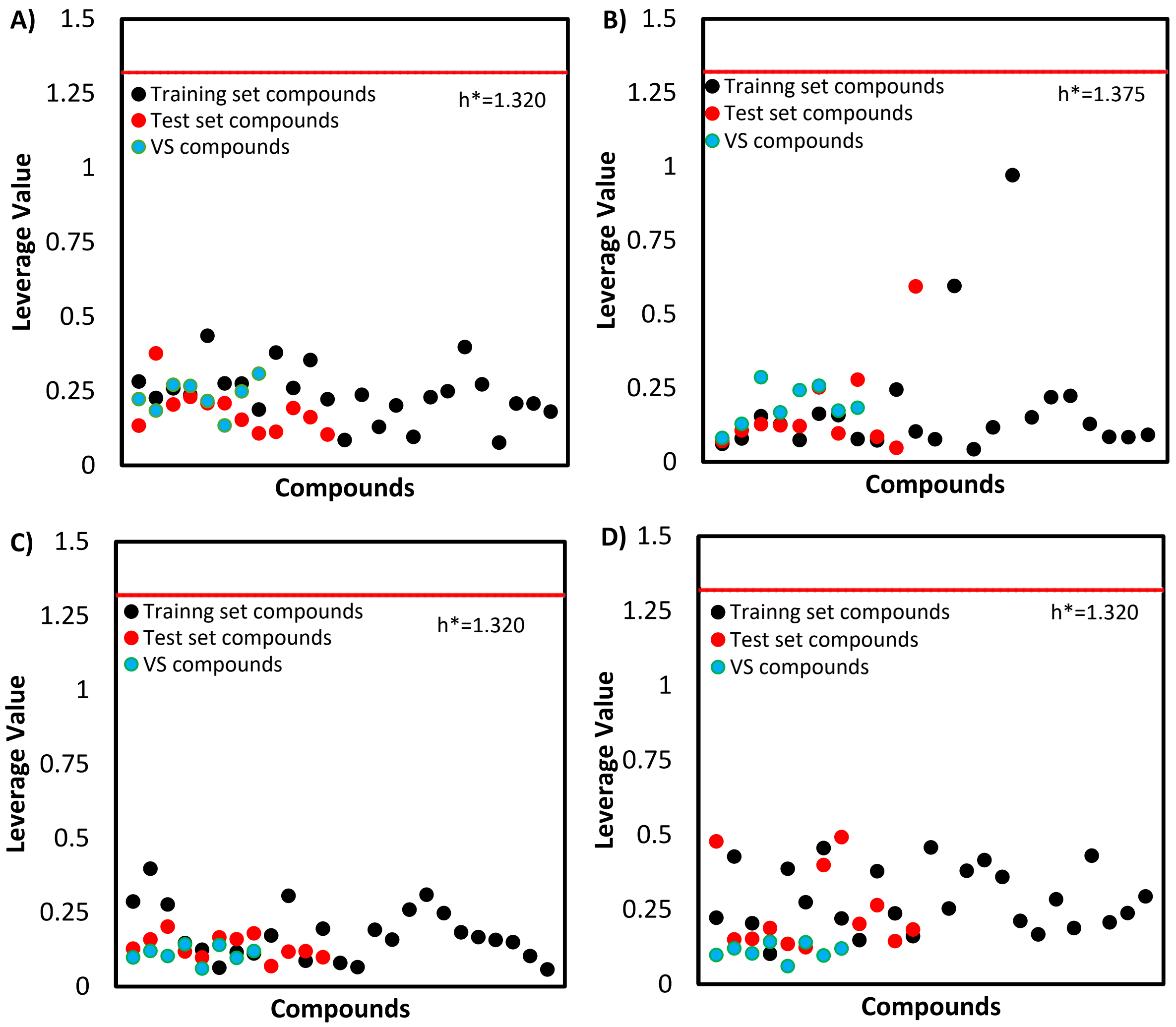

Table S11. Physicochemical properties and synthetic accessibility scores of designed and VS compounds obtained with SwissADME server.

Identifier	MW	MLOGP	Lipinski #violations	TPSA	Synthetic Accessibility
**B1** (Lead)	407.47	1.23	0	104.09	4.16
**H1**	401.37	0.78	0	114.95	3.85
**H2**	393.44	1	0	114.95	4.08
**H3**	409.44	0.23	0	118.61	4.11
**H4**	397.45	0.81	0	119.03	3.87
**H5**	425.46	1.07	0	114.95	4.14
**H6**	407.47	0.96	0	124.94	4.12
**D1**	437.49	1.08	0	114.95	4.33
**D2**	429.42	1.63	0	114.95	4.11
**D3**	411.43	1.52	0	114.95	4.11
**D4**	397.41	1.22	0	114.95	3.3
**D5**	393.44	0.73	0	135.18	3.97
**D6**	437.54	1.39	0	114.95	4.03
**D7**	401.4	1.06	1	114.95	3.76
**D8**	437.54	2.07	0	114.95	4.12
**D9**	387.37	1.5	1	135.18	3.74
**D10**	425.48	1.41	0	114.95	4.38
**D11**	381.43	2.06	0	114.95	3.48
**D12**	397.43	1.56	0	119.03	3.57
**D13**	397.43	1.29	0	114.95	3.58
**D14**	391.39	1.22	1	154.28	3.73
**D15**	397.43	0.55	0	114.95	4.12
**D16**	421.5	1.18	0	154.28	4.23
**D17**	389.36	1.09	0	143.97	3.77
**D18**	399.45	1.34	0	127.82	3.9
**D19**	393.44	1.34	0	148.05	3.32
**D20**	395.46	1	0	148.05	3.69
**D21**	408.46	0.6	0	178.17	3.52
**D22**	393.44	1	0	143.97	4.07
**D23**	401.37	0.78	0	123.74	3.81
**VS1**	304.41	1.74	0	58.12	2.78
**VS2**	340.38	0.72	0	100.02	2.98
**VS3**	424.45	2.41	0	103.95	2.96
**VS4**	474.47	2.04	1	129.49	3.19
**VS5**	401.46	2.67	0	94.74	3.05
**VS6**	344.41	1.69	0	87.32	3.67
**VS7**	345.39	2.95	0	71.95	2.41
**VS8**	397.74	2.81	0	91.24	3.03

Table S12. Pharmacokinetic properties of designed and VS compounds obtained with ADMETPredictor software.

Identifier	BBB_Filter	LogBB	hum_fup %	Pgp_Substr	CYP_Risk	hERG_Filter	TOX_Risk
**B1** (Lead)	Low (40%)	-1.246	23.845	Yes (88%)	0.422	No (66%)	1.5
**H1**	Low (77%)	-1.496	21.58	Yes (99%)	0	No (51%)	1.5
**H2**	Low (73%)	-1.315	14.799	Yes (99%)	0.311	Yes (57%)	1.5
**H3**	High (72%)	-0.778	37.5	Yes (99%)	0	No (91%)	1.298
**H4**	Low (68%)	-1.159	8.631	Yes (99%)	0	No (91%)	1.5
**H5**	Low (77%)	-1.432	18.804	Yes (99%)	0	No (69%)	1.463
**H6**	Low (77%)	-1.372	25.842	Yes (92%)	0	No (64%)	1
**D1**	Low (68%)	-1.506	18.962	Yes (99%)	0	No (56%)	1.5
**D2**	Low (64%)	-1.262	15.207	Yes (99%)	0	No (73%)	1.923
**D3**	Low (73%)	-1.39	11.917	Yes (99%)	1	No (73%)	1.535
**D4**	Low (68%)	-1.494	15.813	Yes (99%)	0	Yes (52%)	1.5
**D5**	Low (77%)	-1.594	24.914	Yes (99%)	0	No (91%)	1.5
**D6**	Low (77%)	-1.497	12.269	Yes (99%)	1	Yes (52%)	1.5
**D7**	Low (68%)	-1.286	16.352	Yes (99%)	0.234	No (43%)	1.555
**D8**	Low (73%)	-1.299	16.506	Yes (99%)	0	Yes (54%)	1.5
**D9**	Low (89%)	-1.406	18.129	Yes (99%)	0.255	No (73%)	1.5
**D10**	Low (68%)	-1.12	12.114	Yes (99%)	1.096	No (66%)	1.662
**D11**	Low (68%)	-1.3	17.698	Yes (99%)	1	No (61%)	1.47
**D12**	Low (60%)	-1.313	9.62	Yes (99%)	0	No (91%)	1
**D13**	Low (77%)	-1.181	11.829	Yes (99%)	0.847	Yes (82%)	1.5
**D14**	Low (97%)	-1.118	45.437	Yes (99%)	0	<u>Yes</u>	1
**D15**	Low (77%)	-1.252	11.247	Yes (99%)	0.54	Yes (86%)	1.5
**D16**	Low (89%)	-1.125	44.573	Yes (97%)	0	<u>No</u>	1
**D17**	Low (97%)	-1.57	21.559	Yes (99%)	0	No (91%)	2.5
**D18**	Low (77%)	-1.322	3.645	Yes (99%)	1	No (91%)	1.5
**D19**	Low (97%)	<u>-1.397</u>	6.146	Yes (99%)	0	No (91%)	1.5
**D20**	Low (97%)	-1.269	6.426	Yes (99%)	0	No (91%)	0.86
**D21**	Low (97%)	-0.718	67.849	Yes (99%)	0.5	<u>No</u>	1
**D22**	Low (89%)	-1.605	28.109	Yes (99%)	0	No (91%)	1.5
**D23**	Low (77%)	-1.322	17.251	Yes (99%)	0	No (79%)	1
**VS1**	High (99%)	-0.035	10.822	No (69%)	0.264	No (91%)	0.274
**VS2**	Low (60%)	-1.381	7.668	Yes (99%)	1	No (69%)	2.473
**VS3**	Low (53%)	<u>-0.679</u>	2.301	Yes (99%)	1	No (61%)	1
**VS4**	Low (73%)	-0.769	4.006	Yes (86%)	0.136	No (91%)	1.358
**VS5**	Low (60%)	-0.836	6.147	Yes (97%)	1.313	No (59%)	0.73
**VS6**	High (73%)	-0.953	36.525	Yes (95%)	1.029	No (79%)	0
**VS7**	High (99%)	-0.011	8.79	No (94%)	1	No (48%)	0
**VS8**	High (99%)	-0.496	4.825	No (74%)	0.924	Yes (98%)	0

Table S13. logPe values predicted with the PAMPA-SVM model: low permeability compounds (- 6.14 < logPe < - 5.66); medium permeability compounds (- 5.66 < logPe < - 5.33); high permeability compounds (logPe > - 5.33).

Compound	logPe
D20	-5.793
VS1	-5.748
VS8	-5.724
D18	-5.710
D17	-5.649
D22	-5.646
D19	-5.596
D23	-5.537
H5	-5.527
D16	-5.514
VS5	-5.500
VS7	-5.487
D14	-5.481
VS6	-5.421
B1 (Lead)	-5.357
D21	-5.357
VS2	-5.310
D6	-5.293
VS4	-5.220
H4	-5.199
D12	-5.199
D1	-5.184
D3	-5.184
D4	-5.184
D5	-5.184
H1	-5.166
D2	-5.166
D10	-5.147
D11	-5.147
H2	-5.131
D7	-5.131
D8	-5.131
D9	-5.131
VS3	-5.128
H6	-5.034
D15	-4.991
D13	-4.989
H3	-4.880

Figure S16. The Williams plot (defined AD domains) of the PAMPA-SVM model which includes PAMPA-SVM dataset compounds, lead compound, designed compounds and compounds obtained from the LDA-SBVS procedure.

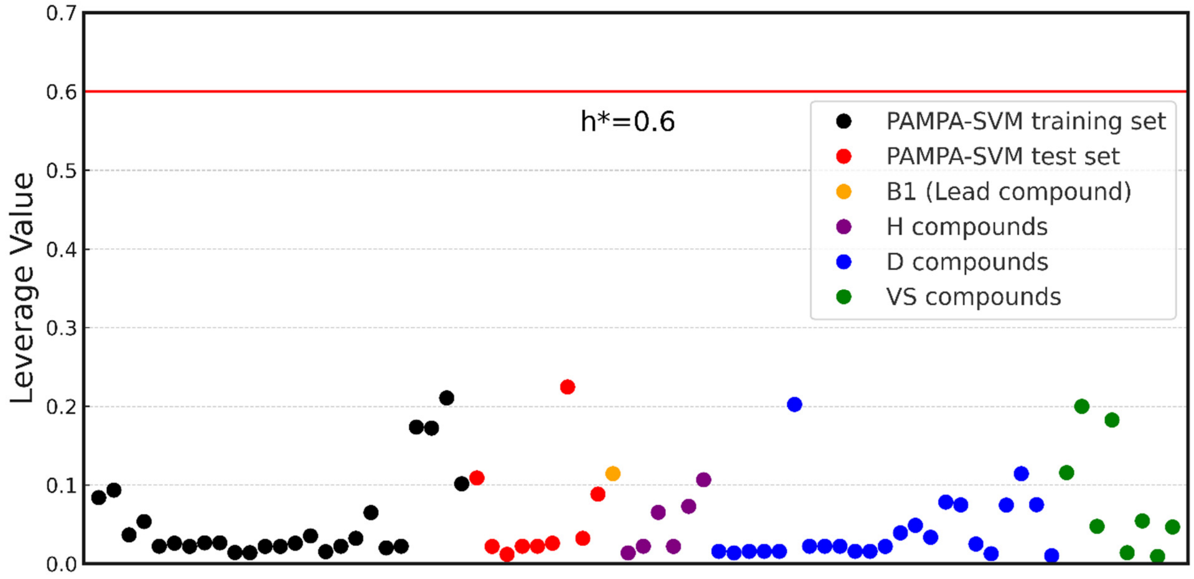

Figure S17. Self-organizing map (SOM) analysis U-matrix projection PI3Kα inhibitor chemical space.

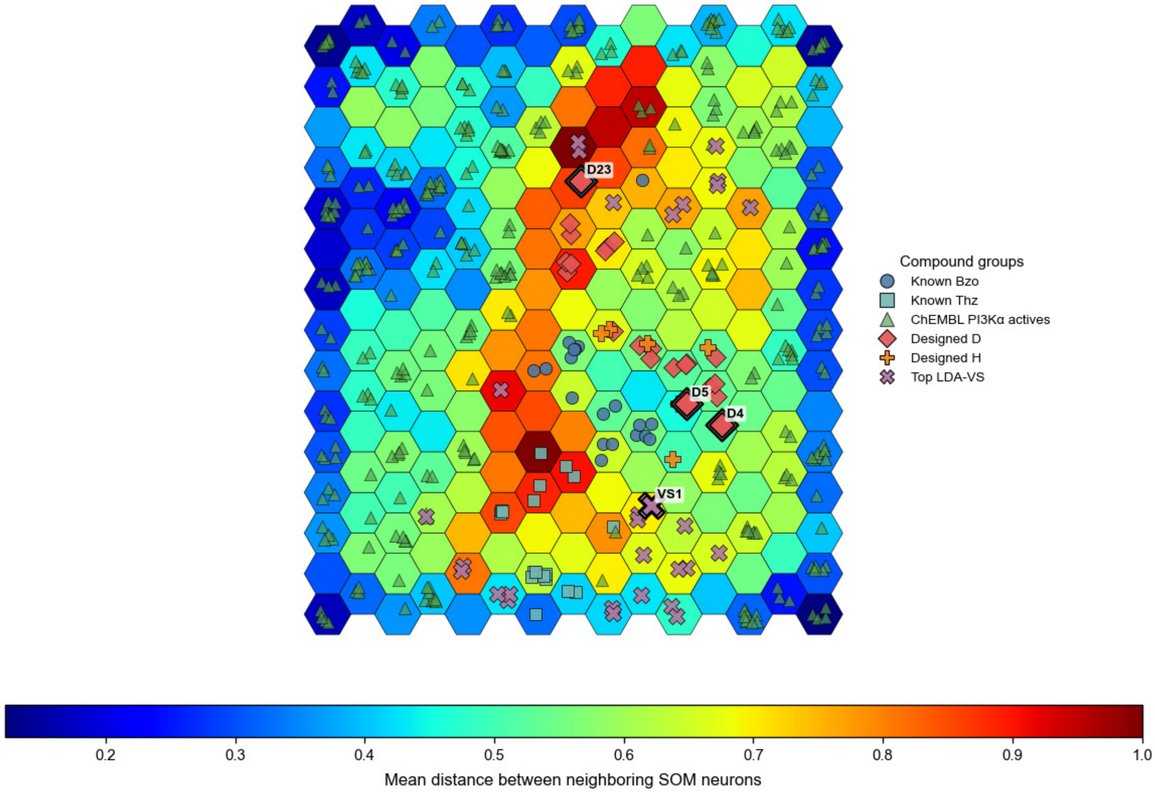

Supplementary Results S2. Comparative D23 MD analysis across PI3K isoforms

A representative 100 ns MD assessment was performed for **D23** in complex with PI3Kα, PI3Kβ, PI3Kγ, and PI3Kδ to qualitatively evaluate the stability of the predicted binding mode across class I PI3K isoforms. The trajectories were analyzed by monitoring protein backbone RMSD, active-site RMSD, ligand RMSD after backbone alignment, Cα RMSF, and radius of gyration.

The protein backbone RMSD and radius of gyration profiles indicated that all four complexes preserved overall structural stability and compactness during the simulations. Active-site RMSD profiles further suggested that the local binding-site architecture remained generally preserved. However, clear differences were observed in ligand behavior, particularly in the extent of ligand repositioning and in the preservation of canonical hinge-region anchoring.

In the PI3Kα-D23 complex, the ligand remained positioned within the ATP-binding site and preserved the main interaction features predicted by ensemble docking. The chromeno[3,4-d]imidazole hinge-binding scaffold maintained the interaction with αVal851, while the affinity pocket binder contributed to hydrophobic stabilization within the active site. The urea-based selectivity modifier was oriented toward the hinge-adjacent hydrophobic region II, where it interacted with residues such as αSer854, αHis855, αAsn853, and αArg852. The interaction between D23 and αGln859 showed a dynamic character, similar to that observed for the reference benzoxazepine compound **B1**. Together with additional contacts involving non-conserved or partially conserved residues such as αSer854, αHis855, αAsn853, and αArg852, this interaction supports positioning of the selectivity modifier within hydrophobic region II and contributes to the predicted PI3Kα-selective interaction profile.

In the PI3Kβ-D23 complex, the protein structure remained stable, but the ligand showed more pronounced repositioning relative to the initial binding mode. The hydrogen bond with βVal854 in the hinge region was less consistently preserved than in PI3Kα, suggesting a weaker anchoring to the hinge region. **D23** instead formed interactions with residues such as βLys777, βGlu858, βSer857, βSer855, βTrp787, and βMet926. This interaction pattern indicates that **D23** can remain within the PI3Kβ binding site, but with less favorable interactions for PI3Kβ inhibitory activity.

In the PI3Kγ-D23 complex, **D23** displayed an initial adjustment but subsequently retained a relatively stable binding orientation. The ligand preserved a hydrogen bond with γVal882 in the hinge region, indicating that the conserved interaction can also be maintained in this isoform. Additional interactions with residues such as γTrp812, γIle831, γIle881, γAla885, γMet953, γIle963, and γAsp964 contributed to ligand stabilization within the PI3Kγ active site. This behavior suggests that selectivity over PI3Kγ cannot be explained solely by the hinge interaction and should instead be interpreted from the broader interaction pattern and 3D-QSAR-derived selectivity features.

In the PI3Kδ-D23 complex, the protein backbone and active-site region remained stable, but the ligand showed more pronounced repositioning compared with PI3Kα. The hydrogen bond with δVal828 from the hinge region was less stabilized, suggesting a reduced ability to maintain the PI3Kα-like hinge-binding positioning. **D23** formed interactions with residues such as δMet752, δTrp760, δIle777, δVal828, δThr833, δAsn836, δMet900, δIle910, and δAsp911, indicating that the compound remained within the binding pocket but adopted a different interaction pattern compared with PI3Kα.

Overall, the comparative MD analysis supports the stability of the predicted **D23** binding mode in PI3Kα and highlights isoform-dependent differences in ligand positioning. PI3Kβ and PI3Kδ showed less stable preservation of the canonical PI3Kα-like hinge-binding pattern, whereas PI3Kγ retained stable hinge binding. Therefore, the predicted PI3Kα preference of **D23** should be interpreted as the result of the combined contribution of hinge binding, affinity pocket occupation, selectivity modifier positioning in the isoform-variable hydrophobic region II, and the ligand-based 3D-QSAR selectivity profile.

Figure S18. Backbone (A), active-site (B), and ligand (C) RMSD profiles of D23 in complex with PI3Kα, PI3Kβ, PI3Kγ, and PI3Kδ during 100 ns MD simulations.

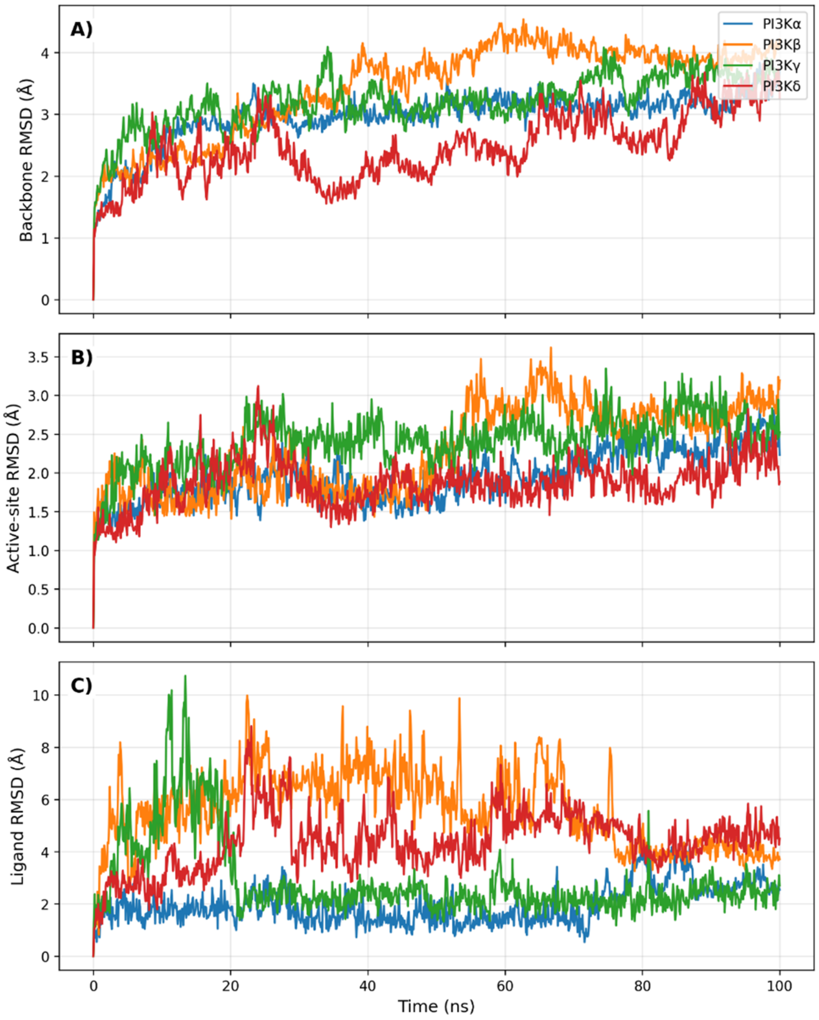

Figure S19. Cα RMSF profiles of PI3Kα (A), PI3Kβ (B), PI3Kγ (C), and PI3Kδ (D) in complex with D23 during 100 ns MD simulations.

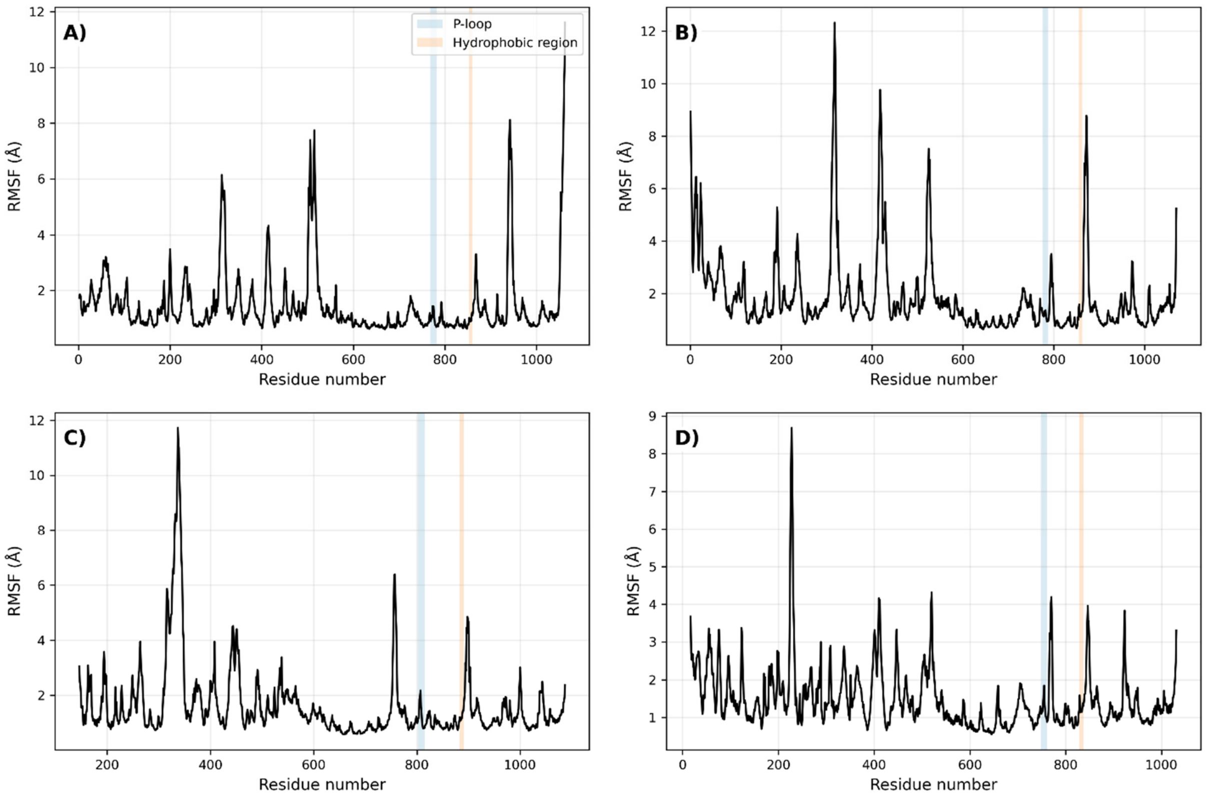

Figure S20. Radius of gyration profiles of PI3Kα, PI3Kβ, PI3Kγ, and PI3Kδ in complex with D23 during 100 ns MD simulations.

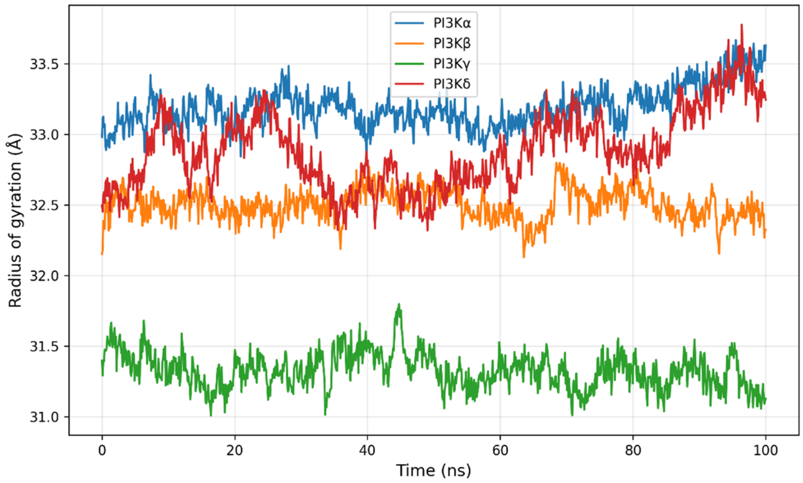

Table S14. Bzo cluster dataset compounds and their pKi values for PI3Kα, PI3Kβ, PI3Kγ, and PI3Kδ isoforms.

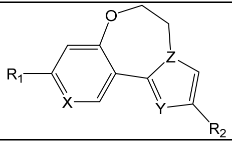

Manuscript ID	ChEMBL ID	X	Y	Z	R_1	R_2	PI3Kα pK_i	PI3Kβ pK_i	PI3Kγ pK_i	PI3Kδ pK_i
**B1**	ChEMBL3770993	C	N	N	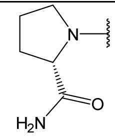	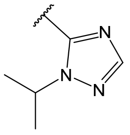	10.523	8.253	8.697	9.654
**B2**	ChEMBL3770306	C	N	N	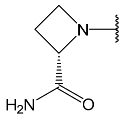	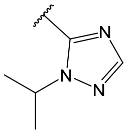	10.000	8.161	8.328	9.137
**B3**	ChEMBL3770709	N	N	N	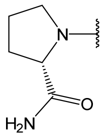	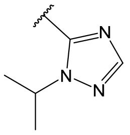	10.000	7.550	7.955	8.569
**B4**	ChEMBL3770325	N	N	N	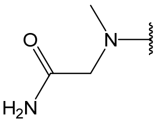	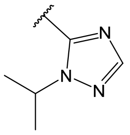	9.699	7.569	8.046	8.699
**B5**	ChEMBL3771364	C	N	N	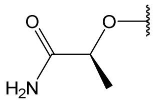	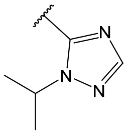	9.699	7.575	7.991	8.398
**B6**	ChEMBL3770140	C	N	N	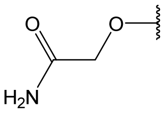	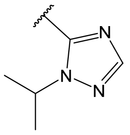	9.523	8.319	8.620	9.523
**B7**	ChEMBL3770717	C	N	N	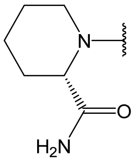	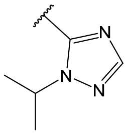	9.523	7.007	7.327	8.319
**B8**	ChEMBL2387080	C	N	N	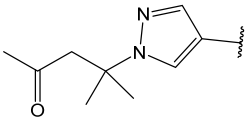	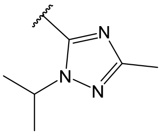	9.523	8.032	9.004	9.921
**B9**	ChEMBL3769854	C	N	N	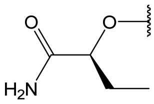	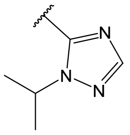	9.301	7.086	7.577	8.071
**B10**	ChEMBL3769966	N	N	N	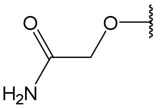	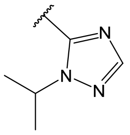	9.046	7.393	8.233	8.321

**B11**	ChEMBL3770332	C	S	C	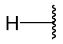	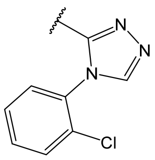	9.000	7.284	8.886	8.745
**B12**	ChEMBL3770824	N	N	N	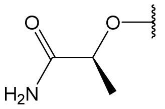	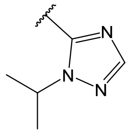	9.000	6.857	7.602	8.066
**B13**	ChEMBL3770630	N	N	N	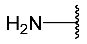	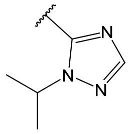	8.921	7.807	9.018	8.642
**B14**	ChEMBL3770092	N	N	N	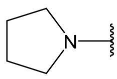	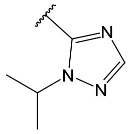	8.886	7.886	9.187	9.041
**B15**	ChEMBL3769972	N	N	N	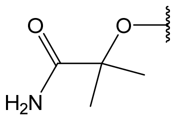	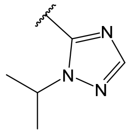	8.509	7.147	7.395	8.147
**B16**	ChEMBL3769603	N	N	N	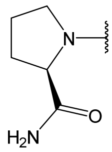	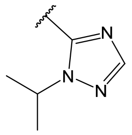	8.495	6.696	7.173	8.349
**B17**	ChEMBL3770615	N	N	N	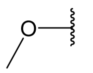	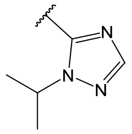	8.444	7.365	8.489	8.489
**B18**	ChEMBL3770644	C	N	N	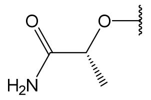	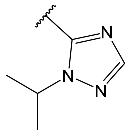	8.444	7.165	8.330	8.444
**B19**	ChEMBL3769857	N	N	N	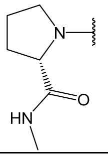	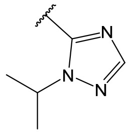	7.523	6.268	6.979	7.921

Table S15. Thz cluster dataset compounds and their pKi values for PI3Kα, PI3Kβ, PI3Kγ, and PI3Kδ isoforms.

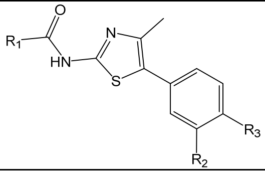

Manuscript ID	ChEMBL ID	R_1	R_2	R_3	PI3Kα pK_i	PI3Kβ pK_i	PI3Kγ pK_i	PI3Kδ pK_i
**T1**	ChEMBL2071332	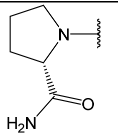	H	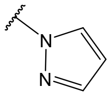	8.301	5.699	6.276	6.658
**T2**	ChEMBL2071333	H_3C	Br	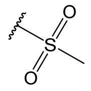	7.959	6.260	7.495	7.495
**T3**	ChEMBL2071328	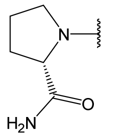	CF_3	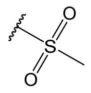	7.699	>5.000	5.943	5.943
**T4**	ChEMBL2071331	H_3C	CF_3	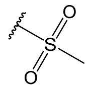	7.553	6.060	7.215	7.215
**T5**	ChEMBL2071336	H_3C	H	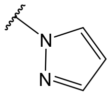	7.523	5.845	6.959	6.959
**T6**	ChEMBL2071326	H_3C	Cl	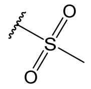	7.456	5.886	7.244	7.244
**T7**	ChEMBL2071340	H_3C	CN	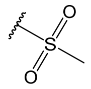	7.347	6.119	7.208	7.208
**T8**	ChEMBL2071330	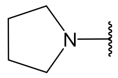	F	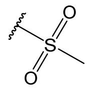	7.301	>5.000	5.236	5.236
**T9**	ChEMBL1986603	H_3C	H	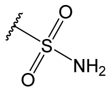	6.959	5.712	5.514	5.514
**T10**	ChEMBL2071335	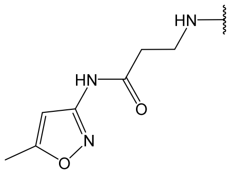	F	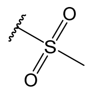	6.959	6.066	6.921	8.523
**T11**	ChEMBL2071334	H_3C	F	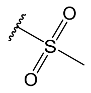	6.914	5.851	6.738	6.914
**T12**	ChEMBL2071327	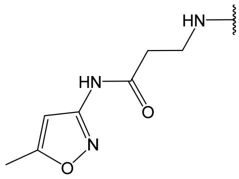	H	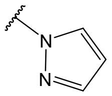	6.824	6.469	5.991	8.398

**T13**	ChEMBL2071342	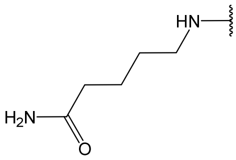	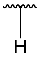	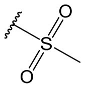	6.728	>5.000	6.585	5.441
**T14**	ChEMBL2069328	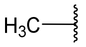	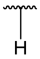	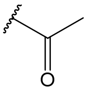	6.721	6.387	6.959	6.721
**T15**	ChEMBL2071341	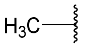	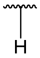	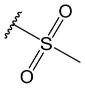	6.699	5.866	6.638	6.699
**T16**	ChEMBL2071332	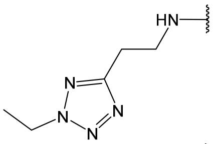	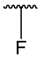	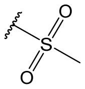	5.854	6.495	7.444	6.328
**T17**	ChEMBL2071333	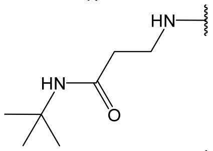	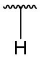	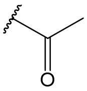	5.724	6.602	7.056	6.131
**T18**	ChEMBL2071328	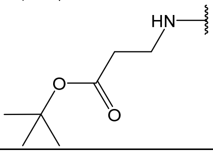	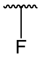	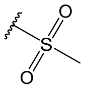	5.672	7.046	7.699	6.420

Supplementary Methods S1. Homology modeling and conformational ensemble generation

The homology model of the human p110β catalytic subunit and the addition of non-terminal missing residues in the experimental PI3K structures were performed using MODELLER 10.1 through the Chimera 1.17.3 interface. One residue adjacent to missing regions was allowed to move by default. The DOPE-HR loop modeling protocol was used, and 1000 models were generated. The model with the lowest zDOPE score was selected for further preparation. The PI3Kβ homology model was evaluated using PROCHECK, ERRAT, and ProSA-web. PI3Kα, PI3Kγ, and PI3Kδ X-ray structures and the PI3Kβ homology model were protonated at physiological pH 7.4 using the PlayMolecule server.

Initial docking was performed to generate starting complexes for conformational ensemble generation. The most active benzoxazepine and thiazole derivatives in the dataset, CHEMBL3770993 and CHEMBL2071338, respectively, were used as representative ligands and are referred to as **B1** and **T1**. These compounds were docked into PI3Kα, PI3Kβ, PI3Kγ, and PI3Kδ using GOLD 2023.3. The binding site was defined within 6.0 Å of the reference ligand in the active site, and no constraints were applied. The ChemPLP scoring function was used for pose ranking. The docking procedure was validated by re-docking the crystallographic ligands and calculating heavy-atom RMSD values relative to the reference poses.

MD simulations were performed for the PI3Kα-B1, PI3Kα-T1, PI3Kβ-B1, PI3Kβ-T1, PI3Kγ-B1, PI3Kγ-T1, PI3Kδ-B1, and PI3Kδ-T1 complexes. The simulations were used for local relaxation of protein–ligand complexes and generation of representative receptor conformations for ensemble docking. System preparation was performed using CHARMM-GUI. Each complex was solvated in a rectangular box of TIP3P water molecules with an 8 Å edge distance. Sodium and chloride ions were added by the Monte Carlo method to neutralize the systems and obtain a final salt concentration of 0.15 M. The CHARMM36m force field was used for protein topology and parameters, while ligand parameters were generated using CGenFF. Before production simulations, each system was minimized for 10000 steps. NVT equilibration was performed for 250 ps, during which the temperature was increased to 310.15 K. Production simulations were then carried out for 50 ns under NPT conditions. Periodic boundary conditions and the particle mesh Ewald method were applied for long-range electrostatic interactions. Langevin temperature coupling with a friction coefficient of 1 ps−1 was used to control the temperature, while the Langevin-piston method was applied for pressure control. The timestep was 2 fs, and trajectory frames were saved every 10 ps.

The trajectories were inspected using backbone RMSD, active-site RMSD, ligand RMSD, Cα RMSF, and radius of gyration. These analyses were used only to confirm that the selected trajectory segments were suitable for extracting representative conformations for ensemble docking. Conformational clustering was performed on the locally stabilized part of each trajectory, using active-site RMSD as the clustering criterion. The centroid structures from the five most populated clusters were selected for subsequent ensemble docking and interaction analysis.

The representative designed compound selected after completion of the design workflow, D23, was additionally subjected to 100 ns MD simulations with PI3Kα, PI3Kβ, PI3Kγ, and PI3Kδ using the same general protocol described above. In contrast to the 50 ns simulations used for conformational ensemble generation, these simulations were performed as a post-design binding-mode stability assessment. Trajectory frames were saved every 100 ps, giving 1000 frames per system. These simulations were not used for conformational clustering, ensemble docking generation, model development, or compound ranking.
